# The dynamic pool of Rec8-cohesin is crucial for meiotic recombination and transcription regulation in the yeast *Saccharomyces cerevisiae*

**DOI:** 10.1016/j.jbc.2026.113064

**Published:** 2026-04-24

**Authors:** Sheetal Paliwal, Partha Dey, Akriti Kumari, Kirithika Sadasivam, Sameer Joshi, Ritika Raghuvanshi, Rohit Goyal, Kaustuv Sanyal, K.T. Nishant, Akira Shinohara, Gunjan Mehta

**Affiliations:** 1 Laboratory of Chromosome Dynamics and Gene Regulation, Department of Biotechnology, Indian Institute of Technology Hyderabad, Sangareddy, Telangana, India; 2 School of Biology, Indian Institute of Science Education and Research Thiruvananthapuram, Trivandrum, Kerala, India; 3 Jawaharlal Nehru Centre for Advanced Scientific Research, Bengaluru, Karnataka, India; 4 Institute of Protein Research, The University of Osaka, Osaka, Japan

**Keywords:** meiosis, cohesin, Rec8, yeast, chromosome segregation, recombination

## Abstract

Cohesin is a ring-shaped protein complex that mediates sister-chromatid cohesion (SCC) to ensure accurate chromosome segregation during mitosis and meiosis. In *Saccharomyces cerevisiae*, cohesin consists of four core subunits—Smc1, Smc3, Scc1/Mcd1, and Scc3. During meiosis, the mitotic α-kleisin Scc1/Mcd1 is replaced by the meiosis-specific α-kleisin Rec8. Rec8-containing cohesin is essential for multiple meiotic processes, including chromosome morphogenesis, homologous recombination, axis and synaptonemal complex formation, SCC, and transcriptional regulation. While stable association of Rec8-cohesin with chromatin is required to maintain SCC from premeiotic S phase through anaphase II, dynamic chromatin association is thought to underlie its roles in recombination, chromosome architecture, and transcription *via* loop extrusion. Whether distinct stable and dynamic pools of Rec8-cohesin coexist during meiosis and how their functions are partitioned remained unclear. Here, we employed an anchor-away strategy to conditionally deplete only the dynamic pool of Rec8-cohesin from the nucleus while preserving the stable pool. Selective depletion reduced sporulation efficiency and spore viability without compromising SCC. Calibrated ChIP-seq revealed a genome-wide reduction in Rec8-cohesin levels rather than locus-specific loss. Functional analyses demonstrated that the dynamic pool of Rec8-cohesin is required for efficient meiotic recombination, establishment of meiosis-specific chromosome architecture and synaptonemal complex formation, and proper transcriptional regulation of key meiotic regulators. In contrast, the stable pool alone was sufficient to maintain spindle pole body cohesion. Together, our findings demonstrate the existence of two functionally distinct pools of Rec8-cohesin during yeast meiosis.

Cohesin is a highly conserved ring-shaped protein complex, composed of four core subunits in *Saccharomyces cerevisiae*: Smc1, Smc3, Scc1/Mcd1 (α-kleisin), and Scc3/Irr1 (1) (Fig. 1*A*). During meiosis, the Scc1/Mcd1 is replaced by a meiosis-specific α-kleisin, Rec8. The canonical function of the cohesin ring is to hold the sister chromatids together until anaphase of mitosis and anaphase II of meiosis, known as sister-chromatid cohesion (2) (SCC, Fig. 1*A*). In addition to SCC, cohesin mediates loop extrusion (3, 4, 5), which is important for bringing together enhancers and their cognate promoters for regulating gene expression. In meiosis, the Rec8-cohesin is a major component of chromosome axes and the assembly of a meiosis-specific ladder-like structure, the synaptonemal complex (SC; Fig. 1*B*). Several reports indicate that only a small fraction of Rec8-cohesin binds chromatin stably for SCC, whereas a substantial pool of dynamic cohesin is required for gene expression and genome organization (6, 7, 8, 9). Thus, it remains unresolved whether meiotic yeast cells contain two pools of cohesin (stable and dynamic) and, if so, what are their functions? Previous reports have shown the role of Rec8-cohesin for multiple meiotic processes, including meiotic recombination, chromosome pairing, and synapsis (10, 11, 12). However, it is not known which pool of Rec8-cohesin (stable or dynamic) is required for these prophase I-specific meiotic processes.

**Figure 1:**
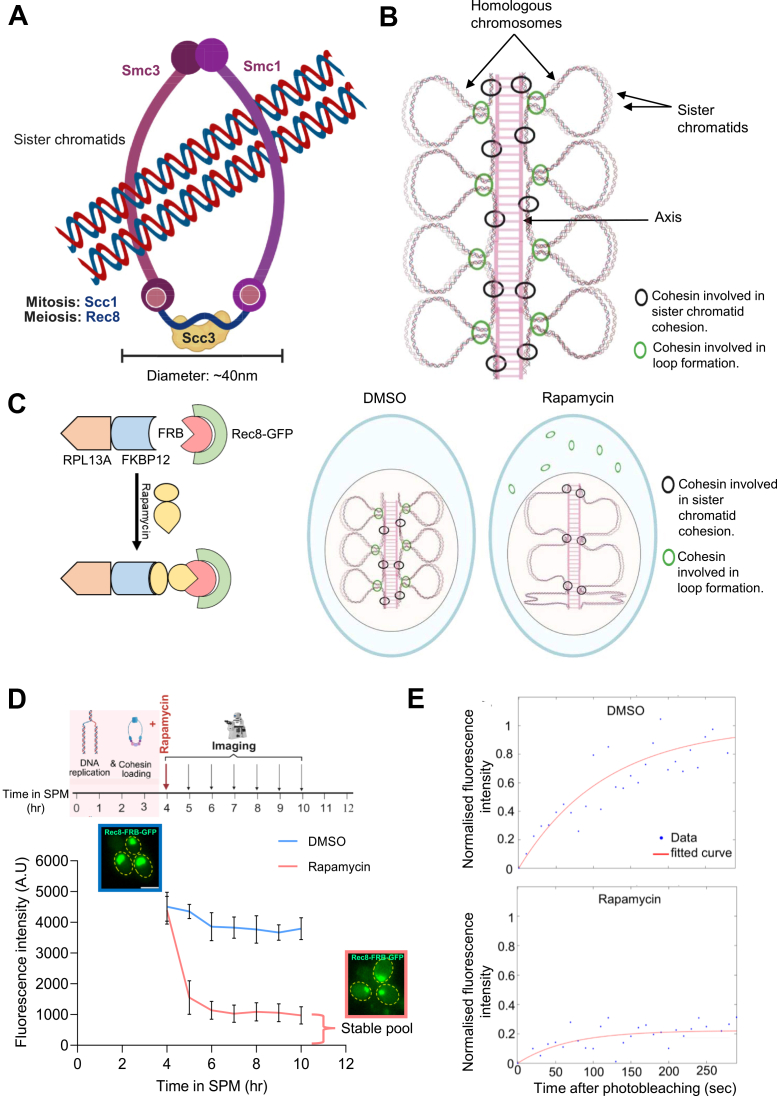
**Cohesin architecture, experiment design and validation for conditional depletion of the dynamic pool of Rec8-cohesin.***A*, architecture of the *S. cerevisiae* cohesin ring. The cohesin complex consists of Smc1, Smc3, Scc3, and Rec8 (in meiosis), forming a ring-like structure that topologically encircles sister chromatids to provide cohesion between them, known as sister-chromatid cohesion (SCC). *B*, synaptonemal complex assembly during meiotic prophase I for the pairing of homologous chromosomes. The cohesion rings shown in black are required for SCC, whereas the cohesion rings shown in green are required for loop extrusion. *C*, mechanism of anchor-away-based nuclear depletion of the Rec8-cohesin rings. We endogenously fused the *REC8* gene with FRB, and *RPL13A* with *FKBP12*. The large flux of ribosomal protein RPL13A transits the nucleus during its assembly process to the 60S particles and makes a ternary complex with rapamycin and FRB to deplete the dynamic pool of Rec8-cohesin (*green rings*). The stable pool of Rec8-cohesin (*black rings*) cannot be pulled out of the nucleus by this system, as it is topologically entrapped with the sister chromatids. *D*, Live cell imaging to quantify the depletion of the dynamic pool of Rec8-cohesin after rapamycin addition. Inset images show the nuclear and cytoplasmic GFP signal (Rec8-FRB-GFP) at the same scale before adding rapamycin (3 h in SPM) and after treating with rapamycin for 3 h (6 h in SPM). Nuclear fluorescence intensities were measured from >100 cells (after background subtraction) using ImageJ. The experiment was performed twice. *ndt80*Δ strain (GMY035) was used for this experiment. Error bars represent standard deviation (SD). Scale: 5 μm. For the dot plot representation of this data, please see Fig. S1*B*. *E*, normalized FRAP curves for Rec8-FRB-GFP in the nucleus from pachytene arrested cells (GMY35). Fluorescence intensities were background-corrected and processed using EasyFRAP webtool (58), with double normalization applied. Mean recovery curves for each condition are plotted. n = 10 for DMSO and n = 6 for rapamycin treated samples.

Additionally, initiation and progression through meiosis are regulated by a transcriptional cascade consisting of temporal and programmed expression of early, middle, and late meiotic genes (13). The transcription factor Ime1 is the master regulator for entry into meiosis, and it activates the early meiotic genes. The transcription factors Ndt80 and Ime2 are required for the transcription of middle meiotic genes. The transcription of the late meiotic genes is regulated by the upstream regulators Ime1, Ime2, and Ndt80. There is a good correlation between the time of transcription and the meiotic functions. Early meiotic genes are induced before premeiotic DNA replication, the middle meiotic genes are induced after DNA replication but before nuclear division, and the late meiotic genes are induced at the time of spore formation (13). Interestingly, recent reports have demonstrated that ∼190 meiotic genes have 5′ extended transcripts (14, 15, 16, 17). Several reports suggest the role of cohesin in regulating transcription during mitosis in *S. cerevisiae* and other model organisms (18). Hence, it is interesting to know how the absence of the dynamic pool of Rec8-cohesin alters the meiotic transcription program.

In this study, we employed an anchor-away technique for the conditional depletion of the dynamic pool of Rec8-cohesin from the nucleus, without affecting/removing the stable pool of Rec8-cohesin. Using live-cell imaging and sister chromatid segregation assays, we validated that the anchor-away removes only the dynamic pool of Rec8-cohesin, but not the stable pool of Rec8-cohesin, and showed that the SCC and sister kinetochore monoorientation remained mostly unperturbed under this condition. The calibrated ChIP-seq analysis showed genome-wide reduction in Rec8-cohesin levels upon rapamycin addition, rather than locus-specific loss, suggesting genome-wide presence of the dynamic pool of Rec8-cohesin. The chromosome-spread assays demonstrated defects in meiotic DNA double-strand breaks (DSBs) formation, crossover formation, and synaptonemal complex assembly in the absence of the dynamic pool of Rec8-cohesin. The quantification of meiotic gene expression by RT-qPCR analysis showed altered transcription of a few middle meiotic genes in the absence of the dynamic pool of Rec8-cohesin. As Rec8-cohesin is known to be important for the spindle-pole body (SPB) cohesion and duplication (19), we tested the role of the dynamic pool of Rec8-cohesin on SPB cohesion and duplication. We failed to detect any defect in SPB cohesion and duplication after depleting the dynamic pool, suggesting that the stable pool of Rec8-cohesin is enough to maintain SPB cohesion and duplication. Hence, this study demonstrates the existence of two pools of Rec8-cohesin (stable and dynamic) during yeast meiosis and their functional significance.

## Results

### Genetic engineering of yeast strains for the conditional depletion of the dynamic pool of Rec8-cohesin from the nucleus, by keeping the stable pool intact

The anchor-away technique was developed by Haruki *et al.* in 2008 and has been successfully adopted by several labs for the rapamycin-induced conditional depletion of various yeast proteins during mitosis and meiosis (20, 21). This method relies on rapamycin-induced heterodimerization between an RPL13A-FKBP12 fusion protein and FRB-fused target protein (*i.e.* Rec8, where, RPL13A is a gene that codes for the ribosomal large subunit (60S) protein L13A, FKBP12 is a human homolog of the yeast *FPR1* gene that encodes the most abundant FK506 rapamycin binding protein in *S. cerevisiae*, FRB is a small domain derived from full-length mTOR, Fig. 1*C*), resulting in efficient sequestration of the target away from the nucleus. In the absence of rapamycin, RPL13A-FKBP12 and the Rec8-FRB protein do not interact, allowing Rec8 to function normally. Upon rapamycin addition, a stable ternary complex forms between RPL13A-FKBP12 and the Rec8-FRB (Fig. 1*C*). This interaction occurs as the anchor protein (RPL13A) transits through the nucleus and associates with the ribosomes. Ribosomes incorporating RPL13A-FKBP12 are subsequently exported to the cytoplasm, carrying the bound Rec8-FRB along with them, leading to rapid and nearly complete cytoplasmic sequestration of the nuclear protein Rec8 (20). We hypothesized that using this method, we can only shunt off the dynamic pool of Rec8-cohesin outside the nucleus upon rapamycin addition, without removing the stable pool of Rec8-cohesin that topologically entraps sister chromatids (Fig. 1, *B* and *C*). We used the strains with genetic modifications essential for anchor-away (*RPL13A-2xFKBP12*, *fpr1*Δ, *tor1*Δ, with or without *ndt80*Δ for pachytene arrest) in the SK1 background (21), and C-terminally fused *REC8* with *FRB-GFP* using a nourseothricin selection marker in both the haploids and made homozygous diploid strains (GMY34 [*NDT80*] and GMY35 [*ndt80*Δ]).

To check the functionality of the anchor-away technique during meiosis, the homozygous diploid yeast strain (GMY35) was subjected to meiotic synchronization. We decided to add rapamycin at 4 h so that the pre-meiotic DNA replication is completed and Rec8-cohesin is loaded onto chromosomes for SCC by this time (22, 23, 24) (Fig. 1*D*). We confirmed the completion of DNA replication in 4 h using FACS analysis (Fig. S1*A*). The addition of rapamycin would only deplete the dynamic pool of Rec8-cohesin, but not the stable pool of Rec8-cohesin, because it is topologically tethered to sister chromatids. After adding rapamycin at 4 h in SPM, we collected samples for live cell imaging at different time points to quantify the kinetics of depletion of the dynamic pool of Rec8-cohesin. The fluorescence intensities from >100 nuclei were measured (after background subtraction) (Fig. 1*D* and S1*B*). This analysis showed that the fluorescence intensity drops by 75% (from 4506 ± 472.11 A.U. to 1138 ± 292.32 A.U., where ± refers to standard deviation) by 6 h (2 h after rapamycin addition). The remaining 25% Rec8-FRB-GFP remained inside the nucleus even after 10 h, representing a stable pool of Rec8-cohesin. This result suggests that the anchor-away technique succeeded in depleting the dynamic pool of Rec8-cohesin from the nucleus. The stable pool of cohesin remained in the nucleus as it could not be pulled away by the anchor-away due to its entrapment of the cohesin to chromosomes. Previous reports have shown fast depletion (within 3–30 min) of nuclear proteins upon rapamycin addition using anchor-away (20), whereas here it took 2 h to deplete only up to 75% of the Rec8-cohesin. This suggests that the dynamic pool of Rec8-cohesin was not available for immediate depletion, as it was chromatin-bound at the time of rapamycin addition. With time (within 2 h), it was evicted from the chromosomes and shunted to the cytoplasm by anchor-away. (Fig. 1, *B* and *C*). The remaining 25% of Rec8-cohesin, which failed to come out of the nucleus even after 10 h, represents the stable pool of cohesin. We also measured the fluorescence intensity for Rec8-FRB-GFP in the cytoplasm after DMSO and Rapamycin treatment. As expected, the cytoplasmic fluorescence was increased upon rapamycin treatment, suggesting the shunting off of the Rec8-FRB-GFP in the cytoplasm from the nucleus (Fig. S1*C*). To ensure that the rapamycin treatment did not alter the transcript and protein level for Rec8-FRB-GFP, we performed relative gene expression analysis using RT-qPCR and western blotting (using anti-Rec8 antibodies), respectively. We did not observe a significant change in the Rec8-FRB-GFP transcript level and protein levels (Fig. S1, *D* and *E*).

To quantify the mobile fraction for the dynamic and the stable pools of Rec8-FRB-GFP, we performed Fluorescence Recovery After Photobleaching (FRAP) analysis. Quantification of the FRAP curves (Figs. 1*E* and S2*A*) revealed 91 ± 0.2% mobile fraction of Rec8-FRB-GFP under DMSO treatment, which reduces to 24.6 ± 0.2% upon rapamycin treatment (where ± refers to standard deviation). These results indicate that the stable pool of cohesin (after rapamycin treatment) is mostly immobile, compared to the DMSO treatment.

To ensure that the anchor-away technique is efficient to deplete Rec8-cohesin completely from the nucleus if it is not chromatin-bound, we added rapamycin at 0 h in SPM, so that the newly synthesized Rec8 will immediately sequester in the cytoplasm before it gets loaded onto the sister chromatids. Under this condition, we observed complete loss of the Rec8-FRB-GFP nuclear fluorescence (Fig. S2*B*). This result suggests that the anchor-away technique is efficient to pull the Rec8-cohesin completely out of the nucleus if it is not topologically entrapping the sister chromatids. Hence, the partial depletion observed by adding the rapamycin at 4 h (Fig. 1*D*) represents the Rec8-cohesin that entraps the sister chromatids.

To further strengthen our argument that the anchor-away technique could deplete only the dynamic, but not the stable, pool of cohesin, we fused another cohesin subunit, Smc3, with FRB-GFP and performed rapamycin-induced depletion in mitotic culture. We hypothesized that the mitotically growing cells should show complete depletion of the nuclear fluorescence of Smc3-FRB-GFP (stable + dynamic pool) upon rapamycin addition once the cells pass through metaphase, because separase-induced cleavage of Scc1 opens the cohesin ring. However, if the cells are arrested at metaphase (using the microtubule depolymerizing drug benomyl), the chromatin-bound cohesin (stable pool) cannot be depleted from the nucleus upon rapamycin addition. We performed this experiment and observed the complete loss of Smc3-FRB-GFP signal from the nucleus (of >95% cells) within 90 min of rapamycin addition (Fig. S3). When we arrested cells at metaphase using benomyl, the Smc3-FRB-GFP signal reduced in >95% cells as the dynamic pool of Smc3-FRB-GFP was shunted to the cytoplasm due to rapamycin addition; however, >95% cells showed the nuclear fluorescence of Smc3-FRB-GFP (stable pool) even after 90 min of rapamycin induction (Fig. S3). This result confirms that the anchor-away method is efficient for complete depletion of cohesin subunits if they are not chromatin-bound (topologically entrapping the sister chromatids).

### Sister-chromatid cohesion and sister kinetochore biorientation were not perturbed after depleting the dynamic pool of Rec8-cohesin

To ensure that we depleted only the dynamic pool of Rec8-cohesin while keeping the stable pool of Rec8-cohesin intact, we performed a sister chromatid segregation assay to check for SCC defects and sister kinetochore monoorientation defects. We hypothesized that if the stable pool of cohesin is enough to maintain SCC, rapamycin addition should not compromise SCC. To address this, we integrated heterozygous *CENV-GFP* (using the TetO/TetR-GFP system) in GMY34, performed meiotic synchronization, added rapamycin after 3 h in SPM and performed an immunofluorescence-based SCC and sister kinetochore monoorientation assay as described previously (25).

**Figure 2:**
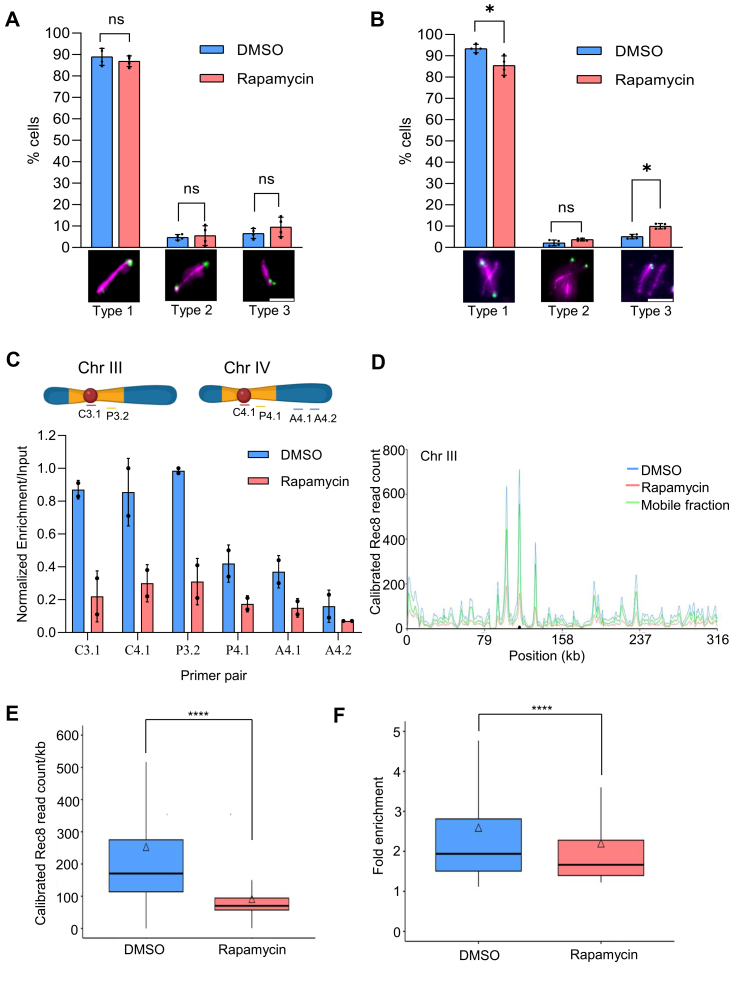
**Sister**-**chromatid cohesion assay and quantification of cohesin binding with chromatin.***A*, immunofluorescence using anti-tubulin antibodies (*Red*) and DAPI staining (*blue*) was performed to quantify the segregation of sister chromatids (labelled using heterozygous *CENV-GFP, Green*) during anaphase I. n > 300. Type 1 represents faithful segregation of sister chromatids during meiosis I, Type 2 represents segregation of sister chromatids during meiosis I due to combined defect in sister kinetochore monoorientation and sister-chromatid cohesion, and Type 3 represents separation of sister chromatids due to weak cohesion. The experiment was repeated four times, and the error bars represent SD. ns: non-significant, by Mann-Whitney U test. Individual data points are overlaid on the bar graph, with each point representing a biological replicate. Scale: 5 μm. *B*, Immunofluorescence using anti-tubulin antibodies (*Red*) and DAPI staining (*blue*) was performed to quantify the segregation of sister chromatids (labelled using heterozygous *CENV-GFP, Green*) during anaphase II. n > 300. Type 1 represents faithful segregation of sister chromatids during meiosis I and II, Type II represents meiosis I disjunction of sister chromatids due to combined defects of the sister kinetochore monoorientation and the protection of centromeric cohesion, Type III represents non-disjunction of sister chromatids during meiosis II, due to weak cohesion between sisters. The experiment was repeated four times, and the error bars represent SD. ns: non-significant, ∗*p* < 0.05 by Mann-Whitney U test. Individual data points are overlaid on the bar graph, with each point representing a biological replicate. Scale: 5 μm. *C*, ChIP-qPCR to quantify the binding of Rec8-cohesin at centromeres (*red*), pericentromeres (*yellow*), and arm regions (*blue*) of chromosomes III and IV. *Top panel*: The schematics of chromosomes III and IV represent the tentative position of the primer pairs (not to scale) used for qPCR analysis. C3.1 represents the centromere of chromosome III, C4.1 represents the centromere of chromosome IV, P3.2 represents the pericentromere of chromosome III, P4.1 represents the pericentromere of chromosome IV, A4.1 represents the CAR site on chromosome IV, and A4.2 represents the non-CAR site on chromosome IV. Appropriate ‘No antibody’ control was kept during the assay, but the data is not presented here because of its values close to zero. The experiment was repeated twice, and the error bars represent SD. Individual data points are overlaid on the bar graph, with each point representing a biological replicate. *D*, calibrated ChIP-seq analysis to quantify the binding of Rec8-cohesin throughout the genome (all 16 chromosomes of *S. cerevisiae*). The experiment was performed the same way as for Figure 2*C* and repeated twice. A representative plot of the Rec8 binding profile on chromosome III is shown here. The *black dot* indicates the centromere, and the chromosomal coordinates are mentioned on the x-axis. The mobile fraction was calculated by subtracting the stable pool (Rapamycin-treated sample) from the dynamic + stable pool (DMSO-treated sample). The Rec8 binding profiles on other chromosomes are shown in Fig. S2. *E*, Boxplot representing Rec8 read density in rapamycin-treated and DMSO-treated conditions (∗∗∗∗*p*< 0.0001, Wilcoxon rank sum test). The triangle indicates the mean value. *F*, comparison of Rec8 fold enrichment with respect to input in rapamycin-treated and DMSO-treated conditions (∗∗∗∗*p*< 0.0001, Wilcoxon rank sum test). The triangle indicates the mean value.

We selectively scored cells with anaphase I spindle and observed the segregation of the GFP spots in a wild-type background (Fig. 2*A*). During anaphase I, the sister chromatids remain associated and can be seen as a single GFP foci within one of the two nuclei (Type 1, Fig. 2*A*). Neither SCC nor sister kinetochore monoorientation defect alone can segregate sister chromatids to two nuclei during anaphase I. Both defects simultaneously can lead to the segregation/disjunction of sister chromatids to different nuclei during anaphase I (Type 2, Fig. 2*A*). Only a SCC defect can lead to the appearance of two separated green foci in a single nucleus during anaphase I (Type 3, Fig. 2*A*). We observed faithful segregation of the sister chromatids in >90% of cells in the control condition (DMSO, Fig. 2*A*). We could not see any significant decrease in the faithful segregation of sister chromatids after adding rapamycin at 3 h (Fig. 2*A*). This result suggests that the SCC and sister kinetochore monoorientation remain unperturbed even after depleting the dynamic pool of Rec8-cohesin, suggesting that the stable pool of Rec8-cohesin is enough to maintain SCC. This result is consistent with the previous report, which showed that only 13% of Mcd1-cohesin is enough to maintain SCC (26).

To further quantify the sister chromatid missegregation during meiosis II, we selectively scored the meiotic cells with anaphase II spindles and categorized them into three types based on the position of sister *CEV-GFP* with respect to the spindles (Fig. 2*B*): Type 1: separated GFP spots with the same spindle, Type 2: separated GFP spots with different spindles, Type 3: twin GFP spots at one end of spindles (sister chromatid nondisjunction). Type 1 represents faithful chromosome segregation throughout meiosis, type 2 represents MI disjunction of sister chromatids due to combined defects of the sister kinetochore monoorientation and the protection of centromeric cohesion, and type 3 represents sister chromatid nondisjunction in meiosis II due to a defect only in centromeric SCC. We observed a modest increase (∼20%) in sister chromatid missegregation (type 3, Fig. 2*B*) after rapamycin treatment. However, 80% of cells faithfully segregated the sister chromatids, suggesting that depleting the dynamic pool of Rec8-cohesins does not affect the SCC and sister kinetochore monoorientation functions in most of the cells. A little increase (∼20%) in the sister chromatid missegregation may be because a few cells (∼20%) entered meiosis late and completed DNA replication by 4 to 5 h (instead of 3–4 h), and the cohesin was depleted before the completion of cohesin loading in those cells. Alternatively, the defects in prophase I (shown below) could induce the segregation errors shown here. It is important to note that a previous report has shown the sister chromatid missegregation to ∼80% in the *rec8*Δ mutant (27), whereas, in our study, we could see this defect in only 20% of cells, suggesting that the SCC is not significantly perturbed due to the loss of the dynamic pool of Rec8-cohesin.

### ChIP-qPCR and calibrated ChIP-seq analysis showed a reduction of the dynamic pool of Rec8-cohesin on chromosomes

To quantify the reduction in the chromatin-bound Rec8-cohesin upon rapamycin treatment, we performed ChIP-qPCR and calibrated ChIP-seq. We designed primers from different regions of chromosomes III and IV (centromeres, pericentromeres, and CAR [Cohesin Associated Regions] site and non-CAR sites on chromosome arms, Fig. 2*C*) to quantify the association of Rec8-cohesin with chromatin at these regions by RT-qPCR (28, 29). The meiotic synchronization was carried out for the *ndt80*Δ strain (GMY35) as used in Figure 1*D*. Rapamycin was added at 4 h in SPM, and the samples were fixed for the ChIP-qPCR and calibrated ChIP-seq analysis at 6 h Rec8 was pulled down using an anti-mTOR antibody. Our ChIP-qPCR analysis revealed a significant reduction in the chromatin-bound Rec8-cohesin after rapamycin treatment at all the locations tested (Fig. 2*C*). To further confirm this result and to see if the reduction in Rec8-cohesin association with chromatin (upon rapamycin treatment) is throughout the chromosomes or at some specific locations (CAR sites, centromeres, and pericentromeres), we performed a calibrated ChIP-seq analysis. The Rec8 ChIP-seq profile showed that the reduction is throughout the chromosomes, without any preference for any specific location (Figs. 2*D* and S4). Quantification of the genome-wide Rec8 binding in Rapamycin-treated and DMSO-treated samples also showed reduced Rec8 read density due to Rapamycin treatment (Fig. 2*E*). The mean (87.7) and median (70.1) Rec8 read density in Rapamycin-treated samples were significantly lower than the mean (248.9) and median (170.4) Rec8 read density in the DMSO-treated samples. Although, the total number of Rec8 peaks in the Rapamycin-treated samples (968) were similar to the total number of Rec8 peaks (1041) in the DMSO-treated samples (Table S1), the Rec8 fold enrichment relative to the input was significantly lower in the Rapamycin-treated samples (mean: 2.2, median: 1.7) compared to the DMSO-treated samples (mean: 2.6, median: 1.9) (Fig. 2*F*). Collectively, these results suggest that the rapamycin treatment indeed results in the reduction of the overall chromatin-bound Rec8-cohesin, irrespective of any specific locations. The observed reduction is due to the removal of the dynamic pool of Rec8-cohesin; however, the stable pool of Rec8-cohesin remains chromatin-bound to offer SCC.

### Depletion of the dynamic pool of Rec8-cohesin delays meiosis, reduces sporulation efficiency, and affects spore viability

**Figure 3:**
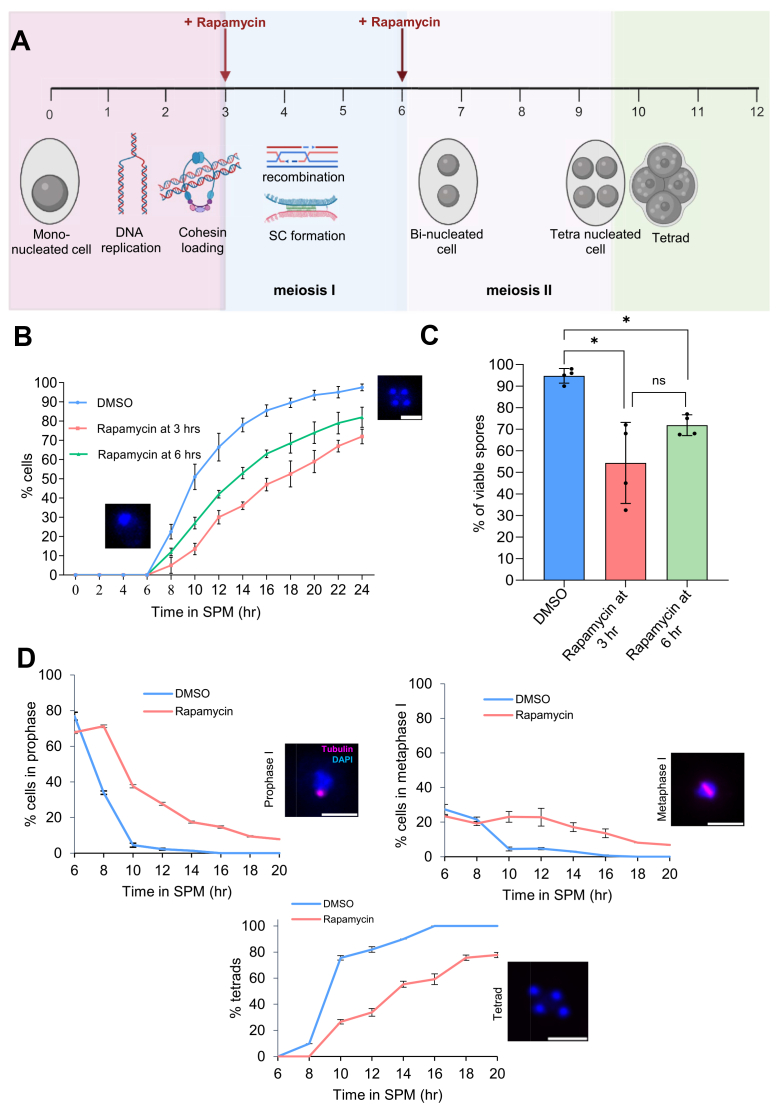
**Depletion of the dynamic pool of Rec8-cohesin delays meiosis and reduces spore viability.***A*, timeline for meiotic synchronization and rapamycin addition. During the first 3 to 4 h in SPM, meiotic DNA replication and cohesin loading take place. Meiotic recombination and homolog pairing (SC assembly) take place between 4–7 h in SPM. We decided to add rapamycin at two different time points: 1) at 3 h in SPM (for depleting the dynamic pool of Rec8-cohesin before meiotic recombination, but after DNA replication and cohesin loading), and at 6 h in SPM (for depleting the dynamic pool of Rec8-cohesin before chromosome segregation, but after meiotic recombination). *B*, meiotic progression analysis in the presence and absence of rapamycin. DAPI-stained nuclei were counted from SPM until 24 h, in the presence and absence of rapamycin. The experiment was repeated three times, and the error bars represent SD. n > 200. Scale: 5 μm. *C*, tetrads obtained by meiotic synchronization were dissected using a micromanipulator on YPD plates. The percentage of spore viability (number of germinated spores divided by the total number of spores, x 100) was calculated by dissecting 100 tetrads. Plates were incubated at 30 °C for 72 h for spore germination. The experiment was repeated four times, and the error bar represents the SD. Statistical significance was assessed using two-tailed Mann–Whitney U tests. ns: not significant, *∗p* < 0.05. Individual data points are overlaid on the bar graph, with each point representing a biological replicate. *D*, immunofluorescence using anti-tubulin antibodies and DAPI to visualize the relative positions of spindles and nuclei to identify the stages of meiosis. The experiment was repeated twice. More than 100 cells were counted for each time point. The error bar represents the SD. Scale: 5 μm.

To observe the defect in meiosis after depleting the dynamic pool of Rec8-cohesin, the homozygous diploid yeast strain (GMY34) was subjected to meiotic synchronization. The rapamycin was added at either 3 h or 6 h in SPM to deplete the dynamic pool of Rec8-cohesin after cohesin loading but before meiotic recombination, and after meiotic recombination but before chromosome segregation, respectively (Fig. 3*A*). The percentage of tetrads/tetra-nucleated cells, the end product of meiosis, was counted every 2 h, until 24 h, to compare the rate of meiotic progression. We observed that the addition of rapamycin at 3 h and 6 h delayed the rate of meiotic progression compared to the control (DMSO) (Fig. 3*B*). The addition of rapamycin at 3 h severely affected meiotic progression, compared with the addition of rapamycin at 6 h. Also, the sporulation efficiency was significantly reduced upon rapamycin addition at 3 h (∼75%) and 6 h (∼80%), compared to the control (97%) (Fig. 3*B*). Next, we dissected at least 100 tetrads from each of these samples to score the spore viability. We observed that the spore viability reduces drastically upon the addition of rapamycin at 3 h and 6 h (54.4% and 72.2%, respectively) compared to the control (92.5%, Fig. 3*C*). As the depletion of the dynamic pool of Rec8-cohesin delays meiosis, we wanted to know at which stage of meiosis cells take a long time to pass through or arrest. We performed immunofluorescence using anti-tubulin antibodies and DAPI staining to quantify the number of cells at different stages of meiosis. Rapamycin was added at 3 h in SPM, and samples were harvested every 2 h, from 6 h to 20 h. We observed that a significant population of cells delays/arrests at prophase I and metaphase I (Fig. 3*D*), but not at any other stages of meiosis (Fig. S5). This result suggests that the dynamic pool of Rec8-cohesin is required during prophase I and metaphase I to maintain the pace of meiotic progression. All these results collectively suggest important functions of the dynamic pool of Rec8-cohesin during meiosis I.

### The absence of the dynamic pool of Rec8-cohesin shows defects in meiotic DSB formation, crossover formation, and synaptonemal complex assembly

**Figure 4:**
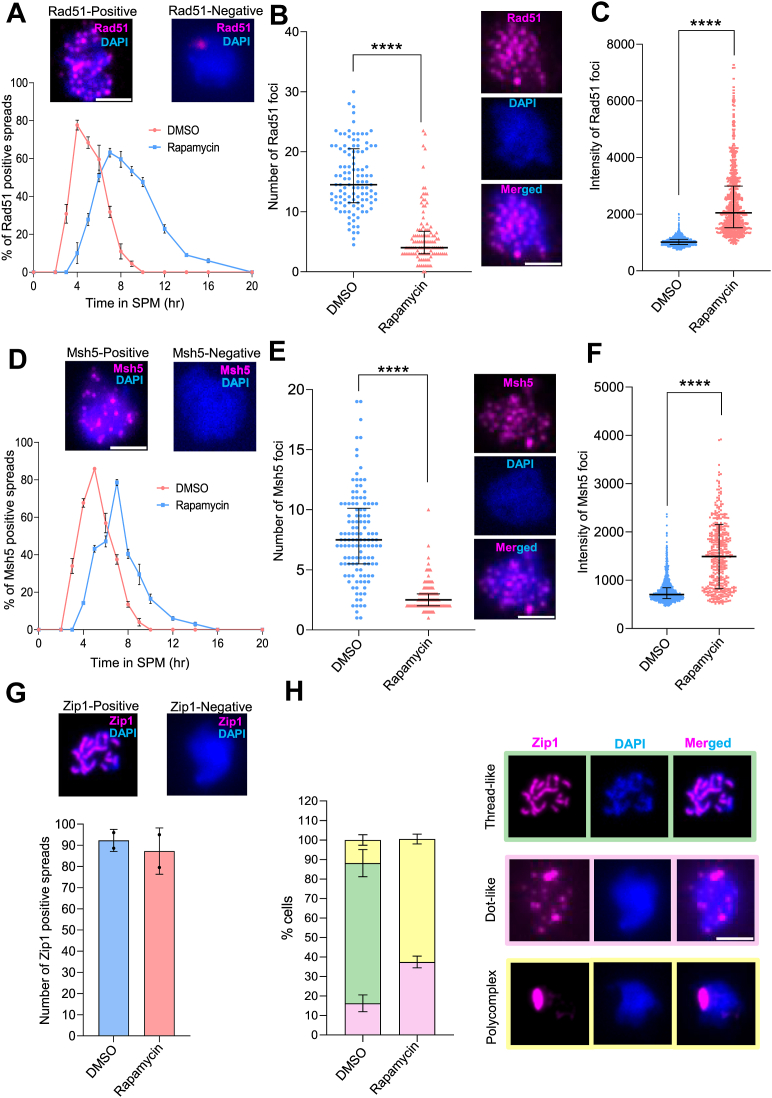
**Chromatin spread analysis to quantify defects in DSB formation, crossover frequency, and SC assembly upon depleting the dynamic pool of Rec8-cohesin.***A*, chromatin spread using anti-Rad51 antibodies and DAPI staining. Representative images show Rad51-positive and Rad51-negative cells. Spreads showing more than 2 foci are considered as Rad51-positive spreads. n > 100 spreads for each condition. The experiment was repeated twice, and the error bars represent SD. Scale: 5 μm. *B*, the dot plot represents the number of Rad51 foci per spread at the peak time observed in Figure 4*A* (*i.e.*, 4 h for the DMSO-treated sample and 7 h for the rapamycin-treated sample). For each condition, >100 spreads were scored per experiment, and two independent biological replicates were analysed. Each dot represents the number of Rad51 foci in a single spread. Horizontal lines indicate the median, and error bars denote the interquartile range. Statistical significance was assessed using a two-tailed Mann–Whitney U test. ∗∗∗∗*p* < 0.0001. Scale: 5 μm. *C*, fluorescence intensity of each spot of Rad51 was quantified from chromatin spreads prepared from DMSO- or rapamycin-treated cells. For each condition, >100 spreads were analysed per experiment, and two independent biological replicates were examined. Each dot represents the integrated density of Rad51 foci. Horizontal lines indicate the median, and error bars denote the interquartile range. Statistical significance was assessed using a two-tailed Mann–Whitney U test. ∗∗∗∗*p* < 0.0001. *D*, chromatin spread using anti-Msh5 antibodies and DAPI staining. Representative images show Msh5-positive and Msh5-negative cells. n > 100 spreads for each condition. The experiment was repeated twice, and the error bars represent SD. Scale: 5 μm. *E*, the dot plot represents the number of Msh5 foci per spread at the peak time observed in Figure 4*D* (*i.e.*, 5 h for the DMSO-treated sample and 7 h for the rapamycin-treated sample). For each condition, >100 spreads were scored per experiment, and two independent biological replicates were analysed. Each dot represents the number of Msh5 foci in a single spread. Horizontal lines indicate the median, and error bars denote the interquartile range. Statistical significance was assessed using a two-tailed Mann–Whitney *U* test. ∗∗∗∗*p* < 0.0001. Scale: 5 μm. *F*, Fluorescence intensity of each spot of Msh5 was quantified from chromatin spreads prepared from DMSO- or rapamycin-treated cells. For each condition, >100 spreads were analysed per experiment, and two independent biological replicates were examined. Each dot represents the integrated density of Msh5 foci. Horizontal lines indicate the median, and error bars denote the interquartile range. Statistical significance was assessed using a two-tailed Mann–Whitney *U* test. ∗∗∗∗*p* < 0.0001. *G*, chromatin spread using anti-Zip1 antibodies and DAPI staining. Representative images show Zip1-positive and Zip1-negative cells. n > 100 spreads for each condition. The experiment was repeated twice, and the error bars represent SD. Statistical significance was assessed using a two-tailed Mann–Whitney *U* test. ns: non-significant. Individual data points are overlaid on the bar graph, with each point representing a biological replicate. Scale: 5 μm. *H*, based on the Zip1 localization pattern, the spreads are categorized into three categories: chromosomal localization of Zip1 only (thread-like), reduced chromosomal localization of Zip1 (dot-like), and absence of Zip1 from chromosomes (polycomplex). n > 100 spreads for each condition. The experiment was repeated twice, and the error bars represent SD. Scale: 5 μm.

Meiotic recombination is a multi-step process that takes place during prophase I of meiosis. During this process, several steps are involved, such as DNA double-strand Break (DSB) formation and crossover formation (30). The recombination occurs concomitantly with the assembly and disassembly of the Synaptonemal Complex (SC), with an intimate relationship between the two events (30). To understand which process of meiotic recombination is perturbed in the absence of the dynamic pool of Rec8-cohesin, we performed a series of immuno-staining analyses of chromosome spreads using anti-Rad51, anti-Msh5, and anti-Zip1 antibodies. Rad51 localizes to the DSB breaks (30, 31, 32, 33, 34); hence, it helps in quantifying the number of DSBs per cell and/or their turnover. Msh5 facilitates crossover formation (30, 35); hence, it helps in quantifying crossover efficiency. Zip1 is a protein in the central regions of SC, which indicates the efficiency of chromosome synapsis (30, 36). For these experiments, we added rapamycin at 3 h in SPM to deplete the dynamic pool of Rec8-cohesin when meiotic recombination starts. For Rad51 staining, first, we counted the number of Rad51-positive spreads at different time points (Fig. 4*A*). We observed that there is a delay of ∼3 h in the peak of Rad51-positive cells in the rapamycin-added condition compared to the DMSO-treated cells. The sample treated with DMSO shows a peak of the Rad51-positive spread at 4 h, compared to the rapamycin-treated sample that shows the peak of Rad51-positive spreads at 7 h (Fig. 4*A*). So, we counted the number of Rad51-foci per cell at the respective time points (4 h for DMSO-treated and 7 h for rapamycin-treated samples, Fig. 4*B*). We observed a significant reduction in the number of Rad51 foci in the rapamycin-treated cells compared to the DMSO-treated cells (15.6 ± 5.3–5.8 ± 2.3, *p* < 0.0001, where ± refers to standard deviation, Fig. 4*B*), suggesting a reduction in the DSB formation (or rapid turnover). To ensure that the reduction in the number of Rad51-foci per spread is not due to different time points (4 h *versus* 7 h), we counted the same for several other time points (Fig. S6*A*). We found a reduction in the number of Rad51 foci per spread throughout all the time points upon rapamycin treatment. This result suggests the role of the dynamic pool of Rec8-cohesin in DSB formation. This is consistent with the reduced DSB formation in the *rec8Δ* mutants (37, 38). Interestingly, we observed that the fluorescence intensity of Rad51 foci in rapamycin-treated conditions is significantly higher compared to the DMSO-treated cells (1032 ± 150–2408 ± 1175, *p* < 0.0001, where ± refers to standard deviation, Figures 4*C* and S6*B*). Also, the disappearance of Rad51 foci in the presence of rapamycin was delayed (Fig. 4*A*). These results suggest that the accumulation of Rad51 at the DSB sites and the dynamic pool of cohesin may be required for the timely dissociation of Rad51 from the DSB sites.

Similarly, we performed the chromatin spread with anti-Msh5 antibodies to count the number of foci per cell, which represents the crossover formation. The DMSO-treated cells showed the peak of the Msh5-positive cells at 5 h, whereas the rapamycin-treated cells showed the peak of the Msh5-positive cells at 7 h, suggesting a 2-h delay in crossover formation (Fig. 4*D*). Depletion of the dynamic pool of Rec8-cohesin reduces the number of Msh5 foci significantly compared to the DMSO-treated sample (7.7 ± 3.6–2.7 ± 1.2, *p* < 0.0001, where ± refers to standard deviation, Fig. 4*E*). To ensure that the reduction in the number of Msh5-foci per spread is not due to different time points (5 h *versus* 7 h), we counted the same for several other time points (Fig. S6*C*). We found a reduction in the number of Msh5 foci per spread throughout all the time points upon rapamycin treatment. These results suggest the role of the dynamic pool of Rec8-cohesin in crossover formation. However, given the reduction of Rad51-focus formation (Fig. 4, *A*–*C*), it is likely that the reduced Msh5 foci could be explained by the reduction of DSB formation and delayed DSB repair since the Msh4-5 complex works near Dmc1-Rad51 recombinases (35). Also, we observed that the fluorescence intensity of Msh5 foci in rapamycin-treated conditions is significantly higher compared to the DMSO-treated cells (771 ± 239–1758 ± 693, *p* < 0.0001, where ± refers to standard deviation, Figs. 4*F* and S6*D*), suggesting the accumulation of Msh5 and the dynamic pool of Rec8-cohesin is responsible for the timely dissociation of Msh5.

To understand the role of the dynamic pool of Rec8-cohesin in SC assembly and/or chromosome synapsis, we performed chromatin spread using anti-Zip1 antibodies from pachytene-arrested cells (strain: GMY35). We observed that the number of Zip1-positive spreads is almost the same (>85%) at 6 h (for DMSO-treated sample) and 8 h (for rapamycin-treated sample) (Fig. 4*G*). We categorized the Zip1 staining into three types, as shown in Figure 4*H*. The faithful assembly of the SC should show thread-like structures of Zip1, whereas any defect in SC assembly leads to dot-like appearance and polycomplexes. The polycomplex represents the aggregated mass of Zip1 due to failure in SC assembly. We observed that the DMSO-treated sample showed thread-like structures of Zip1 in 73% spreads, whereas the rapamycin-treated sample showed polycomplexes in 60% spreads and a dotted appearance in 40% spreads (Fig. 4*H*). Given that the depletion of the Rec8-cohesin showed defects in DSB formation and repair, the abnormal assembly of Zip1 could be due to the defective meiotic recombination. Or else, the defective SC formation caused the abnormal recombination.

Overall, all these results together suggest the role of the dynamic pool of Rec8-cohesin in multiple processes of meiotic recombination.

### A dynamic pool of Rec8-cohesin is not required for SPB cohesion and duplication

**Figure 5:**
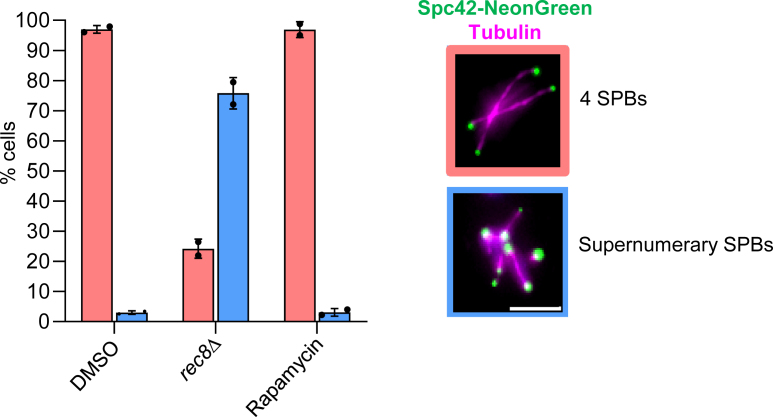
**The dynamic pool of Rec8-cohesin is not required for SPB cohesion and duplication.** Immunofluorescence analysis using anti-Tub1 antibodies and DAPI staining to identify cells in the anaphase II stage. The number of cells with 4 SPBs (Spc42-NeonGreen) or more were counted. n > 100. The experiment was repeated two times, and the error bars represent SD. Individual data points are overlaid on the bar graph, with each point representing a biological replicate. Scale: 5 μm.

Previously, Rec8-cohesin has been implicated in maintaining the SPB cohesion and duplication (19), as its absence shows supernumerary SPBs after meiosis II. So, we asked the question of whether the stable pool or the dynamic pool of Rec8-cohesin is required for this process. To address this question, we visualized the SPBs by endogenous *SPC42-NeonGreen* tagging and depleted the dynamic pool of Rec8-cohesin using anchor away by adding rapamycin at 3 h in SPM. We observed the SPBs at the end of meiosis by live cell imaging of tetrads. The *rec8*Δ strain was used as a positive control for SPB cohesion defect. We could not observe supernumerary SPBs in rapamycin-treated samples, whereas the *rec8*Δ mutant showed supernumerary SPBs in >75% cells (Fig. 5). This result suggests that the dynamic pool of Rec8-cohesin is not required for SPB cohesion and duplication. The stable pool of Rec8-cohesin, which also provides SCC, is enough for SPB cohesion and duplication.

### The dynamic pool of Rec8-cohesin regulates the transcription level of key meiotic regulators

Cohesin has been implicated in regulating gene expression during mitosis (18) and meiosis (39, 40). However, whether the stable pool or the dynamic pool is required to regulate the expression of the key meiotic regulators is not investigated.

**Figure 6:**
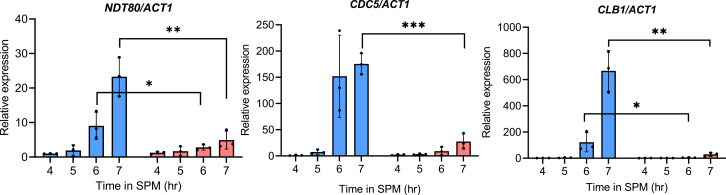
**Relative gene expression analysis using RT-qPCR for key meiotic genes.** The expression of the housekeeping gene *ACT1* was used for normalization and represents the fold change in expression of the DMSO and rapamycin added condition. The experiment was repeated thrice. Error bars represent SD. ∗*p* < 0.05, ∗∗*p* < 0.01, ∗∗∗*p* < 0.001, by two-tailed unpaired *t* test with Welch’s correction. Only significant *p*-values have been shown. Individual data points are overlaid on the bar graph, with each point representing a biological replicate.

To quantify the effect of depleting the dynamic pool of Rec8-cohesin on meiotic transcription, we performed relative gene expression analysis by mRNA extraction and RT-qPCR. As we observed a delay in meiotic progression during prophase I and metaphase I, we restricted our analysis to only a few early meiotic genes (*IME1*, *IME2*, *UME6*, *HOP1*, *RED1*, *SPO11*, *MRE11*, *ZIP1*) and a few middle meiotic genes (*NDT80*, *CLB1*, *SPO13*, *CDC5*, *REC8*, *MSH5*). The rapamycin was added into SPM at 3 h, and the samples were harvested for the mRNA extraction at later time points from the DMSO-treated and rapamycin-treated samples. This analysis revealed that the depletion of the dynamic pool of Rec8-cohesin alters the transcription of a few middle meiotic genes (*NDT80*, *CDC5*, and *CLB1*) only, whereas the expression of the early meiotic genes (*IME1*, *IME2*, *UME6*, *HOP1*, *RED1*, *SPO11*, *MRE11*, *ZIP1*) and a few middle meiotic genes (*SPO13*, *REC8*, *MSH5*) was not perturbed significantly (Figs. 6 and S7). This is quite expected because the addition of rapamycin at 3 h will deplete the dynamic pool of Rec8-cohesin by 5 h by the time early meiotic genes must have been expressed. Ndt80 is a meiosis-specific transcription factor that plays a crucial role in activating the middle sporulating genes (including B-type cyclin *CLB1*) necessary for meiotic divisions and spore formation (41). Cdc5, a polo-like kinase, plays a crucial role in exit from pachytene by resolving the Holliday junction and disassembling the SC (42). This result suggests that the dynamic pool of Rec8-cohesin is essential for maintaining the meiotic transcription program and the pace of meiotic progression. The delay observed in the meiotic progression (Fig. 3, *B* and *D*) may be due to the delay in the transcription of the key meiotic genes.

## Discussion

Cohesin has emerged as a multifunctional complex with roles extending beyond its canonical function in SCC, including loop extrusion, genome organization, and transcriptional regulation (18). These diverse functions are closely linked to the dynamic behavior of cohesin on chromatin, its subunit composition, stoichiometric changes and regulatory post-translational modifications (4, 7, 8, 9). The SCC function needs a stable association of cohesin with sister chromatids to keep them together from pre-meiotic S-phase to anaphase II, whereas meiotic chromosome morphogenesis, recombination, genome organization and transcription regulation through loop extrusion need a dynamic association of cohesin with chromatin. Hence, it is unclear whether two different pools of cohesin (stable and dynamic) exist during yeast meiosis.

Our study provides direct evidence that Rec8-cohesin in *S. cerevisiae* forms two distinct chromatin-bound pools with separable functions: a dynamic pool required for meiotic recombination, SC formation, and regulation of meiotic transcription, and a stable pool required to maintain SCC and SPB cohesion. Using an anchor-away system to selectively deplete the dynamic pool of Rec8, we targeted cohesin complexes with high chromatin turnover while preserving the stably bound fraction. Upon rapamycin treatment, we observed ∼75% depletion of nuclear Rec8-FRB-GFP, consistent with loss of a dynamic chromatin-associated pool, while ∼25% of Rec8 remained nuclear even after 10 h, representing a stably bound fraction. The phenotypic consequences of this depletion—reduced sporulation efficiency, recombination defects, failure to assemble a functional SC, and transcription reduction/delays, but intact SCC, SPB cohesion and kinetochore monoorientation—demonstrate functional specialization of these pools. The dynamic pool may facilitate chromosome morphogenesis required for homolog recombination, SC assembly and promoter-enhancer contacts through loop extrusion for transcription regulation. A future experiment using a Chromosome-Conformation Capture method (Hi-C) may provide detailed insight into perturbations in loop extrusion in the absence of the dynamic pool of Rec8-cohesin. The stable pool topologically entraps sister chromatids for SCC and potentially a few SPB components to facilitate SPB cohesion. Separation-of-function alleles of *REC8* (10, 43) may provide tools to dissect the molecular determinants of these pools. Also, some cohesin binding proteins, or the post-translational modifications, or the stoichiometry of cohesin subunits may be responsible for differentiating Rec8-cohesin into two different pools, which needs further investigation.

Our findings raise mechanistic questions regarding how a single Rec8-based cohesin complex can adopt both stable and dynamic chromatin interactions. In *Drosophila*, functionally distinct meiotic cohesin populations have been identified: a dynamic pool containing the kleisin subunit C(2)M, which is essential for interhomolog recombination and synaptonemal complex (SC) assembly, and a stable pool composed of SOLO, SUNN, and ORD, which is required for SCC (7). So, the functional specificity is encoded at the subunit level. In contrast, yeast relies on a single kleisin (Rec8), suggesting that regulation must occur at the level of post-translational modification, interaction with accessory proteins, stoichiometry of the cohesion subunits or chromatin context. One attractive hypothesis is that Eco1/Ctf7-mediated acetylation of Smc3, known to stabilize cohesin on chromatin during SCC establishment (44), may also distinguish stable from dynamic pools. If unacetylated Smc3 promotes dynamic association, and acetylation commits the complex to stable entrapment, this could provide a molecular mechanism to switch cohesin function. Future studies will be needed to test whether the dynamic pool is devoid of Smc3 acetylation, and whether manipulating acetylation dynamics affects Rec8-cohesin pool identity.

Additionally, our anchor-away approach failed to pull the stable pool of cohesin out of the nucleus upon rapamycin addition. This result allows us to speculate that the opening and closing of the cohesin entry and exit gates (Smc3-Smc1 interface/Smc3-Rec8 interface) may not be required for the dynamic association of cohesin with chromatin, whereas the stable pool of cohesin requires the opening/closing of the entry/exit gates for entrapment and release of the sister chromatids. Further experiments using mutants of Scc2-Scc4 (facilitate opening of the entry gate) and Rad61 (facilitates opening of the exit gate) may address this conundrum.

Moreover, the possibility of cohesin dimerization further complicates this picture. A recent study in human cells using 3D structured illumination microscopy identified both monomeric and dimeric forms of cohesin (9). They found that while most chromatin-bound cohesin is dimeric, the monomeric rings are responsible for SCC, suggesting that dimeric form may represent a dynamic pool. Whether similar dimeric complexes exist in yeast, and whether they correspond to the dynamic pool described here, is an intriguing possibility that deserves further investigation.

Our data also highlight kinetic differences in the depletion of nuclear Rec8 by anchor-away. The ∼2-h timeframe for dynamic Rec8 depletion, much slower than typical nuclear proteins targeted by this system (usually within 3–30 min) (20), suggests a degree of chromatin retention or structural entrapment that impedes rapid nuclear export. This observation supports the notion that even the dynamic pool is chromatin-associated but differs in binding mode or residence time compared to the stably entrapped pool.

To resolve these outstanding questions, future work must move beyond population-level measurements. Single-cell and single-molecule approaches may be adopted to quantify the heterogeneity in the cell population and cohesin stoichiometry, dynamics, and modification state *in vivo* (4, 8, 9). In summary, our study demonstrates that *S. cerevisiae* Rec8-cohesin is functionally partitioned into dynamic and stable chromatin-bound populations, enabling the same complex to mediate both dynamic chromosome remodeling and stable chromatid cohesion. These findings establish a conceptual framework in which cohesin function is not solely dictated by its subunit composition but also by its chromatin binding dynamics—principles likely conserved across eukaryotes.

## Experimental procedures

### Yeast strains

All the yeast strains constructed for this study are isogenic to the SK1 background (Table S2). For gene deletion and C-terminal fusions of proteins, a PCR-based homologous recombination approach was used as described elsewhere (45, 46) using appropriate plasmid-borne cassettes obtained from Euroscarf/Addgene. The primer sequences are depicted in Table S3. All the C-terminal fusions were confirmed by diagnostic PCR or western blotting, or fluorescence imaging. Homozygous diploids were made using a micromanipulator and verified by crossing with tester yeast strains. Parental yeast strains for the anchor-away technique were gifted by Viji Subramanian (IISER) and Andreas Hochwagen (New York University), as reported previously (21). Plasmid for FRB-GFP tagging was obtained from Susan Forsburg’s lab (University of Southern California), as reported previously (47). All the plasmids used in this study are listed in Table S4.

### Meiotic synchronization

Cells were patched on a YPG plate (1% (w/v) yeast extract, 2% (w/v) peptone, 3% (w/v) glycerol, 2% (w/v) agar) from the glycerol stock and incubated for 15 h. From the YPG plate, cells were streaked for isolation of a single colony on the YPD plate (1% (w/v) yeast extract, 2% (w/v) peptone, 2% (w/v) dextrose, 2% (w/v) agar), and kept for 48 h. A single colony was inoculated in 5 ml of YPD broth and kept shaking for 24 h. Cells from the YPD-grown culture were diluted in synthetic pre-sporulation (SPS) media (0.5% (w/v) yeast extract, 2% (w/v) peptone, 1% potassium acetate, 0.17% (w/v) nitrogen base without ammonium sulfate and amino acid, 0.5% (w/v) Ammonium sulphate, 0.05 M potassium hydrogen phthalate; 50 ml media in 500 ml flask) at O.D._600_ 0.2. Cells were grown in SPS media for 15 to 18 h until O.D._600_ reached 1.2 to 1.8. Cells were washed twice with prewarmed sporulation media (SPM; 0.3% (w/v) potassium acetate, 0.02% (w/v) raffinose) and resuspended in 50 ml of SPM in a 500 ml flask at O.D._600_ 1.2 to induce sporulation. Samples were harvested from SPM at indicated time points. Throughout the meiotic synchronization, the temperature was maintained at 30 °C, and shaking was performed at 230 RPM.

For rapamycin treatment, 1 μg/ml rapamycin (Gold Bio, Cat# R-1010-25) was added (from 1 mg/ml stock in DMSO (PureSynth, Cat# PSR38154) at 4 h in SPM (for *ndt80*Δ strain) and at 3 h in SPM (for *NDT80* strain). For control experiments, an equal volume of DMSO was added.

### DAPI staining for sporulation efficiency

Sporulation efficiency was determined by staining the nuclei with DAPI. Briefly, 1 ml sample from *S. cerevisiae* meiotic cultures was harvested in a 1.5 ml microcentrifuge tube, centrifugation was done at 1690*g* for 1 min, and the supernatant was discarded. Cells were fixed by adding 1 ml of 70% ethanol and kept at room temperature for 2 h. Fixed cells were washed with 1X PBS twice and resuspended in PBS containing DAPI at a final concentration of 1 μg/ml. After a 10-min incubation in the dark, cells were mounted on microscope slides and examined by fluorescence microscopy with 63X oil immersion objective lens. At least 200 cells were counted at each time point. Cells containing two, three, or four distinct DAPI-stained nuclei were counted as tetrads. Sporulation efficiency was calculated by dividing the number of tetrads by the total number of cells at indicated time points.

### Tetrad dissection and spore viability analysis

After observing a sufficient number of tetrads in SPM by regular brightfield microscope, 1 ml SPM was harvested in a 1.5 ml microfuge tube, resuspended in 1 ml of spheroplasting solution (1.2 M sorbitol, 0.1 M phosphate buffer pH 7.5) and incubated with 5 μl of 10 mg/ml Zymolyase 20T (MP Biomedicals, Cat# 320921) for 20 min at 30 °C. After incubation, the tube was kept in ice until dissection. A loop full of this cell suspension was streaked on the middle of the YPD plate (90 mm plate), and tetrads were dissected using a Nikon Eclipse Ci microscope with a micromanipulator (Micro Video Instruments Inc). Plates were incubated for 48 to 72 h at 30 °C. Spore viability (%) was determined after tetrad dissection by dividing the total number of spores germinated by the total number of spores planted, multiplied by 100.

### Chromatin spread

Chromosome spreads were prepared as previously reported (48) with minor modifications. 5 ml of cells from SPM were harvested at different time points. Spheroplasting was carried out by incubating cells in ZK buffer (25 mM Tris-Cl, 0.8 M KCl; pH 7.5) with dithiothreitol (DTT; final concentration 50 mM) for 2 min at room temperature with gentle mixing. Zymolyase 20T was added at 5 μl (stock: 20 mg/ml) and incubated for 30 min at 30 °C. The reaction was halted by adding 1 ml of ice-cold STOP solution (0.1 M MES, 1 mM EDTA, 0.5 mM MgCl_2_, pH 6.4). Spheroplasts were pelleted by centrifugation at 1690*g* for 3 min and resuspended in 100 μl of STOP solution. Glass slides were pretreated by boiling in 0.01% HCl for 10 min, followed by an ethanol rinse. Chromosome spreading was performed by sequentially adding 20 μl of spheroplast suspension, 40 μl of fixative solution (4% paraformaldehyde, 3.4% sucrose), 80 μl of 1% Lipsol, and a final 80 μl of fixative solution to each slide. After gentle mixing, the spheroplasts were evenly distributed across the slide using a pipette tip. Slides were allowed to dry overnight at room temperature. Fixed slides were washed with 2 ml of 0.2% Kodak Photo-Flo 200 (Kodak Alaris Inc, Cat# 1464510), followed by a wash in TBS (25 mM Tris-Cl, 136 mM NaCl, 3 mM KCl, pH 7.4) for 5 min. Slides were blocked in TBS containing 0.1% BSA to minimize non-specific binding for 15 min. Primary antibody incubation was performed for 2 h using one of the following antibodies: Rabbit Anti-Zip1, Rabbit Anti-Rad51, Rabbit Anti-Msh5 (gifted by Prof. Akira Shinohara, Osaka University, Japan; 1:1000 dilution). Slides were washed twice with TBS and were incubated for 2 h with pre-adsorbed secondary antibodies, TRITC-conjugated goat anti-rabbit (Jackson Immuno Research, Cat# 111-025-144 RRID: AB_2337932, 1:1000 dilution). Slides were washed twice in TBS for 5 min each. A mounting medium, Vectashield with DAPI (Vector Laboratories, Cat# H-1200), was used, and the slides were sealed with a transparent nail paint.

### Indirect immunofluorescence

Immunofluorescence was performed as previously reported (29) with minor modifications. Cells were fixed with 37% formaldehyde for 2 h at RT. The fixed cells were washed once with PBS and once with spheroplasting buffer (1.2 M sorbitol, 0.1 M phosphate buffer pH 7.5), and were resuspended in the same solution. Cells were spheroplasted using 5 μl of zymolyase 20T in the presence of 10% β-mercaptoethanol for 45 min at 30 °C. The spheroplasts were washed with spheroplasting solution and transferred to poly-L-lysine-coated slides having Teflon-coated wells (Fisher Scientific, Cat# NC9811708). The spheroplasts were flattened and permeabilized by immersing the slide in methanol for 5 min and acetone for 30 s. Blocking was done using 10 mg/ml BSA in PBS for 15 min. All the primary and secondary antibodies were diluted using antibody dilution buffer (10 mg/ml BSA in PBS). Spheroplasts were incubated with primary antibodies for 1 h, followed by washing with PBS several times, and incubated with secondary antibodies for 1 h in the dark. After several washes with PBS, samples were incubated with a mounting medium, Vectashield with DAPI. The slides were sealed with a transparent nail polish. The following antibodies were used at the indicated dilutions: Rat anti-tubulin antibody (BioRad, Cat# MCA78G, RRID: AB_325005, 1:200 dilution) and TRITC-conjugated goat anti-rat antibody (Jackson ImmunoResearch, Cat# 112-025-167, RRID: AB_2338122, 1:1000dilution).

### Fluorescence microscopy and live cell imaging

All fluorescence microscopy images were acquired using a Leica DMi8 inverted fluorescence microscope, equipped with a 63X oil immersion objective lens, NA=1.40, LED illumination (395 nm for DAPI, 475 nm for GFP, 555 nm for TRITC), a 16-bit Photometric Prime 95B sCMOS camera (Teledyne Photometrics) and controlled by Leica LAS X software. Z-stack images were acquired as per Nyquist sampling criteria. Images were processed by ImageJ for brightness and contrast adjustment, maximum intensity projection, channel merging, and cell counting.

For arresting cells at mitotic metaphase (Fig. S3), we added Benomyl (Sigma Aldrich,Cat# 381586) at 30 μg/ml in mitotic culture and incubated for 2 h to arrest the cells. For control experiments, an equal volume of DMSO was added.

### FACS Analysis

1.5 ml SPM culture was harvested and fixed with 70% ice-cold ethanol for overnight. Fixed cells were pelleted at 21913*g* for 2 min and resuspended in 50 mM Tris-HCl, pH 7.5. 6.25 μl of RNAseA (Invitrogen, Cat# 12091021; stock: 20 mg/ml) was added and incubated for 2 h at 37 °C. 20 μl of proteinase K (Gene to protein, Cat# BUF28-5; 10 mg/ml) was added and incubated at 50 °C for 1 h. The sample was washed once with 50 mM Tris-HCl, pH 7.5. DNA was stained with 25 μg/ml propidium iodide (Sisco Research Laboratories, Cat# 11195) in 200 mM Tris-HCl, pH 7.5, 210 mM NaCl, 78 mM MgCl_2_. Cells were sonicated and diluted in 50 mM Tris-HCl, pH 7.5. Flow cytometric analyses were performed using BD Flowcytometer (BD Biosciences, BD LSRFortessa X-20). >10,000 cells were scored for each sample. Data was processed, analysed and visualized using FlowJo v11.

### ChIP-qPCR

ChIP was performed as previously reported (49) with minor modifications. Approximately 5 × 10^8^cells were used per sample. Crosslinking was carried out with 37% formaldehyde at room temperature for 2 h. Crosslinked cells were washed twice with ice-cold TBS. Lysis was performed in 1.6 ml of lysis buffer (50 mM HEPES-KOH, pH 7.5, 140 mM KCl, 1 mM EDTA, 1% Triton X-100, 0.1% sodium deoxycholate, and protease inhibitor cocktail (Roche, Cat# 4693159001)) using a mini bead beater for 15 cycles of 1 min ON, 3 min OFF (when the samples were kept on ice). Lysates were sonicated using a Bioruptor (Diagenode, Cat# B01020001), at high amplitude, 40 cycles of 30 s ON/30 s OFF to obtain chromatin fragments in the range of 500 to 700 base pairs. Following sonication, lysates were cleared by centrifugation at 21,913*g* for 10 min at 4 °C. A 100 μl aliquot of the clarified lysate was reserved as the input sample (Whole Cell Extract, WCE). The remaining 700 μl was incubated overnight at 4 °C with 5 μl of Anti-mTOR antibody (Enzo Life Sciences, Cat# ALX-215-065-1, RRID: AB_2051920). 50 μl of a ChIP-Grade Protein A/G Magnetic beads (Thermo Scientific, Cat# 26162) were added to each sample, and the tubes were rotated at 4 °C for 2 h. Washes were performed as follows: two washes with 1 ml of IP wash buffer I (50 mM HEPES-KOH, pH 7.5, 150 mM NaCl, 1 mM EDTA, 0.1% sodium deoxycholate, 1% Triton X-100); one wash with 1 ml of IP wash buffer II (IP wash buffer I with 500 mM NaCl); one wash with 1 ml of IP wash buffer III (10 mM Tris, pH 8.0, 250 mM LiCl, 0.5% NP-40, 0.5% sodium deoxycholate, 1 mM EDTA); and one wash with 1 ml of 1x TE buffer (10 mM Tris, pH 8.0, 1 mM EDTA). For elution, the beads were resuspended in 100 μl of elution buffer (50 mM Tris, pH 8.0, 10 mM EDTA, 1% SDS) and incubated at 65 °C for 15 min. The beads were separated using a magnetic block and the supernatant was collected as ‘eluate’ in a fresh tube. This elution step was repeated with 50 μl of elution buffer, and the eluates were pooled. 2.5 μl of proteinase K (Gene to Protein, Cat# BUF28-5, 10 mg/ml) was added to each tube and incubated at 50 °C for 2 h. Crosslinks were reversed by overnight incubation at 65 °C. Input samples (100 μl WCE plus 50 μl elution buffer) were also de-crosslinked overnight at 65 °C. DNA was purified from IP and input samples using the GeneJET PCR purification kit (Invitrogen, Cat# K0702) and eluted in 60 μl of elution buffer. qPCR was performed using a BioRad CFX Opus 96 system (BioRad, Cat# 12011319) with a SYBR Green reaction mixture iQ SYBR Green Supermix (BioRad, Cat# 1708882). The %Enrichment/Input values were calculated as previously reported (28).

ΔCT=CT(ChIP)-[CT(Input)–loge(Inputdilutionfactor)]

% Enrichment/Input = Eˆ(-ΔCT) where E represents the primer efficiency. Primers used for ChIP-qPCR are listed in Table S3.

### Calibrated ChIP-seq

For Calibrated ChIP, the entire protocol was kept the same as the ChIP assay mentioned above. Meiotic synchronization of *S. cerevisiae* and *Saccharomyces mikatae* was performed separately, and the cells were mixed in 10:1 ratio. *S. mikatae* genome was used as an internal reference, as previously described (50).

### Sequencing library preparation

The NEBNext Ultra II DNA Library Prep Kit for Illumina (New England Biolabs, Cat# E7645L) was used to construct ChIP-seq libraries. DNA was initially fragmented to 200 to 280 bp by using the M220 Focused-ultrasonicator. Input of 2 ng to 100 ng DNA for library preparation, fragmented DNA was end-repaired, and adapter ligation adapters were used at a 1:20 dilution, removal of excess adapter using sample purification beads, PCR enrichment of adapter-ligated DNA, and clean-up of PCR products. The cleaned libraries were quantified on a Qubitflurometer (Invitrogen Qubit flurometer) and appropriate dilutions loaded on a high-sensitivity D1000 screen tape on an Agilent 4200 tape station to determine the average size range of the fragments. Sequencing was performed by using NextSeq 2000 with XLEAP-SBS chemistry through the miBiome Therapeutics LLP.

### Bioinformatic analysis of illumina sequence data

Calibrated ChIP-seq analysis was done as described in the earlier studies (51, 52, 53). The quality of the raw sequencing reads from rapamycin-treated, DMSO-treated (untreated control), and their corresponding input samples was checked using FastQC (https://www.bioinformatics.babraham.ac.uk/projects/fastqc/). Sequencing adapters were trimmed using Trimmomatic (version 0.39) to obtain the processed fastq files. These processed samples were then aligned individually with the *S. cerevisiae* S288c (version 64–1-1, 2011) and *S. mikatae* IFO1815 (GCF_947241705.1) genomes using Bowtie2 (version 2.3.5.1) (54). Reads that did not map to the *S. mikatae* genome were aligned against the *S. cerevisiae* genome to obtain uniquely mapped *S. cerevisiae* reads. Similarly, reads that did not map to the *S. cerevisiae* genome were aligned against the *S. mikatae* genome to obtain uniquely mapped *S. mikatae* reads. These uniquely mapped reads were sorted and indexed using Samtools (version 1.10). The sorted uniquely mapped reads to the *S. cerevisiae* genome were used for further analysis as described below. Genome-wide Rec8 peaks were called using MACS2 (model-based analysis for ChIP-seq, version 2.2.7.1) (55). Peaks were called using a pooled dataset from two replicates for rapamycin-treated and DMSO-treated samples. Further, peaks with a *p* value ≥ 10^−5^ were excluded from the analysis to enhance reliability. For plotting genome-wide Rec8 association to chromosomes, first, the occupancy ratio was calculated using uniquely mapped *S. cerevisiae* and *S. mikatae* reads as described (50). The S288c genome was then partitioned into 10 bp bins, and the RPM-normalized Rec8 reads were counted in these bins. These RPM-normalized counts were multiplied by the occupancy ratio to obtain the calibrated Rec8 read depth at each bin. Further, we averaged these calibrated Rec8 reads from two replicates and smoothed them using the ksmooth function in R. All plots were generated in R. The Wilcoxon ranked-sum test was used to test the statistical significance of the boxplot data.

### Relative gene expression analysis

Cells were harvested at the desired time point and pelleted by centrifugation at 3000*g* for 3 min at 4 °C. The cell pellet was resuspended in 500 μl of TRIzol reagent (Invitrogen, Cat# 15596026) and transferred to DEPC-treated O-ring tubes (Biospec, Cat# 10831) containing ∼500 μl of acid-washed glass beads (Sigma-Aldrich. Cat# G8772). Cells were lysed using a mini bead beater (Unigenetics, Cat# MBB-16) for 4 cycles of 1 min beating followed by 1 min on ice. Following lysis, 500 μl of TRIzol is added to the samples and is incubated at room temperature for 5 min for complete dissociation of nucleoprotein complexes. Cells were centrifuged at 12,000*g* for 1 min, and the supernatant was taken. 200 μl of chloroform was added to each sample, mixed vigorously for 15 s, and incubated at room temperature for 2 to 3 min. Samples were centrifuged at 12,000*g* for 15 min at 4 °C, and the aqueous phase was transferred to a fresh tube. RNA was precipitated by adding an equal volume of absolute isopropanol, incubated on ice for 15 min, and pelleted by centrifugation at 12,000*g* for 15 min at 4 °C. The RNA pellet was washed with 1 ml of 75% ethanol, air-dried briefly, and resuspended in 50 μl of RNase-free water. To remove residual genomic DNA, DNase I (Thermo Fisher Scientific, Cat# EN0521) treatment was given for 15 min at 37 °C, followed by phenol–chloroform purification and ethanol precipitation to remove the enzyme and reaction components. RNA concentration and purity were assessed using a NanoDrop spectrophotometer (Eppendorf, Cat#D30), and integrity was confirmed by agarose gel electrophoresis.

One μg of total RNA was used for the cDNA synthesis using the iScript cDNA Synthesis Kit (BioRad, Cat# 1708891), following the manufacturer’s instructions. Briefly, the RNA was mixed with a reaction master mix containing reverse transcriptase, oligo(dT) primers, random hexamers, dNTPs, and buffer components in a final volume of 20 μl. The reverse transcription reaction was carried out in a thermal cycler under the following conditions: 5 min at 25 °C (primer annealing), 20 min at 46 °C (reverse transcription), and 1 min at 95 °C (enzyme inactivation). The resulting cDNA was diluted 1:5 with nuclease-free water and used as a template for qPCR analysis.

qPCR was performed using a BioRad Real-Time PCR system (BioRad CFX Opus, Cat#12011319) with a SYBR Green reaction mixture (BioRad, Cat#1708882). The gene expression of the housekeeping gene *ACT1* was quantified for the normalization. To confirm the absence of contaminating genomic DNA in cDNA preparations, reverse transcriptase negative samples (-RT) were prepared for each cDNA sample, which produced the Ct value difference of ≥10 cycles between “-RT” and “+RT” samples, indicating negligible amount of genomic DNA contamination in all cDNA samples. Primers used for the qPCR assay are listed in Table S3.

### Fluorescence recovery after photobleaching (FRAP)

The FRAP assay was carried out as described elsewhere with a few modifications (56, 57). Briefly, pachytene arrested cells (GMY35) were harvested from meiotic cultures (SPM) at 6 h from DMSO-treated and 8 h from Rapamycin treated flasks. Cells were immobilized on concanavalin A–coated glass-bottom dishes and mounted under 1% agarose pads prepared in sporulation medium.

Live-cell imaging was performed using a Leica TCS SP8 confocal microscope equipped with a 63X 1.4 NA oil immersion objective and 20X digital zoom. Imaging regime: pre-bleach: 3 frames at every 10 s with 0.5% laser, bleach: 1 frame for 1290 ms with 100% laser, recovery for 30 frames at every 10 s with 0.5% laser. For photobleaching, a circular region of interest (ROI) of an area of 0.4 μm^2^ was made.

Fluorescence intensity measurements were performed using ImageJ. For each cell, three ROIs were defined (i) the bleached region, (ii) the nuclear region as a reference ROI, and (iii) a background ROI. All the signals were corrected for background fluorescence at each time point.

For quantification of FRAP data, a free web tool, EasyFRAP (https://easyfrap.vmnet.upatras.gr/) was used with default parameters, full scale normalization, and mean curve fitting (58).

### Western Blotting

Western Blotting was performed to detect the Rec8 abundance in both DMSO and Rapamycin-treated conditions. 12 ml of meiotic cultures were collected in a centrifuge tube, and cells were harvested at 1690*g* for 3 min. Cell pellets were washed with ice-chilled distilled water and transferred into a 1.5 ml microcentrifuge tube. 150 μl of cell lysis buffer (50 mM HEPES-pH7.5, 140 mM KCl, 1 mM EDTA, 1% Triton-X-100, 0.1% sodium deoxycholate, 1X Protease inhibitor cocktail, 1 mM phenylmethanesufonyl fluoride, 10 mM sodium fluoride) and an equal volume of 0.5 mm glass beads were added to each screw-capped “O” ring tubes. The cells were ruptured by 5 cycles of bead beating (1 min ON and 2 min OFF) at high-speed using BioSpec Mini Beadbeater-16, USA. Whole cell lysates were collected into fresh 15 ml Falcon tubes. The cell lysate was transferred into a 1.5 ml microcentrifuge tube and spun at 21,913*g* for 10 min at 4 °C. Supernatant was gently transferred into the fresh 1.5 ml microcentrifuge tube. The crude protein extract was mixed with 4× Laemmli sample loading buffer containing 750 mM of 2-mercaptoethanol and boiled at 95 °C for 5 min. After boiling, the tubes were kept at room temperature to cool down. 35 μl of protein extracts were loaded onto 10% SDS-polyacrylamide gel and resolved. The resolved SDS-polyacrylamide gel was transferred to PVDF membrane using standard protocol for western blotting. Blocking was done using 3% BSA for 1 h. The blot was incubated with the primary antibodies (Rabbit-anti Rec8 polyclonal antibody (custom made, kind gift from Akira Shinohara’s lab, University of Osaka, Japan, 1:2000 dilution) and Anti-GAPDH antibody [GA1R] (Abcam, Cat# ab125247, RRID: AB_11129118, 1:3000 dilution) overnight at 4 °C. The blot was incubated with the HRP conjugated goat anti-Rabbit IgG secondary antibody (Jackson ImmunoResearch Cat# 111-035-008, RRID: AB_2337937, 1:10,000 dilution) and HRP conjugated Goat anti-Mouse IgG (H + L) secondary antibody (Jackson Immunoresearch Cat# 115-035-003, RRID: AB_10015289, 1:10,000 dilution) for 1.5 h. The membrane was developed using the Pierce ECL Western Blotting Substrate (Enhanced Chemiluminescence, Thermo Scientific and Cat no. 32109) and imaged using Bio-Rad Hi-sensitivity ChemiDoc Imager. Band intensities were measured using ImageJ.

## Data availability

Sequence data available from the NCBI Sequence Read Archive with BioProject ID PRJNA1290074 (http://www.ncbi.nlm.nih.gov/bioproject/1290074).

## Supporting information

This article contains supporting information (59).

## Conflict of interest

The authors declare that they have no conflicts of interest with the contents of this article.
