# Supplementary material for: The dynamic pool of Rec8-cohesin is crucial for meiotic recombination and transcription regulation in the yeast Saccharomyces cerevisiae
Source: J Biol Chem. 2026 Apr 24;302(6):113064. doi: 10.1016/j.jbc.2026.113064 (PMC13214519; doi:10.1016/j.jbc.2026.113064)
Supplement: Supplementary Data [file mmc1.pdf]

The dynamic pool of Rec8-cohesin is crucial for meiotic recombination and transcription regulation in the yeast *S. cerevisiae*.

Authors: Sheetal Paliwal[1], Partha Dey[1], Akriti Kumari[1], Kirithika Sadasivam[1], Sameer Joshi[2], Ritika Raghuvanshi[3], Rohit Goyal[3], Kaustuv Sanyal[3], K.T. Nishant[2], Akira Shinohara[4], Gunjan Mehta[1]*

Affiliations:
[1]Laboratory of Chromosome Dynamics and Gene Regulation, Department of Biotechnology, Indian Institute of Technology Hyderabad, Kandi, Telangana-502285, India.
[2]School of Biology, Indian Institute of Science Education and Research Thiruvananthapuram, Trivandrum, Kerala-695551, India
[3] Molecular Biology and Genetics Unit, Jawaharlal Nehru Centre for Advanced Scientific Research, Jakkur, Bengaluru, Karnataka-560064, India
[4]Institute of Protein Research, The University of Osaka, Osaka 565-0871, Japan.

***Correspondence:**
gunjanmehta@bt.iith.ac.in

List:

S-2. Fig.S1. FACS for DNA replication progression analysis, Rec8-FRB-GFP fluorescence intensity measurements, and Rec8 transcript and protein levels upon rapamycin treatment.
S-4. Fig.S2. Validation experiments for a stable pool of Rec8-cohesin.
S-6 Fig. S3. Anchor-away–mediated depletion of Smc3-FRB-GFP during mitosis.
S-7. Fig.S4. Calibrated ChIP-seq data for Rec8-FRB-GFP.
S-9. Fig.S5. Immunofluorescence to quantify the cells at different stages of meiosis.
S-10. Fig.S6. Chromatin spread analysis to quantify defects in DSB formation and crossover frequency upon depleting the dynamic pool of Rec8-cohesin.
S-12. Fig.S7. Relative gene expression analysis using RT-qPCR for key meiotic genes.
S-13. Table S1. Rec8 peak locations at the 6h time point in DMSO-treated and Rapamycin-treated samples.
S-68. Table S2. List of yeast strains used in the study.
S-70. Table S3. List of Primers used in the study.
S-74. Table S4. List of plasmids used in the study.

Figure S1

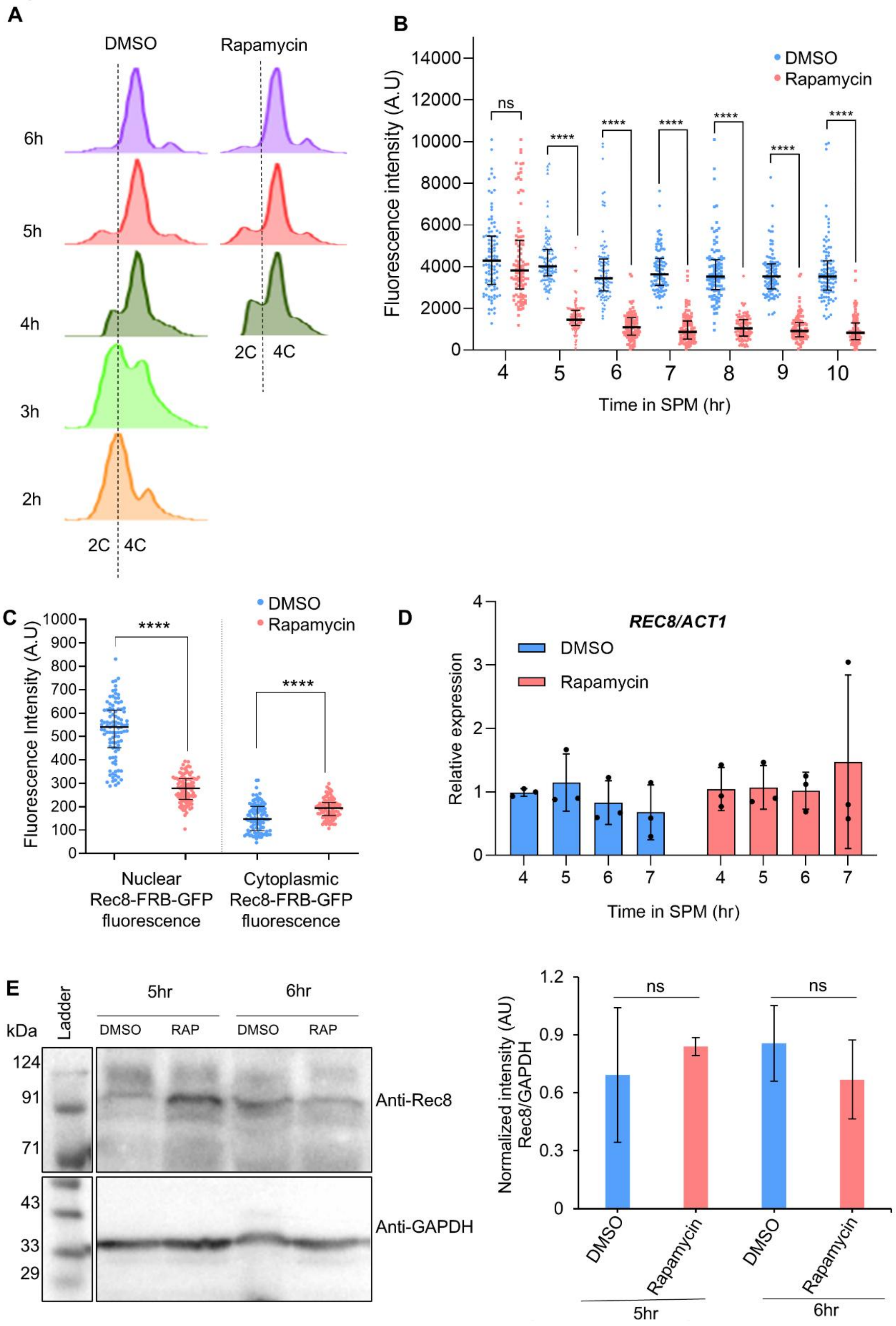
A
DMSO
Rapamycin
6h
5h
4h
3h
2h
2C
4C
B
Fluorescence intensity (A.U)
DMSO
Rapamycin
ns
****
Time in SPM (hr)
C
Fluorescence Intensity (A.U)
Nuclear Rec8-FRB-GFP fluorescence
Cytoplasmic Rec8-FRB-GFP fluorescence
D
REC8/ACT1
Relative expression
Time in SPM (hr)
E
Ladder
kDa
5hr
6hr
DMSO
RAP
Anti-Rec8
Anti-GAPDH
Normalized intensity (AU) Rec8/GAPDH
Rapamycin

Figure S1. FACS for DNA replication progression analysis, Rec8-FRB-GFP fluorescence intensity measurements, and Rec8 transcript and protein levels upon rapamycin treatment. (A) FACS for DNA replication progression analysis. Rapamycin was added at 3 h in SPM. Samples were collected at the indicated time points from SPM. DNA content was quantified by FACS using propidium iodide staining. >10,000 cells were scored. Representative histograms show changes in DNA content (2C to 4C) over time. The experiment was repeated twice, but the data from only one set is presented.

(B)The dot plot representation of Figure 1D. The nuclear fluorescence intensity for Rec8-FRB-GFP (after background subtraction) for each nucleus. Horizontal lines indicate the median, and error bars denote the interquartile range. Statistical significance was assessed using two-tailed Mann–Whitney U tests. ****p<0.0001, ns: not significant

(C) The nuclear and cytoplasmic fluorescence intensity for Rec8-FRB-GFP (after background subtraction) was quantified from 6 h sample (rapamycin was added at 4 h). Nuclear and cytoplasmic intensities were measured using an identical-sized region of interest (ROI). Horizontal lines indicate the median, and error bars denote the interquartile range. Statistical significance was assessed using two-tailed Mann–Whitney U tests. ****p <0.0001

(D) Relative gene expression analysis using RT-qPCR. mRNA was extracted from the meiotic samples harvested at the indicated time points, and the level of *REC8* mRNA was measured using RT-qPCR. The expression of the housekeeping gene *ACT1* was used for normalization. The experiment was repeated thrice. Error bars represent SD. Individual data points are overlaid on the bar graph, with each point representing a biological replicate.

(E) Quantification of Rec8-FRB-GFP protein levels using western blotting. Band intensities were quantified using ImageJ. The experiment was repeated twice, and the error bars represent the SD. Statistical analysis was performed using an unpaired t-test with Welch's correction.

Figure S2

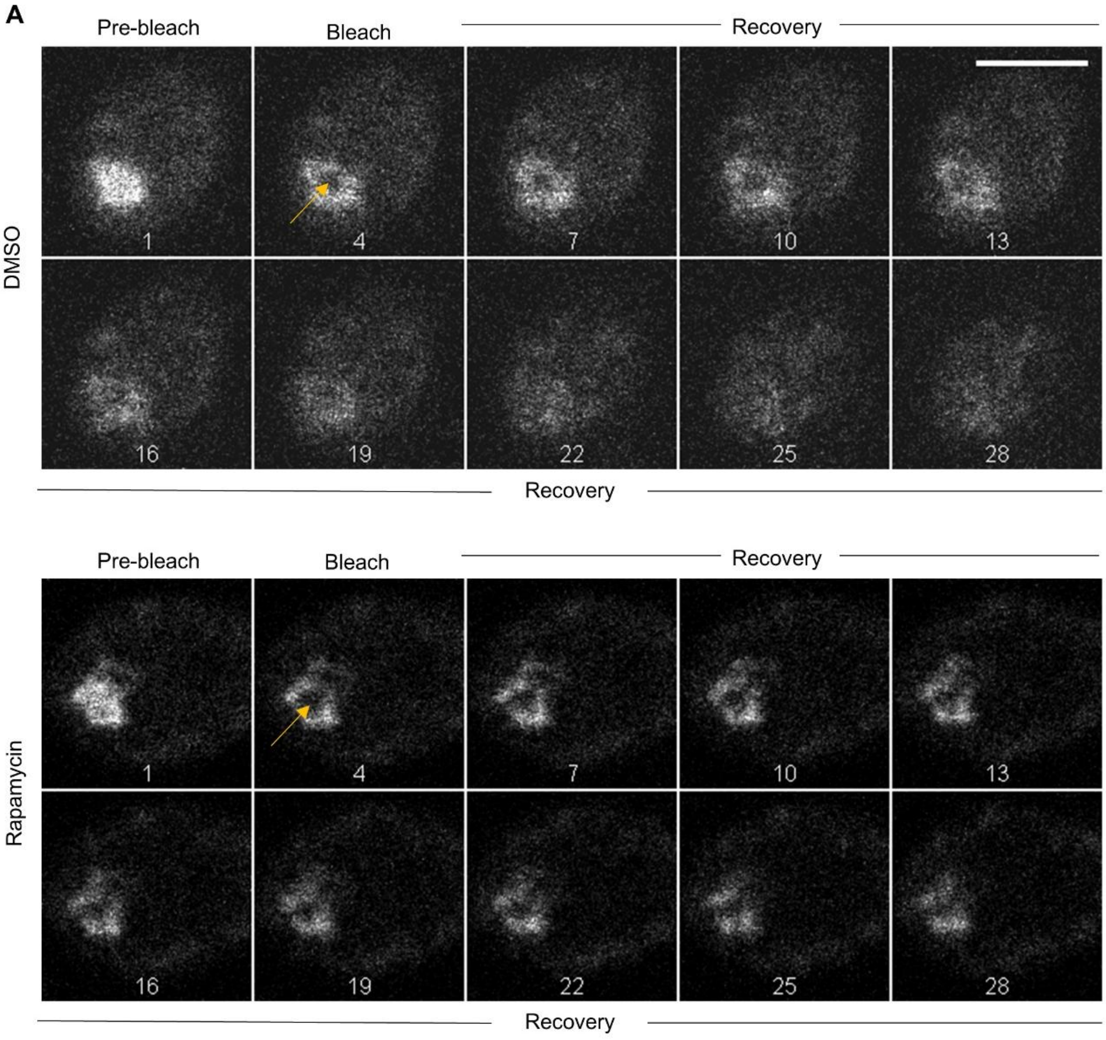

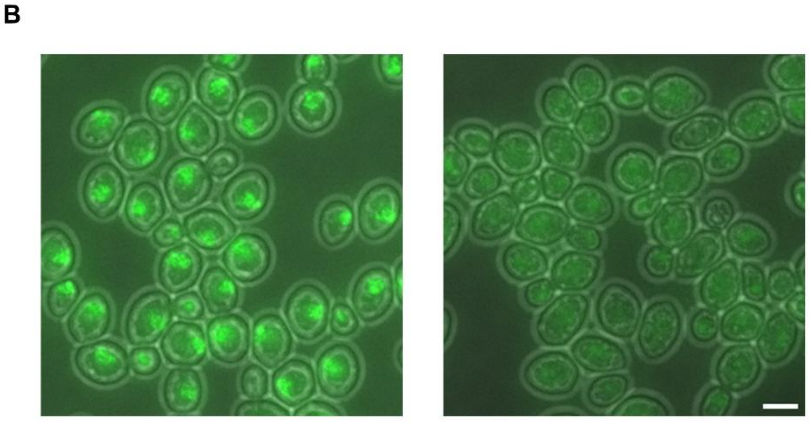

DMSO added at 0 hr in SPM

Rapamycin added at 0 hr in SPM

Figure S2. Validation experiments for a stable pool of Rec8-cohesin.

(A) FRAP analysis for Rec8-FRB-GFP**.** Rapamycin/DMSO was added at 4 h in SPM, and the samples were harvested for FRAP analysis at 6 h. Frame numbers are shown at the bottom of each frame. Movies were acquired with 10 s time interval between the consecutive frames. Scale: 5 μm.

(B) Live cell imaging to visualize the distribution of Rec8-FRB-GFP in the nucleus and cytoplasm. The rapamycin/DMSO was added at 0 h in SPM, and the samples were taken for imaging at 4 h from SPM. Scale: 5 μm.

Figure S3

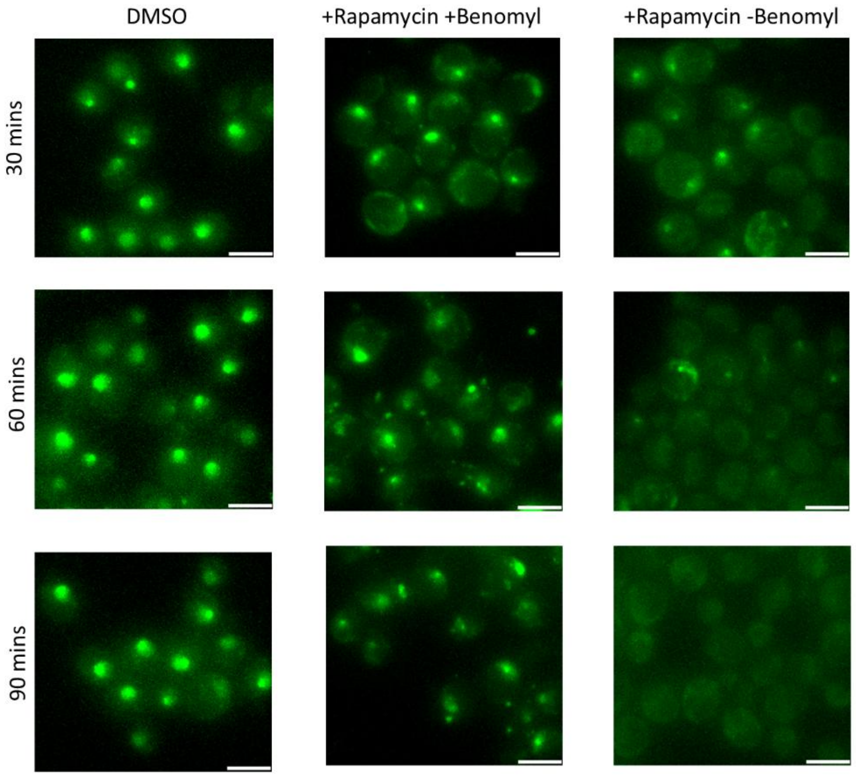

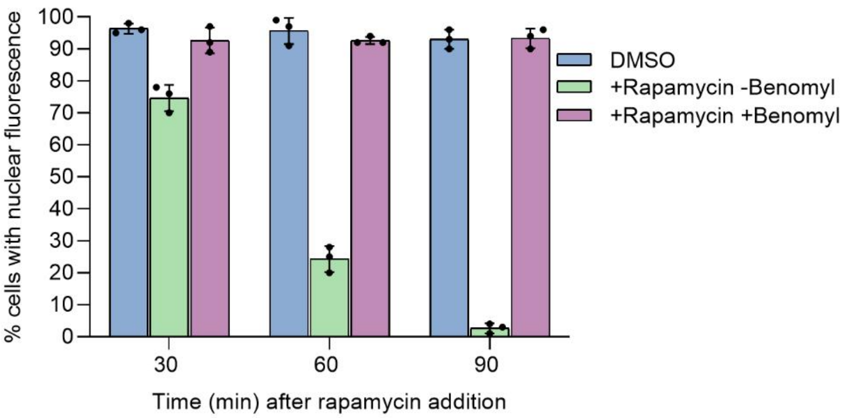

Figure S3. Anchor-away–mediated depletion of Smc3-FRB-GFP during mitosis. Live cell imaging for Smc3-FRB-GFP under various treatments and time points from mitotic culture. The bar graph represents the quantification of the number of cells showing the Smc3-FRB-GFP signal in the nucleus. The experiment was repeated three times. Error bars represent SD. Individual data points are overlaid on the bar graph, with each point representing a biological replicate. n>300. Scale bar: 5 μm

Figure S4

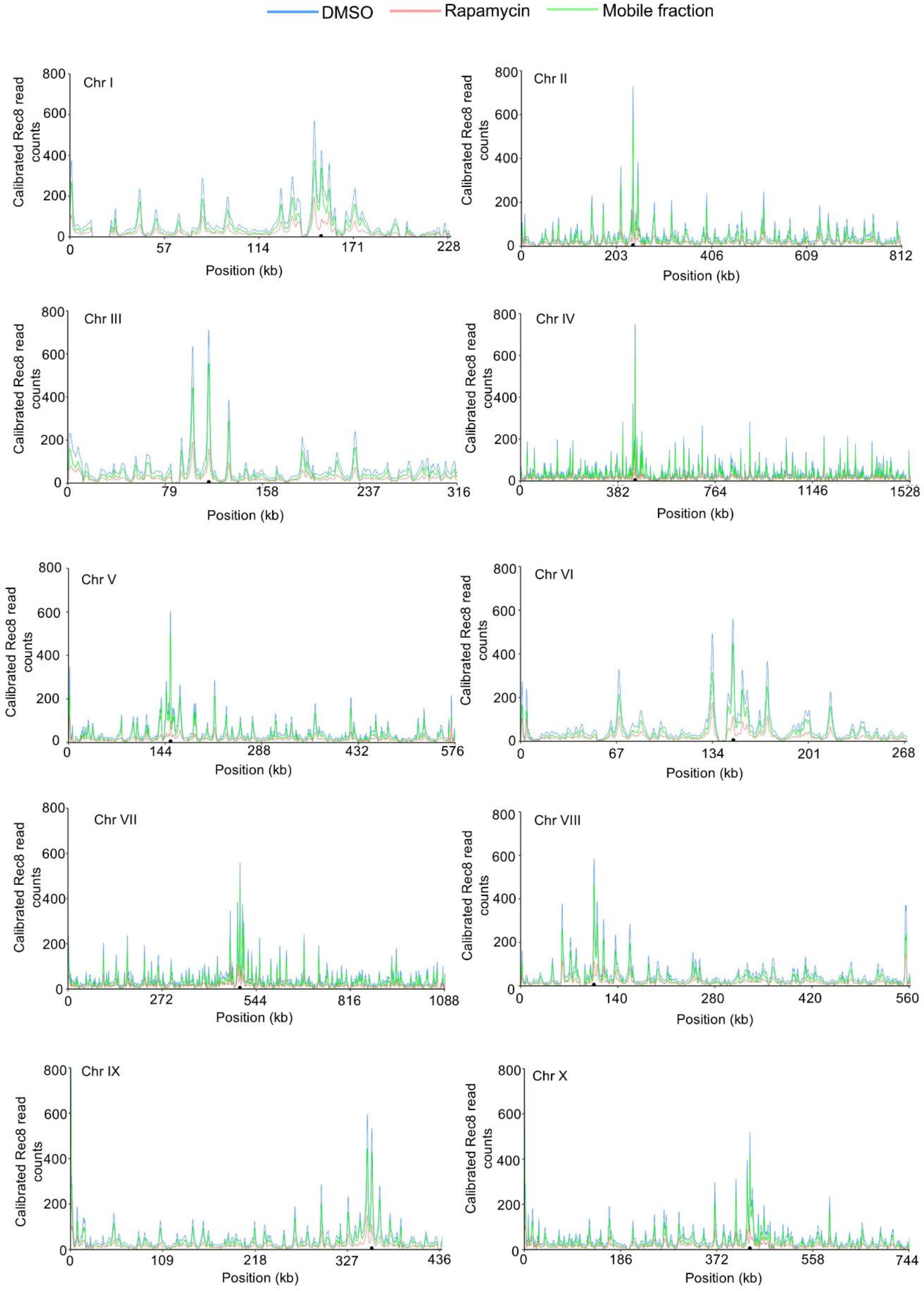

DMSO
Rapamycin
Mobile fraction
Chr I
Chr II
Chr III
Chr IV
Chr V
Chr VI
Chr VII
Chr VIII
Chr IX
Chr X
Calibrated Rec8 read counts
Position (kb)

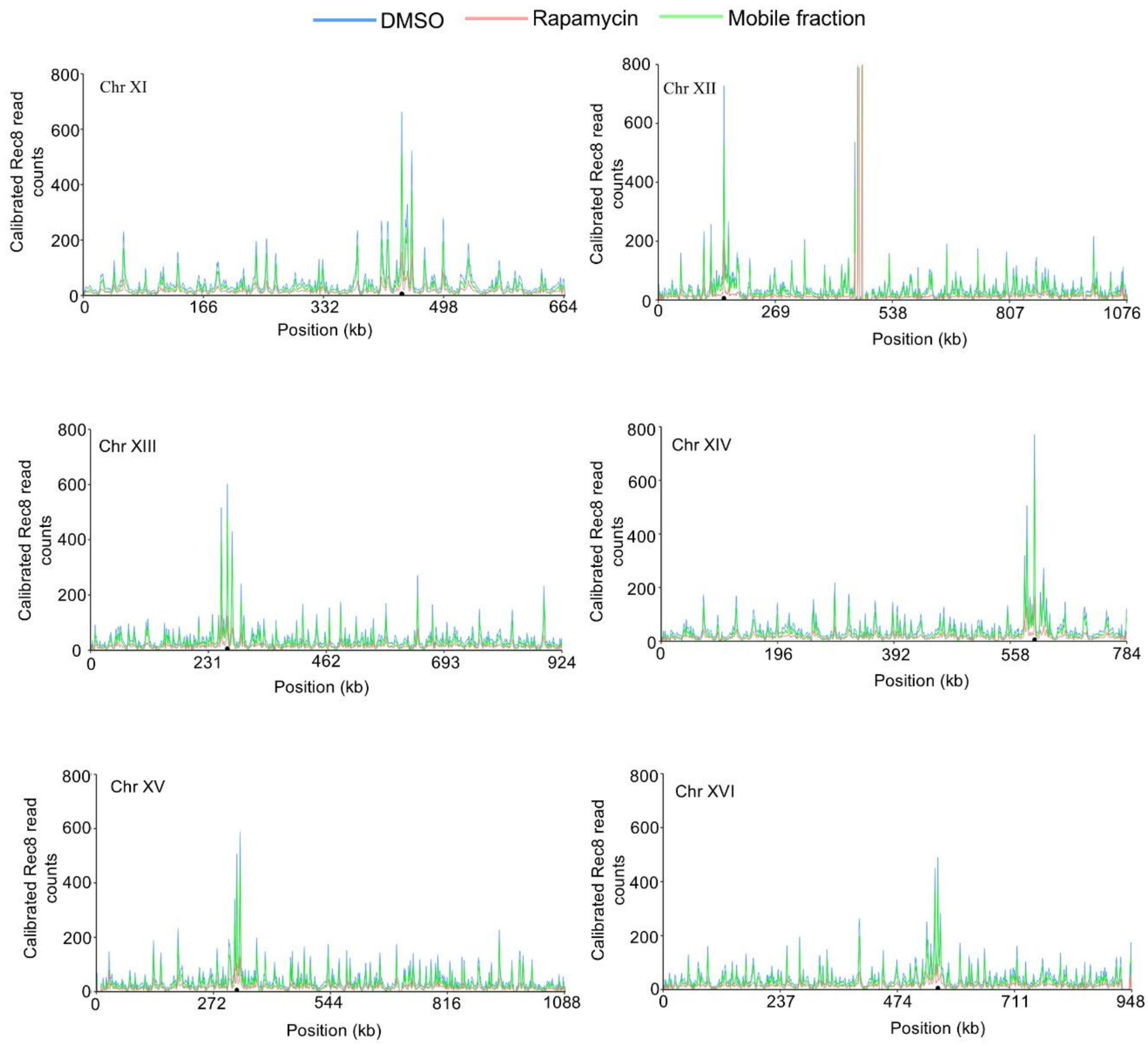

Figure S4. Calibrated ChIP-seq data for Rec8-FRB-GFP.

Representative Rec8-ChIP-seq profile of all 16 chromosomes of yeast. The black dot indicates the centromere. The data are from two independent biological replicates. The mobile fraction was calculated by subtracting the stable pool (Rapamycin-treated sample) from the dynamic+stable pool (DMSO-treated sample).

Figure S5

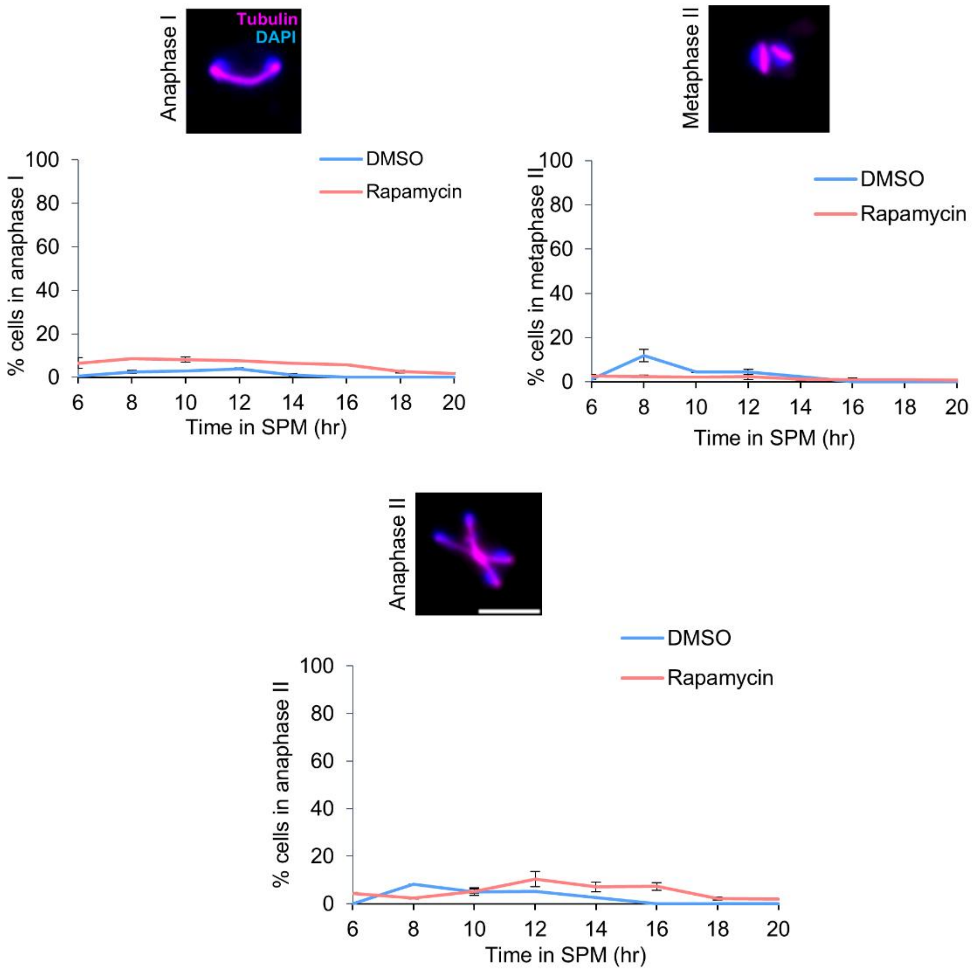

Figure S5. Immunofluorescence to quantify the cells at different stages of meiosis. Immunofluorescence using anti-tubulin antibodies and DAPI to visualize the relative positions of spindles and nuclei to identify the stages of meiosis. The experiment was repeated twice. More than 100 cells were counted for each time point. Error bars represent standard deviation (SD). Scale: 5 μm.

Figure S6

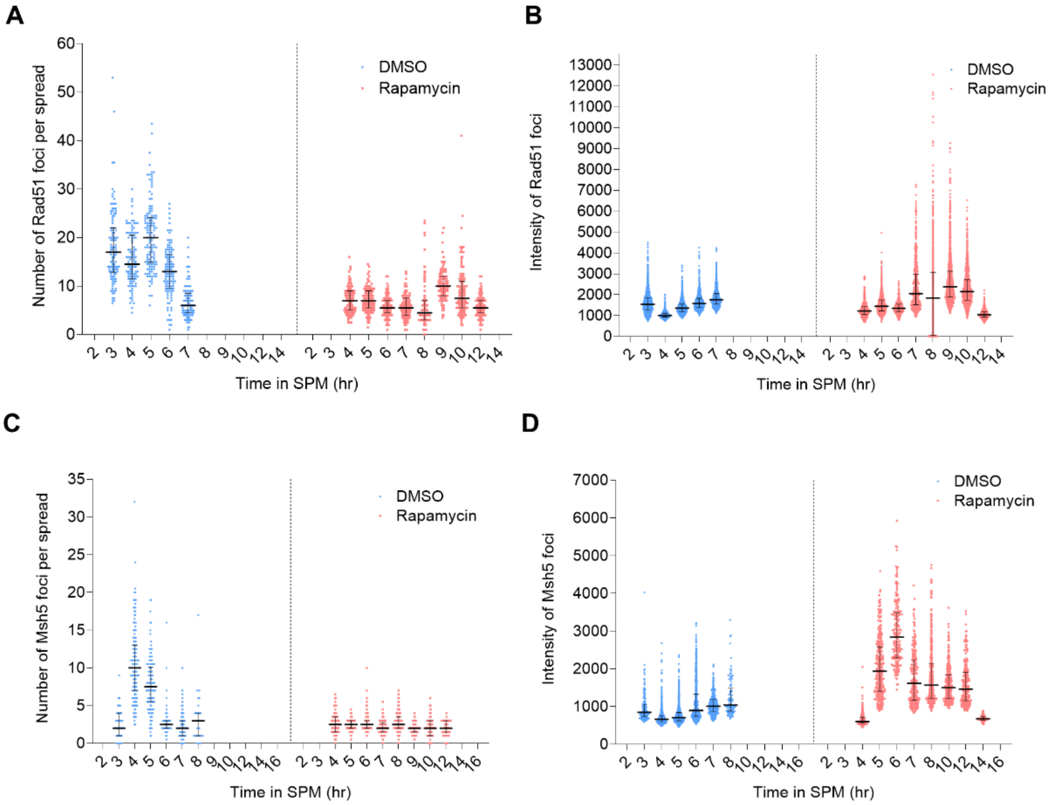

Figure S6. Chromatin spread analysis to quantify defects in DSB formation and crossover frequency upon depleting the dynamic pool of Rec8-cohesin.

(A) Chromatin spread using anti-Rad51 antibody and DAPI staining. The dot plot represents the number of Rad51 foci per spread at different time points. Rad51 foci were quantified from chromatin spreads prepared from DMSO- or rapamycin-treated cells. For each condition, >100 nuclei were scored per experiment, and two independent biological replicates were analyzed. Each dot represents the number of Rad51 foci in a single nucleus. Horizontal lines indicate the median, and error bars denote the interquartile range. The experiment was repeated twice.

(B) Rad51 fluorescence intensity was quantified from chromatin spreads prepared from DMSO- or rapamycin-treated cells from Figure S5A. For each condition, >100 nuclei were analyzed per experiment, and two independent biological replicates were examined. Each dot represents the integrated Rad51 signal intensity from a single nucleus. Horizontal lines indicate the median, and error bars denote the interquartile range.

(C) Chromatin spread using anti-Msh5 antibody and DAPI staining. The dot plot represents the number of Msh5 foci per spread at different time points. Msh5 foci were quantified from chromatin spreads prepared from DMSO- or rapamycin-treated cells. For each condition, >100 nuclei were

scored per experiment, and two independent biological replicates were analyzed. Each dot represents the number of Msh5 foci in a single nucleus. Horizontal lines indicate the median, and error bars denote the interquartile range. The experiment was repeated twice.

(D) Msh5 fluorescence intensity was quantified from chromatin spreads prepared from DMSO- or rapamycin-treated cells from Figure S5C. For each condition, >100 nuclei were analyzed per experiment, and two independent biological replicates were examined. Each dot represents the integrated Msh5 signal intensity from a single nucleus. Horizontal lines indicate the median, and error bars denote the interquartile range.

Figure S7

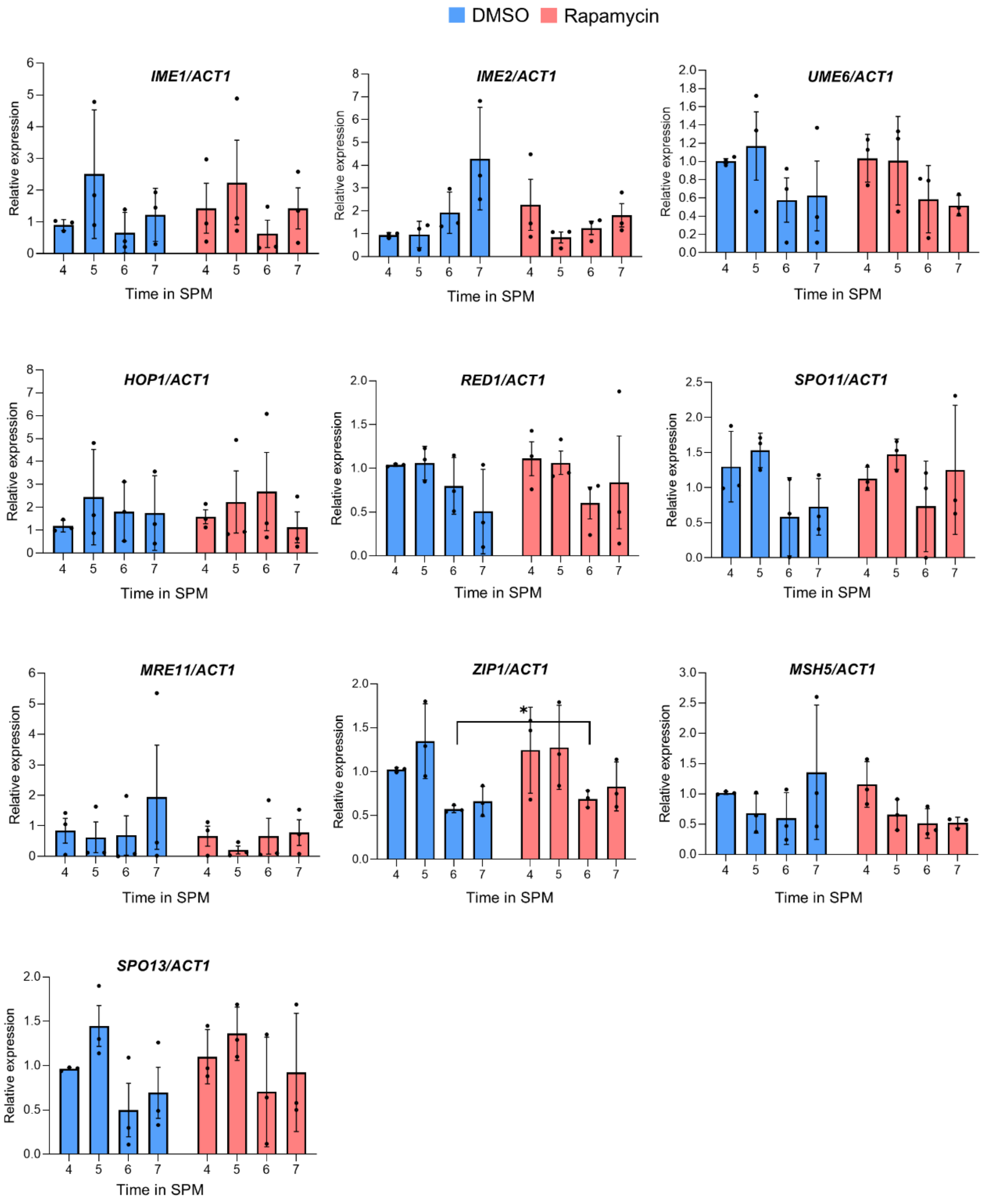

Figure S7. Relative gene expression analysis using RT-qPCR for key meiotic genes. The expression of the housekeeping gene *ACT1* was used for normalization. The experiment was repeated thrice. Error bars represent SD. Individual data points are overlaid on the bar graph, with each point representing a biological replicate.

Table S1.

The p-value indicates statistical significance of the peaks

q value indicates the adjusted p value using Benjamini-Hochberg correction

Rec8 peak locations at the 6h time point in the DMSO-treated sample.

Chromosome	Start	End	Fold enrichment	p value	q value
chr1	417	1405	2.48301	0	0
chr1	10183	11415	1.46559	3.25087E-15	2.66502E-14
chr1	39640	43347	5.59214	0	0
chr1	50493	53151	2.9725	1.2023E-185	4.4668E-184
chr1	64459	66552	2.42523	6.8234E-108	1.6255E-106
chr1	78266	81926	5.06278	0	0
chr1	82559	83537	1.47071	6.38852E-17	5.44879E-16
chr1	93000	96367	3.33519	2.4322E-293	1.4158E-291
chr1	96623	98416	2.12512	3.6779E-76	6.78891E-75
chr1	123923	129670	4.82907	0	0
chr1	130007	130842	1.51	4.29438E-21	4.00682E-20
chr1	131136	131444	1.57846	1.75914E-24	1.74944E-23
chr1	131694	139362	5.916	0	0
chr1	143816	160193	10.0293	0	0
chr1	166218	179009	4.97529	0	0
chr1	195305	196642	1.84084	8.28705E-45	1.11635E-43
chr1	196888	197328	2.12809	3.18787E-65	5.32353E-64
chr1	197709	198144	1.4487	1.18059E-13	9.30251E-13
chr1	228110	228364	2.2763	1.0074E-92	2.14141E-91
chr10	44	2574	3.16017	0	0
chr10	7510	8067	1.51863	8.6278E-75	1.5729E-73
chr10	14479	17283	1.77538	1.4454E-113	3.581E-112
chr10	27142	28524	1.67525	6.36209E-60	1.00972E-58
chr10	39098	41945	2.20064	1.7402E-79	3.30598E-78
chr10	66616	68144	1.93655	1.92265E-52	2.82879E-51
chr10	76538	77581	1.61854	2.67055E-23	2.59836E-22
chr10	86402	88069	1.50407	9.20238E-19	8.18653E-18
chr10	91774	92203	1.58524	3.20848E-21	3.00123E-20
chr10	98843	99126	1.63187	3.7145E-24	3.67198E-23
chr10	99454	100450	1.86642	3.155E-43	4.16294E-42
chr10	106366	107856	1.61456	4.77309E-24	4.70869E-23

chr10	121773	122603	1.48548	1.08893E-16	9.24272E-16
chr10	124006	127720	2.86116	1.9099E-169	6.5615E-168
chr10	146998	148570	2.08859	6.57658E-71	1.15664E-69
chr10	162200	166286	3.27224	2.6977E-273	1.4588E-271
chr10	166787	168686	1.98194	9.64939E-56	1.4696E-54
chr10	168943	170053	1.33341	4.56951E-10	3.2526E-09
chr10	170395	170724	1.45543	3.14485E-15	2.57929E-14
chr10	209325	211243	3.014	1.7498E-202	6.9984E-201
chr10	233887	235622	2.18668	2.58285E-74	4.68705E-73
chr10	249610	253383	3.39299	1.0691E-272	5.7677E-271
chr10	268726	275657	3.4904	6.166E-294	3.5892E-292
chr10	283367	284035	1.55878	7.00326E-22	6.63437E-21
chr10	297659	301351	2.86968	1.0789E-203	4.3451E-202
chr10	306749	309135	2.00955	1.1298E-67	1.92975E-66
chr10	324104	328603	2.24323	2.30144E-87	4.68813E-86
chr10	346070	348609	1.97115	1.72703E-57	2.67609E-56
chr10	367038	371793	5.80163	0	0
chr10	383339	388814	2.51527	5.6234E-122	1.4723E-120
chr10	395000	396027	1.47789	1.2543E-16	1.06316E-15
chr10	396800	399414	1.73663	1.54028E-35	1.83021E-34
chr10	407684	412443	7.31682	0	0
chr10	416260	420061	1.85833	8.82673E-41	1.12772E-39
chr10	428419	444899	12.7752	0	0
chr10	446819	448502	1.95295	2.78163E-50	3.99853E-49
chr10	450692	451002	1.33547	1.27198E-08	8.63754E-08
chr10	451584	453651	4.30709	0	0
chr10	456167	456550	1.43499	2.86748E-13	2.23615E-12
chr10	456935	468827	5.60622	0	0
chr10	469176	472351	2.44027	1.374E-117	3.4995E-116
chr10	484062	486371	1.90867	1.30347E-48	1.8395E-47
chr10	498951	501507	2.61691	1.6482E-135	4.6881E-134
chr10	508530	511727	2.02486	2.88669E-62	4.68274E-61
chr10	515233	516959	1.67074	5.90201E-29	6.34016E-28
chr10	518293	519404	1.334	1.37196E-08	9.30144E-08
chr10	537191	537780	1.32347	3.23735E-08	2.16576E-07
chr10	552170	553756	1.2855	2.63196E-07	1.70322E-06
chr10	557461	558187	1.47763	1.22546E-15	1.01485E-14

chr10	564795	569686	2.23262	1.17085E-82	2.28823E-81
chr10	569929	570416	1.26745	2.04918E-07	1.33168E-06
chr10	574122	576052	2.22126	1.53391E-77	2.86484E-76
chr10	588819	592496	5.47775	0	0
chr10	601980	603615	1.69253	3.83796E-31	4.27071E-30
chr10	628351	630125	2.07012	3.56205E-61	5.72005E-60
chr10	630440	630683	1.40873	9.58297E-12	7.17133E-11
chr10	650499	651212	1.28884	6.74714E-07	4.29655E-06
chr10	652693	655569	2.64283	2.6546E-133	7.4473E-132
chr10	655862	657005	1.72418	1.52827E-30	1.68461E-29
chr10	687831	690640	2.30422	3.09813E-91	6.51028E-90
chr10	711070	713212	1.94222	4.60363E-64	7.59801E-63
chr10	741421	741680	1.7018	6.9952E-29	7.50758E-28
chr11	23961	27822	1.91646	5.52205E-46	7.55266E-45
chr11	41434	41724	1.24728	3.48706E-06	2.15367E-05
chr11	41991	44130	2.90713	1.2942E-178	4.6452E-177
chr11	54148	58664	4.14575	0	0
chr11	77276	77549	1.30275	1.61879E-07	1.05619E-06
chr11	85536	86916	2.33686	2.70209E-96	5.90609E-95
chr11	105802	106524	1.44537	1.72425E-13	1.35301E-12
chr11	106793	107706	2.70423	1.0593E-131	2.9444E-130
chr11	108047	109169	1.84834	5.72532E-40	7.23602E-39
chr11	109573	109902	2.43448	2.36265E-98	5.24204E-97
chr11	110222	111757	3.155	3.0974E-196	1.2078E-194
chr11	129358	132681	3.22702	8.414E-228	3.7497E-226
chr11	157751	158320	1.25528	8.06529E-06	4.90208E-05
chr11	158575	160494	1.89829	4.25011E-44	5.67283E-43
chr11	167151	168221	1.33912	1.10864E-09	7.79579E-09
chr11	182991	188244	2.77684	1.8793E-149	5.781E-148
chr11	212089	212700	1.29217	5.1625E-07	3.30293E-06
chr11	216578	217183	1.25554	8.55205E-06	5.1923E-05
chr11	217872	218996	1.48866	1.05172E-15	8.72369E-15
chr11	220877	222541	2.03855	7.92501E-64	1.30527E-62
chr11	234382	234908	1.32929	7.37836E-09	5.04929E-08
chr11	235512	240796	4.08818	0	0
chr11	251259	254876	4.11415	0	0
chr11	264484	267692	3.08471	2.5351E-206	1.0328E-204

chr11	292121	293904	1.46088	8.35603E-18	7.27612E-17
chr11	302396	302860	1.5481	2.87409E-19	2.58464E-18
chr11	312451	313320	1.25719	3.31642E-06	2.04989E-05
chr11	320288	321182	1.46925	8.97842E-15	7.27947E-14
chr11	324272	326372	3.61473	1.194E-274	6.5013E-273
chr11	329994	332667	3.47157	2.1232E-250	1.0447E-248
chr11	335821	336269	1.40873	9.58297E-12	7.17133E-11
chr11	373242	380267	4.91179	0	0
chr11	387827	390950	1.84168	1.97379E-39	2.47628E-38
chr11	393891	395155	1.35587	2.15482E-10	1.54882E-09
chr11	398473	399264	1.38877	1.9002E-11	1.4101E-10
chr11	410564	416740	5.18891	0	0
chr11	418217	423638	5.23799	0	0
chr11	429862	430922	1.26937	1.82898E-06	1.14362E-05
chr11	431776	432444	1.91494	1.63606E-45	2.22485E-44
chr11	432760	434561	3.17695	9.2257E-214	3.8726E-212
chr11	435725	449848	15.1996	0	0
chr11	452122	456354	11.8494	0	0
chr11	470155	473993	3.69557	0	0
chr11	481401	482580	1.45198	4.75773E-16	3.97741E-15
chr11	484397	486350	1.91034	1.0311E-46	1.42364E-45
chr11	495328	499590	5.98931	0	0
chr11	500583	502084	1.93732	2.5293E-49	3.59749E-48
chr11	520855	523052	1.55208	3.3458E-25	3.37132E-24
chr11	529476	535664	3.40099	2.8119E-285	1.5885E-283
chr11	535964	537624	1.48001	3.20848E-18	2.81968E-17
chr11	570091	570803	1.6244	1.48115E-26	1.52827E-25
chr11	572738	577028	2.39897	2.5586E-109	6.166E-108
chr11	595728	597596	1.89254	9.00741E-46	1.22857E-44
chr11	602186	604183	1.56083	2.82813E-21	2.64789E-20
chr11	632410	634395	2.35455	3.36047E-89	6.94864E-88
chr11	637869	638833	1.24227	4.5848E-06	2.81722E-05
chr11	659067	659528	1.74707	4.81504E-48	6.74994E-47
chr11	660151	660408	2.26119	4.38834E-80	8.38108E-79
chr11	662616	662866	2.14946	9.68055E-74	1.74783E-72
chr11	663213	664688	2.16698	7.66479E-82	1.48628E-80
chr12	3808	4092	2.11016	8.46642E-71	1.48765E-69

chr12	9333	9627	2.14285	1.99526E-74	3.62493E-73
chr12	31004	31694	1.43538	5.30152E-13	4.10677E-12
chr12	31996	33122	1.65185	1.81384E-25	1.83696E-24
chr12	51049	53668	4.14902	0	0
chr12	91287	91904	1.27885	1.48392E-06	9.31644E-06
chr12	103155	106468	5.58991	0	0
chr12	118970	123260	6.22066	0	0
chr12	126397	128372	1.96902	1.27732E-50	1.84289E-49
chr12	128806	129329	1.96157	1.42495E-49	2.03283E-48
chr12	132911	135454	2.28952	2.41713E-83	4.75335E-82
chr12	137424	140665	1.90438	1.38197E-45	1.88105E-44
chr12	144493	163088	16.13	0	0
chr12	164447	166598	1.66708	1.4067E-32	1.60103E-31
chr12	166906	174938	2.37035	2.22895E-92	4.72498E-91
chr12	175502	179557	1.60134	5.61177E-24	5.52968E-23
chr12	180105	186875	3.95379	0	0
chr12	207502	208771	1.48667	2.94036E-16	2.47059E-15
chr12	209185	211874	2.90577	8.3368E-181	3.0269E-179
chr12	256391	257982	2.14568	2.38397E-69	4.13523E-68
chr12	264319	264788	1.33066	1.47201E-08	9.97034E-08
chr12	265653	269625	2.50837	3.3497E-108	7.9983E-107
chr12	285160	286316	1.58191	5.12035E-21	4.772E-20
chr12	287839	289313	1.33837	3.3266E-09	2.30362E-08
chr12	304680	309207	3.24122	2.8184E-234	1.2912E-232
chr12	334994	338722	4.99077	0	0
chr12	381055	383685	2.39476	3.5975E-117	9.1411E-116
chr12	393577	395429	1.66085	1.97288E-28	2.10136E-27
chr12	419696	422700	2.79561	5.2723E-159	1.71E-157
chr12	425988	426981	1.4977	3.83266E-17	3.28625E-16
chr12	427667	430395	2.3176	2.4581E-85	4.9204E-84
chr12	435259	438751	3.37666	3.0269E-237	1.4028E-235
chr12	450400	450962	1.50129	2.33238E-23	2.27196E-22
chr12	458797	459582	1.11416	1.26503E-56	1.94357E-55
chr12	459900	460782	1.22124	8.2794E-257	4.1976E-255
chr12	467935	468734	1.12307	1.02636E-64	1.70451E-63
chr12	520051	521057	1.57858	8.15643E-21	7.57007E-20
chr12	529174	532316	3.54022	6.6222E-286	3.7497E-284

chr12	563140	564190	1.35472	1.44694E-09	1.01417E-08
chr12	577687	583261	1.88562	5.76634E-46	7.88497E-45
chr12	622102	625298	2.56103	1.5417E-113	3.8194E-112
chr12	625570	626184	1.48699	5.35303E-16	4.46992E-15
chr12	627088	629768	2.37557	1.19399E-94	2.57691E-93
chr12	657016	657528	1.36001	1.75501E-10	1.26497E-09
chr12	661125	664186	4.4146	0	0
chr12	674836	676822	1.7451	5.40505E-32	6.09818E-31
chr12	682265	685509	2.34123	1.04208E-87	2.12912E-86
chr12	693402	697728	1.90114	1.69942E-44	2.28034E-43
chr12	717086	717394	1.32292	3.02315E-08	2.02554E-07
chr12	732253	732627	1.39874	2.73149E-11	2.01739E-10
chr12	733848	734967	4.76904	0	0
chr12	748140	749880	1.92699	2.47799E-48	3.48498E-47
chr12	775462	776562	1.78828	3.69743E-36	4.43302E-35
chr12	793869	794553	1.55527	2.00032E-19	1.80509E-18
chr12	798374	802543	3.20474	1.1776E-239	5.5335E-238
chr12	814563	817972	2.42831	1.6106E-101	3.6593E-100
chr12	821313	824697	2.81335	3.6644E-151	1.1376E-149
chr12	835225	836728	1.47326	2.97783E-17	2.56035E-16
chr12	845538	848526	1.82861	1.90414E-38	2.35668E-37
chr12	851940	852479	1.55527	2.00032E-19	1.80509E-18
chr12	865393	870044	4.37291	0	0
chr12	880859	883641	2.3008	1.59001E-86	3.2144E-85
chr12	886464	891415	2.62691	4.3053E-130	1.1858E-128
chr12	894587	896984	2.46137	1.3836E-110	3.3651E-109
chr12	909172	911402	1.80807	5.19517E-40	6.57052E-39
chr12	927863	928713	1.35545	2.00507E-09	1.39888E-08
chr12	952528	953613	1.54644	2.01233E-20	1.85353E-19
chr12	971059	972594	1.72271	3.7914E-32	4.28549E-31
chr12	997446	998070	1.31467	8.5008E-09	5.80644E-08
chr12	998446	1001646	4.08787	0	0
chr12	1003843	1008584	2.46296	4.9545E-104	1.1455E-102
chr12	1038073	1040295	2.24025	7.4319E-79	1.40443E-77
chr12	1064202	1064696	1.27326	1.81301E-11	1.34605E-10
chr12	1072126	1072371	1.86772	3.42058E-73	6.14469E-72
chr13	3529	3937	1.6223	2.37958E-31	2.65766E-30

chr13	8258	9743	2.48443	3.013E-104	6.9823E-103
chr13	39126	40046	1.48818	8.70763E-17	7.40969E-16
chr13	48896	50909	2.06377	5.31251E-60	8.43723E-59
chr13	52996	56291	1.83671	4.61424E-41	5.91834E-40
chr13	57192	60878	2.15806	3.18346E-68	5.46512E-67
chr13	73300	76024	2.16133	1.00277E-72	1.79349E-71
chr13	83860	86367	2.35788	1.41906E-89	2.94307E-88
chr13	87226	87618	1.38788	3.72821E-11	2.7429E-10
chr13	107340	113323	2.63706	2.023E-126	5.445E-125
chr13	144018	145607	2.52251	2.2909E-114	5.7016E-113
chr13	147328	149832	1.77507	3.13906E-34	3.66016E-33
chr13	152426	153667	1.6833	4.42181E-29	4.75992E-28
chr13	161553	162520	1.83102	3.48177E-45	4.71303E-44
chr13	172983	174740	2.03569	1.80302E-59	2.84905E-58
chr13	194712	195495	1.55076	1.00115E-19	9.08657E-19
chr13	202339	203128	1.3721	4.02717E-10	2.87224E-09
chr13	211138	213030	2.72391	2.1232E-153	6.6834E-152
chr13	226837	227362	1.46746	6.32121E-15	5.14517E-14
chr13	233394	235283	1.43175	1.44977E-13	1.13972E-12
chr13	237499	240254	3.62674	2.023E-270	1.0839E-268
chr13	240502	240959	1.34483	4.55512E-09	3.14007E-08
chr13	249549	251934	2.99053	2.2856E-190	8.6896E-189
chr13	252455	252874	1.27961	8.8247E-07	5.58959E-06
chr13	254175	259422	12.3039	0	0
chr13	259760	262706	2.49595	4.1687E-120	1.0789E-118
chr13	263120	271025	16.9086	0	0
chr13	274190	280387	9.39151	0	0
chr13	292984	296743	5.15459	0	0
chr13	298511	301889	3.51017	5.0816E-251	2.5119E-249
chr13	315949	317999	1.72397	1.33229E-35	1.58416E-34
chr13	319123	319805	1.50641	5.26502E-17	4.50194E-16
chr13	326524	330090	2.12511	5.88166E-72	1.044E-70
chr13	330923	332893	2.27916	6.18159E-84	1.22208E-82
chr13	338792	341985	2.62597	6.6834E-128	1.8155E-126
chr13	356264	356846	1.42205	2.29562E-12	1.74783E-11
chr13	363023	364518	2.78749	1.0186E-142	3.02E-141
chr13	385957	386648	1.2678	2.54015E-06	1.57805E-05

chr13	394431	395415	1.4942	5.21435E-17	4.45862E-16
chr13	402929	405023	2.53392	1.0209E-117	2.6062E-116
chr13	414631	417513	3.82625	0	0
chr13	425849	427087	1.73074	5.93472E-33	6.79204E-32
chr13	441271	445634	3.02057	2.0464E-183	7.5336E-182
chr13	458835	460535	1.62853	6.10099E-24	6.00759E-23
chr13	467033	469137	4.34609	0	0
chr13	488356	491679	3.08519	4.7643E-270	2.5468E-268
chr13	498456	499417	2.38785	5.68591E-93	1.21116E-91
chr13	499883	500144	1.61521	4.35011E-23	4.21697E-22
chr13	507247	507761	1.47164	1.85823E-15	1.53179E-14
chr13	519193	521601	2.71273	2.5527E-145	7.6736E-144
chr13	531162	533601	1.50033	3.09742E-17	2.66195E-16
chr13	538024	539273	1.86668	1.7746E-45	2.41102E-44
chr13	549084	550143	2.54434	1.3305E-118	3.4119E-117
chr13	553210	555283	3.10302	1.0209E-192	3.9264E-191
chr13	564356	564668	1.30574	7.79974E-08	5.14731E-07
chr13	576720	580835	3.32138	1.1482E-274	6.2517E-273
chr13	634474	636253	1.9274	5.80764E-50	8.31955E-49
chr13	638978	642810	5.21784	0	0
chr13	662412	663069	1.31965	4.04101E-08	2.69519E-07
chr13	665068	666758	1.68515	1.00925E-27	1.06267E-26
chr13	668369	669065	1.74177	9.48855E-32	1.06635E-30
chr13	669402	671651	3.56482	0	0
chr13	676510	678444	1.78666	1.81761E-40	2.31153E-39
chr13	697491	699982	1.97399	1.62967E-58	2.55153E-57
chr13	711768	714858	1.68804	1.84629E-33	2.12814E-32
chr13	716967	719132	1.46674	4.00405E-16	3.35429E-15
chr13	747625	750070	2.23799	1.7342E-76	3.20996E-75
chr13	760783	766101	3.82209	0	0
chr13	775260	775833	1.30968	7.8233E-08	5.16262E-07
chr13	778384	780704	1.46477	7.8614E-16	6.53883E-15
chr13	799473	803621	1.5862	1.32739E-23	1.29867E-22
chr13	824133	828303	3.2278	9.6605E-234	4.4157E-232
chr13	855006	855956	1.59221	6.87068E-22	6.51028E-21
chr13	869694	869995	1.38217	5.93198E-11	4.33641E-10
chr13	885757	891368	5.21512	0	0

chr14	37526	39430	1.56777	1.34586E-20	1.24308E-19
chr14	41630	43137	1.79762	6.6896E-39	8.33297E-38
chr14	49330	49869	1.27935	6.52845E-07	4.15863E-06
chr14	69933	73473	3.39869	1.3614E-277	7.4817E-276
chr14	94154	96400	2.48391	3.6983E-105	8.6298E-104
chr14	121050	121421	1.25587	2.37263E-06	1.47652E-05
chr14	124220	128150	3.25884	3.3497E-252	1.6596E-250
chr14	153393	158905	2.5056	1.074E-120	2.7925E-119
chr14	166052	167025	1.3465	2.21993E-10	1.59511E-09
chr14	178876	180273	1.54284	4.58458E-21	4.27464E-20
chr14	194222	196903	2.42741	7.2611E-145	2.1777E-143
chr14	208687	209809	1.3755	3.83619E-11	2.82098E-10
chr14	213453	216305	2.11391	1.47979E-77	2.76503E-76
chr14	218592	219169	1.4872	3.95458E-16	3.31284E-15
chr14	221811	223727	1.52514	2.79898E-18	2.46434E-17
chr14	223978	224974	1.2864	4.1096E-07	2.63846E-06
chr14	253990	258162	3.13525	1.5066E-221	6.5464E-220
chr14	259616	262187	2.53385	3.069E-121	7.9983E-120
chr14	290605	293740	4.86497	0	0
chr14	313544	313803	1.28456	2.36423E-07	1.53218E-06
chr14	314122	317749	3.40693	1.8155E-283	1.0186E-281
chr14	318604	319429	1.2897	1.84421E-07	1.20022E-06
chr14	334122	335649	2.203	1.66763E-78	3.14196E-77
chr14	343110	344224	2.89453	1.2794E-175	4.529E-174
chr14	347275	347757	1.43204	7.67361E-13	5.91834E-12
chr14	357717	362703	3.02137	4.2756E-202	1.7061E-200
chr14	368198	368652	1.25113	4.0724E-06	2.50877E-05
chr14	388632	391086	3.1465	2.3388E-228	1.0471E-226
chr14	396244	399037	3.15983	7.4817E-209	3.0832E-207
chr14	401609	403024	1.83311	3.23668E-43	4.27071E-42
chr14	409046	411042	2.32693	3.72907E-94	8.01863E-93
chr14	420296	421472	1.78887	1.07349E-39	1.35114E-38
chr14	433532	434125	1.28928	2.67442E-07	1.7301E-06
chr14	434481	439327	2.0054	2.61758E-61	4.2092E-60
chr14	468543	470721	2.65677	1.1092E-134	3.1405E-133
chr14	473978	477443	2.79536	8.0724E-160	2.6303E-158
chr14	484743	487422	2.17623	7.07457E-75	1.29062E-73

chr14	492548	493800	2.26473	2.15625E-83	4.24229E-82
chr14	494559	495998	1.7873	3.67198E-36	4.40149E-35
chr14	504394	505764	1.95334	7.31139E-51	1.05779E-49
chr14	511140	511486	1.31138	5.97228E-08	3.95786E-07
chr14	511819	512379	2.03484	3.05351E-56	4.67412E-55
chr14	512634	513204	1.84168	1.97379E-39	2.47628E-38
chr14	532782	536210	1.77913	2.27143E-35	2.69215E-34
chr14	538757	540466	1.77633	5.24204E-37	6.3577E-36
chr14	556787	557743	1.31548	7.39776E-08	4.88832E-07
chr14	560936	561604	2.02818	1.27468E-55	1.93865E-54
chr14	568708	569713	1.25142	2.97413E-06	1.84281E-05
chr14	581662	584786	2.78592	6.1944E-160	2.0184E-158
chr14	587687	588756	1.40179	4.35712E-12	3.29155E-11
chr14	609775	618486	10.7051	0	0
chr14	619647	631183	14.8857	0	0
chr14	637273	650871	5.76604	0	0
chr14	653374	655012	2.46985	4.3451E-117	1.1041E-115
chr14	667392	667876	1.44668	3.26588E-14	2.61036E-13
chr14	671604	673701	1.34287	2.37017E-09	1.64926E-08
chr14	676230	681980	3.63895	2.8445E-275	1.5524E-273
chr14	710659	717129	2.635	3.4435E-130	9.506E-129
chr14	748202	750687	2.33973	1.2677E-104	2.9444E-103
chr14	751032	752307	2.09069	1.29599E-73	2.33668E-72
chr14	783399	783891	2.8098	4.6989E-233	2.1429E-231
chr15	1131	1929	1.91688	4.72498E-64	7.7983E-63
chr15	15148	15831	1.28218	1.14391E-06	7.21573E-06
chr15	22975	23334	1.62989	5.58856E-43	7.35191E-42
chr15	76599	79365	1.86249	5.626E-43	7.39946E-42
chr15	89215	90679	1.68358	5.44753E-28	5.7597E-27
chr15	112641	113432	1.55194	3.13329E-19	2.81579E-18
chr15	131243	135161	4.45463	0	0
chr15	148602	151605	2.91225	7.1121E-183	2.6062E-181
chr15	179787	180832	1.40688	1.85055E-12	1.41221E-11
chr15	187980	192806	4.95452	0	0
chr15	195482	200115	2.29562	1.58635E-95	3.44588E-94
chr15	200481	200875	1.35713	1.14304E-10	8.28343E-10
chr15	213119	214104	1.51118	2.17771E-17	1.87845E-16

chr15	223869	224725	1.31881	5.55022E-08	3.68383E-07
chr15	238031	240843	1.8153	2.43052E-42	3.17103E-41
chr15	247711	249043	1.71702	4.4157E-32	4.9877E-31
chr15	263148	267174	1.83666	2.52813E-42	3.29762E-41
chr15	269535	270571	1.49188	3.60911E-18	3.16811E-17
chr15	278976	283623	3.30085	4.3752E-253	2.1827E-251
chr15	293663	295499	1.96443	7.89587E-57	1.21535E-55
chr15	299956	301094	1.36544	7.70797E-10	5.44967E-09
chr15	306567	314033	5.02604	0	0
chr15	319346	338997	11.5127	0	0
chr15	340372	341485	1.93527	1.38931E-52	2.04786E-51
chr15	345593	346438	1.43416	8.5428E-19	7.60326E-18
chr15	353095	354468	1.95824	2.80931E-49	3.99485E-48
chr15	359852	360820	1.52882	1.07127E-18	9.517E-18
chr15	365718	367869	1.79567	8.7963E-38	1.07845E-36
chr15	369188	371056	1.85414	1.32221E-43	1.75348E-42
chr15	371545	376099	4.99487	0	0
chr15	388709	391247	1.31465	4.20979E-09	2.90489E-08
chr15	391662	394114	3.09294	1.1169E-210	4.6345E-209
chr15	412276	416491	2.61098	9.2683E-120	2.3933E-118
chr15	416772	417111	1.41816	2.22177E-12	1.692E-11
chr15	418251	419180	1.31664	3.2017E-08	2.1422E-07
chr15	432909	433719	1.52404	1.10943E-17	9.62942E-17
chr15	450346	453271	3.23376	4.6026E-207	1.8793E-205
chr15	454196	456332	3.2544	3.3806E-235	1.556E-233
chr15	456727	457016	1.36236	6.3866E-10	4.52585E-09
chr15	466185	466836	1.28218	1.14391E-06	7.21573E-06
chr15	468450	470236	2.50512	1.6558E-116	4.1879E-115
chr15	475926	477420	1.62755	3.004E-26	3.0839E-25
chr15	482095	485432	3.63278	0	0
chr15	488808	490228	1.56121	1.47096E-21	1.38484E-20
chr15	495853	499392	2.83068	8.6896E-157	2.7797E-155
chr15	507171	508086	1.42064	2.08689E-12	1.59074E-11
chr15	535866	536920	1.6645	1.89234E-28	2.01651E-27
chr15	537214	541567	3.35278	1.1695E-265	6.1376E-264
chr15	547459	549561	1.78949	7.36885E-40	9.29822E-39
chr15	551303	552232	1.65174	5.01303E-26	5.12625E-25

chr15	562758	565514	2.37866	2.208E-106	5.2E-105
chr15	580985	583449	3.86012	0	0
chr15	588940	591368	2.58488	5.4576E-132	1.5205E-130
chr15	608035	610549	1.73178	5.07341E-31	5.63638E-30
chr15	611924	613021	1.77448	2.24492E-34	2.62241E-33
chr15	624402	627396	1.94988	5.58856E-59	8.7882E-58
chr15	635527	637822	2.35395	9.9541E-103	2.2856E-101
chr15	646711	647526	1.38948	5.86678E-11	4.28894E-10
chr15	650394	652603	1.79831	4.51024E-36	5.40008E-35
chr15	657724	661140	2.8574	1.766E-174	6.2087E-173
chr15	663135	663688	1.29315	2.25907E-07	1.46599E-06
chr15	696206	699915	3.66027	0	0
chr15	700261	700579	1.4487	1.18059E-13	9.30251E-13
chr15	714657	716855	2.1253	5.24566E-66	8.8247E-65
chr15	717911	719051	2.06333	6.98715E-61	1.11892E-59
chr15	725441	726971	2.48064	4.5394E-107	1.074E-105
chr15	727708	730575	2.90331	4.4771E-168	1.5241E-166
chr15	731484	731758	1.27675	7.9006E-07	5.0151E-06
chr15	735026	739699	2.8529	4.4361E-175	1.5631E-173
chr15	751061	753207	2.46071	8.356E-117	2.1184E-115
chr15	765724	767593	2.1218	3.83972E-70	6.70656E-69
chr15	776747	778542	2.91454	6.7764E-192	2.5942E-190
chr15	778823	779618	2.22466	4.39036E-75	8.02232E-74
chr15	787325	791335	2.40635	1.4521E-102	3.3266E-101
chr15	792787	795894	2.83311	4.0087E-167	1.3583E-165
chr15	797754	799790	1.23494	1.03739E-06	6.5533E-06
chr15	804204	805434	1.55438	3.66522E-20	3.3566E-19
chr15	808012	808870	1.31014	2.77581E-08	1.86196E-07
chr15	815297	815776	1.3597	2.79679E-10	2.00433E-09
chr15	821046	823436	2.76944	7.8524E-145	2.355E-143
chr15	826960	828118	1.92595	1.05925E-50	1.52968E-49
chr15	849469	850064	2.00497	6.2087E-109	1.4928E-107
chr15	854685	856068	2.83363	1.6711E-149	5.1523E-148
chr15	873350	873743	1.32825	1.58972E-08	1.07565E-07
chr15	886809	891543	2.62332	3.6728E-143	1.0914E-141
chr15	912930	913986	1.69507	7.99098E-29	8.56643E-28
chr15	917631	919555	2.31019	1.52721E-96	3.34349E-95

chr15	934760	940572	4.44665	0	0
chr15	964231	967456	2.90072	3.2211E-158	1.0399E-156
chr15	982024	986487	2.73783	1.0666E-172	3.7154E-171
chr15	990228	993965	3.01897	1.2474E-187	4.6881E-186
chr15	1011297	1013890	2.59092	8.2985E-122	2.1677E-120
chr15	1085090	1085349	2.02672	2.1707E-93	4.63981E-92
chr16	37228	38010	1.38353	5.78496E-11	4.2297E-10
chr16	49940	50290	1.25887	6.70934E-06	4.09355E-05
chr16	50577	52657	3.5667	2.4774E-261	1.2794E-259
chr16	53200	53779	1.31931	3.33803E-08	2.23208E-07
chr16	69993	73178	2.29958	8.84097E-87	1.79184E-85
chr16	76407	77768	1.40661	6.56901E-13	5.07458E-12
chr16	88883	92397	3.27521	8.3368E-239	3.8905E-237
chr16	119500	121157	1.90828	6.05202E-45	8.16959E-44
chr16	123636	124194	1.39874	2.73149E-11	2.01739E-10
chr16	124535	127199	2.14141	1.50176E-66	2.53922E-65
chr16	137748	138874	1.5586	1.27438E-19	1.15478E-18
chr16	139360	140810	2.62529	3.062E-127	8.2794E-126
chr16	148063	149715	2.07638	2.85036E-68	4.89441E-67
chr16	161814	162370	1.30392	1.87392E-07	1.21933E-06
chr16	166230	169097	3.12409	1.2134E-193	4.6774E-192
chr16	178716	179555	1.39071	5.26381E-11	3.85478E-10
chr16	179832	185218	2.70845	2.4155E-136	6.9024E-135
chr16	191769	194656	1.53862	1.84629E-18	1.63155E-17
chr16	210579	211141	1.2622	5.25049E-06	3.21907E-05
chr16	212205	212731	1.40207	1.93108E-11	1.43245E-10
chr16	219200	221164	1.75794	1.93286E-36	2.32595E-35
chr16	232032	233662	1.70222	1.25199E-30	1.38197E-29
chr16	238474	239582	1.62401	1.11122E-24	1.10917E-23
chr16	249181	251789	4.03303	0	0
chr16	254827	255824	1.42973	3.5514E-13	2.76376E-12
chr16	274867	278326	4.9945	0	0
chr16	283088	284378	1.98155	2.34207E-51	3.40722E-50
chr16	315103	319541	2.56687	7.145E-127	1.932E-125
chr16	322798	324506	2.30579	7.56659E-88	1.54739E-86
chr16	330612	333425	3.83452	0	0
chr16	340784	342046	1.83907	1.48765E-45	2.02395E-44

chr16	394503	399659	5.22234	0	0
chr16	421728	423183	1.93574	2.59239E-53	3.84946E-52
chr16	423527	424023	1.30629	8.55638E-08	5.63923E-07
chr16	427760	428923	1.29651	2.26345E-07	1.46879E-06
chr16	435932	436774	1.67213	5.89522E-28	6.23017E-27
chr16	443249	446209	3.80725	0	2.138E-307
chr16	446649	447343	1.64381	1.24022E-27	1.30347E-26
chr16	469393	474741	2.44459	4.7863E-107	1.1324E-105
chr16	485840	486543	1.44748	7.60677E-14	6.02282E-13
chr16	486969	487715	1.29289	2.23224E-07	1.44874E-06
chr16	499006	501178	2.37084	3.55959E-91	7.47481E-90
chr16	508845	514754	3.01687	7.4473E-180	2.6915E-178
chr16	523696	525863	3.00973	2.0324E-182	7.4473E-181
chr16	529621	537868	5.07024	0	0
chr16	539440	544015	3.62233	4.3551E-301	2.6002E-299
chr16	544676	567612	10.3455	0	0
chr16	574470	575339	1.53336	2.7874E-20	2.55918E-19
chr16	576535	579113	2.31791	4.02161E-85	8.03526E-84
chr16	598799	602613	3.81968	0	0
chr16	618036	620685	3.14051	6.4565E-193	2.4889E-191
chr16	623484	625359	2.31335	2.48256E-92	5.26138E-91
chr16	635915	638612	2.70195	2.2131E-140	6.4714E-139
chr16	649206	652421	4.38272	0	0
chr16	660307	661545	1.58428	3.55795E-22	3.38844E-21
chr16	670598	670866	1.26472	2.16048E-06	1.34654E-05
chr16	673721	675535	1.3509	1.10202E-09	7.74997E-09
chr16	691346	692770	1.70088	8.21486E-31	9.09494E-30
chr16	702098	703379	1.81295	5.76634E-41	7.38414E-40
chr16	707470	709297	1.55855	4.9705E-23	4.81061E-22
chr16	710412	711858	1.93178	4.15623E-50	5.96211E-49
chr16	714117	717825	3.22693	3.4754E-241	1.6406E-239
chr16	732703	734290	2.34927	1.0375E-101	2.3621E-100
chr16	748838	749345	1.24958	5.25618E-06	3.22055E-05
chr16	749608	749874	1.26014	1.44797E-06	9.09432E-06
chr16	764944	769192	2.44312	1.7865E-105	4.1783E-104
chr16	773039	774394	1.55527	2.00032E-19	1.80509E-18
chr16	776220	777058	1.71377	6.5539E-30	7.15155E-29

chr16	779854	782233	1.62103	9.50824E-26	9.67609E-25
chr16	782567	784572	1.41247	8.66762E-13	6.67421E-12
chr16	790668	791518	1.24318	7.82258E-06	4.75718E-05
chr16	802349	804568	3.45899	6.8865E-250	3.3884E-248
chr16	810679	812245	1.57826	4.90682E-24	4.84061E-23
chr16	814441	816576	2.20212	3.53102E-79	6.69268E-78
chr16	835082	836584	2.03046	5.99929E-56	9.15588E-55
chr16	862549	865256	2.10744	5.86273E-69	1.01321E-67
chr16	875926	876998	1.72178	2.66624E-30	2.92887E-29
chr16	884898	887150	1.82169	7.74462E-38	9.50605E-37
chr16	890249	891199	1.57523	7.23936E-22	6.85646E-21
chr16	892788	894973	1.6423	4.0439E-30	4.42894E-29
chr16	896127	897666	1.4981	3.85567E-18	3.38298E-17
chr16	915272	918401	2.53613	1.3002E-118	3.3343E-117
chr16	923754	926086	1.28376	6.91035E-13	5.33581E-12
chr16	944203	944500	2.01353	1.5136E-150	4.6881E-149
chr16	945999	946627	2.18712	2.3714E-283	1.3305E-281
chr2	890	1149	2.52267	9.1411E-265	4.7863E-263
chr2	1485	1942	2.41546	6.8865E-286	3.8994E-284
chr2	5922	6239	1.99512	2.35939E-83	4.63981E-82
chr2	6766	7501	2.41422	5.3827E-291	3.1046E-289
chr2	43705	44538	1.39178	7.87771E-12	5.90881E-11
chr2	50284	52295	1.72851	4.28351E-33	4.91247E-32
chr2	65875	68126	2.43709	3.2211E-110	7.7983E-109
chr2	77520	80263	3.2599	3.0061E-213	1.2618E-211
chr2	93260	93932	1.38542	1.06864E-10	7.75443E-10
chr2	104668	106243	2.19056	1.4832E-73	2.67301E-72
chr2	112912	113307	1.34348	1.12689E-09	7.92246E-09
chr2	114835	116634	1.78089	2.36592E-37	2.88403E-36
chr2	117830	120493	2.34448	4.48436E-88	9.18544E-87
chr2	127039	128336	1.69544	6.61759E-31	7.33669E-30
chr2	148224	154644	5.19513	0	0
chr2	161937	162674	1.34116	6.63606E-09	4.55009E-08
chr2	172938	176344	4.60679	0	0
chr2	185124	186124	1.48593	3.89314E-17	3.33734E-16
chr2	186373	187590	1.45918	1.26386E-15	1.04616E-14
chr2	198535	199372	1.46002	9.66051E-15	7.82708E-14

chr2	205705	206609	1.5124	1.56063E-18	1.38166E-17
chr2	209450	214665	7.52031	0	0
chr2	230313	235370	4.50966	0	0
chr2	235784	240928	16.6632	0	0
chr2	241263	241556	1.37543	2.90061E-10	2.07793E-09
chr2	241969	242230	1.28551	8.7961E-07	5.57519E-06
chr2	242544	254002	8.00179	0	0
chr2	267081	268037	1.34645	1.20751E-09	8.48242E-09
chr2	279331	286326	4.31579	0	0
chr2	303568	305014	1.97202	6.68036E-54	9.977E-53
chr2	305281	305962	1.67516	4.89103E-27	5.09096E-26
chr2	318643	323918	4.51233	0	0
chr2	327142	328818	1.71221	2.07348E-30	2.28087E-29
chr2	333822	334818	2.22552	6.35185E-80	1.21171E-78
chr2	344677	345087	1.29161	4.89058E-07	3.13177E-06
chr2	348447	355294	2.56436	5.9841E-114	1.4894E-112
chr2	387836	390117	2.07516	3.19963E-65	5.34195E-64
chr2	390522	391229	1.40402	3.01162E-12	2.2856E-11
chr2	393565	397690	3.942	0	0
chr2	412224	414136	2.30598	6.55994E-91	1.37467E-89
chr2	441592	444777	2.18433	5.96211E-77	1.1079E-75
chr2	459317	463244	2.027	6.57809E-64	1.08443E-62
chr2	469518	471423	2.34399	3.4514E-162	1.1402E-160
chr2	479202	481577	1.94414	7.68953E-54	1.14789E-52
chr2	498607	500088	1.70774	2.26048E-31	2.52522E-30
chr2	511281	520032	4.17618	0	0
chr2	540069	542433	2.37272	2.4604E-109	5.9156E-108
chr2	549847	551277	1.57315	1.21395E-21	1.14472E-20
chr2	569394	571165	1.76136	3.84503E-34	4.4761E-33
chr2	572156	574638	2.91225	7.1121E-183	2.6062E-181
chr2	603102	604412	2.02909	1.73221E-60	2.76376E-59
chr2	607532	608118	1.37543	2.90061E-10	2.07793E-09
chr2	608463	611158	1.68848	5.94155E-28	6.27914E-27
chr2	631040	634101	1.98197	1.19454E-56	1.83612E-55
chr2	634752	641039	3.25487	1.7378E-282	9.7275E-281
chr2	653646	658712	3.05919	2.133E-209	8.8105E-208
chr2	658968	659272	1.55255	1.93152E-20	1.77951E-19

chr2	672538	677455	2.45646	2.3014E-111	5.6234E-110
chr2	689137	691179	1.75087	4.87192E-41	6.24597E-40
chr2	693264	697145	2.89708	2.9107E-166	9.8175E-165
chr2	704824	706028	1.40652	1.1272E-12	8.65167E-12
chr2	707259	709160	2.06754	1.45412E-64	2.41213E-63
chr2	731377	734246	2.55763	2.5527E-123	6.7453E-122
chr2	745147	746990	1.55581	5.14517E-21	4.79181E-20
chr2	749913	753552	3.04359	8.1658E-198	3.2063E-196
chr2	762141	763314	1.40044	3.90301E-13	3.03319E-12
chr2	801826	803293	1.95156	9.76338E-69	1.68384E-67
chr3	394	774	3.34699	1.0328E-224	4.5499E-223
chr3	1114	11061	3.91324	0	0
chr3	11315	11684	2.17778	4.29141E-90	8.93717E-89
chr3	13087	13565	1.63551	4.61424E-27	4.80397E-26
chr3	13943	16433	1.88794	6.65426E-46	9.09076E-45
chr3	26799	28058	1.80711	3.18273E-37	3.87168E-36
chr3	28622	30260	1.31593	2.83505E-08	1.90117E-07
chr3	33637	34313	1.34433	4.94015E-09	3.40173E-08
chr3	36518	37916	2.06047	9.31966E-70	1.62218E-68
chr3	38596	39132	1.35758	4.57762E-10	3.25829E-09
chr3	42232	46775	1.89419	3.16373E-49	4.49469E-48
chr3	54019	56772	2.95406	3.1915E-193	1.2274E-191
chr3	62863	67492	2.73207	2.421E-164	8.091E-163
chr3	74824	76071	1.24227	4.5848E-06	2.81722E-05
chr3	81534	82502	1.60296	1.46117E-23	1.42889E-22
chr3	83136	83963	2.33124	1.34927E-86	2.73086E-85
chr3	91007	94217	5.11245	0	0
chr3	97084	104116	10.873	0	0
chr3	104937	105549	1.26836	8.06418E-09	5.51214E-08
chr3	106658	107162	1.29952	6.5734E-08	4.35041E-07
chr3	107933	117496	15.2729	0	0
chr3	119006	119451	1.26196	7.68635E-07	4.8818E-06
chr3	128799	132128	8.36165	0	0
chr3	139114	139538	1.35681	7.87789E-10	5.56557E-09
chr3	143545	145727	2.19417	9.09494E-75	1.6573E-73
chr3	150924	151655	2.15473	6.9024E-68	1.18141E-66
chr3	157410	157856	1.28995	1.21065E-07	7.93634E-07

chr3	158148	158823	1.24554	8.20918E-06	4.98793E-05
chr3	188268	196813	5.15725	0	0
chr3	198760	199378	1.89295	7.09741E-79	1.34153E-77
chr3	200225	200734	1.69847	1.19757E-28	1.28027E-27
chr3	211813	212204	1.24639	5.86665E-08	3.88866E-07
chr3	212522	212804	1.29218	5.2689E-10	3.74421E-09
chr3	217160	222366	2.3848	1.7498E-121	4.5709E-120
chr3	230637	235465	3.10343	1.374E-291	7.9433E-290
chr3	243720	244002	1.22362	9.7717E-06	5.91548E-05
chr3	258313	260024	2.2224	5.49161E-85	1.09572E-83
chr3	275778	278648	1.94641	1.7108E-55	2.59836E-54
chr3	285609	289278	1.82673	1.58052E-49	2.25268E-48
chr3	289745	293066	2.6913	3.6983E-130	1.0186E-128
chr3	293804	294117	2.32791	3.15937E-86	6.37382E-85
chr3	294600	295506	1.94825	2.13108E-48	3.00054E-47
chr3	295794	298807	1.90203	9.24698E-47	1.27703E-45
chr3	299179	301651	1.87256	3.97375E-48	5.57828E-47
chr4	2127	2798	2.06912	8.05008E-60	1.27644E-58
chr4	24011	27513	4.6381	0	0
chr4	38359	39043	1.32773	1.14093E-09	8.0201E-09
chr4	47007	47537	1.52529	1.05124E-17	9.13482E-17
chr4	52761	55627	3.46971	4.9888E-265	2.6122E-263
chr4	91382	92985	2.23466	3.89852E-76	7.19283E-75
chr4	93256	93509	1.30216	2.27856E-07	1.47846E-06
chr4	95128	96910	2.0716	2.33453E-60	3.72049E-59
chr4	117023	118192	2.48776	1.205E-104	2.8054E-103
chr4	118560	119613	2.6566	6.6834E-128	1.8197E-126
chr4	142617	146931	4.28972	0	0
chr4	181084	181587	1.36487	3.77068E-10	2.69135E-09
chr4	187137	189154	1.66226	2.60016E-27	2.71894E-26
chr4	192171	196276	3.45987	2.9923E-273	1.6181E-271
chr4	201526	202625	1.34795	1.74566E-09	1.22E-08
chr4	203091	206548	4.71071	0	0
chr4	226337	226676	1.29455	8.62939E-08	5.6867E-07
chr4	226952	227286	1.67524	1.10255E-27	1.15958E-26
chr4	231509	232854	1.42707	5.45255E-13	4.22085E-12
chr4	234953	236635	2.62239	3.0061E-129	8.2414E-128

chr4	252539	254466	2.31323	1.3122E-89	2.72207E-88
chr4	255975	257219	1.75649	8.48985E-34	9.83558E-33
chr4	261639	264085	1.65109	1.77419E-28	1.89147E-27
chr4	279490	280477	1.83281	2.41213E-41	3.10599E-40
chr4	291377	294391	1.83404	5.09448E-42	6.61759E-41
chr4	297071	298943	2.12152	7.57182E-73	1.35581E-71
chr4	306503	306828	1.84501	1.0639E-39	1.33999E-38
chr4	310524	311401	1.45401	5.8776E-14	4.66659E-13
chr4	332860	333855	1.93083	6.86436E-50	9.82426E-49
chr4	335374	336965	1.63994	2.34207E-26	2.40824E-25
chr4	353416	356032	3.5556	1.3397E-269	7.145E-268
chr4	359082	359356	1.25887	6.70934E-06	4.09355E-05
chr4	372016	374242	1.8855	1.60435E-44	2.15328E-43
chr4	374488	380476	2.71298	1.6144E-152	5.0582E-151
chr4	381319	382061	1.27819	6.51823E-07	4.1526E-06
chr4	387939	389718	1.54944	3.77833E-19	3.38922E-18
chr4	398640	403020	6.77988	0	0
chr4	413204	415863	2.89073	7.925E-157	2.5351E-155
chr4	425670	426498	1.79838	5.15822E-36	6.17163E-35
chr4	430246	431538	1.82908	7.67538E-41	9.81522E-40
chr4	433259	434044	1.37015	1.89561E-10	1.36515E-09
chr4	435190	436061	1.76175	3.15283E-33	3.6241E-32
chr4	438962	450189	16.5721	0	0
chr4	450461	452593	6.11675	0	0
chr4	454545	458850	4.66963	0	0
chr4	468049	471820	4.6154	0	0
chr4	472920	481066	5.59038	0	0
chr4	489923	491942	2.41782	2.0361E-96	4.45451E-95
chr4	492325	495143	2.93501	1.7179E-179	6.1944E-178
chr4	508935	510416	1.95158	1.08643E-48	1.53462E-47
chr4	522319	522679	1.51863	2.47685E-17	2.13403E-16
chr4	554139	555622	2.0748	5.03617E-60	8.00203E-59
chr4	577790	578968	1.45869	3.73766E-14	2.98401E-13
chr4	580488	586000	3.84176	0	0
chr4	604793	609023	4.14223	0	0
chr4	611854	612382	1.36059	8.47227E-10	5.98026E-09
chr4	612919	614211	1.23512	7.0635E-06	4.30309E-05

chr4	623874	625004	1.52541	1.14156E-18	1.01368E-17
chr4	638570	642689	5.70646	0	0
chr4	659264	663130	2.90697	1.3062E-188	4.9317E-187
chr4	673026	673424	1.2493	5.10552E-06	3.13163E-05
chr4	677366	678468	1.92609	8.32147E-49	1.17679E-47
chr4	697148	701827	2.75642	5.236E-155	1.6596E-153
chr4	710477	715073	5.06476	0	0
chr4	732716	736036	2.29281	1.0814E-102	2.4774E-101
chr4	762478	765010	3.17399	7.3114E-213	3.062E-211
chr4	765886	767007	1.4165	6.88494E-13	5.31741E-12
chr4	777190	778662	1.58162	3.0839E-22	2.94036E-21
chr4	778921	783990	2.47396	8.1658E-114	2.0277E-112
chr4	784937	786686	1.6443	1.97651E-27	2.07062E-26
chr4	791105	792948	2.23906	2.23049E-84	4.4269E-83
chr4	824567	827218	2.67929	1.7498E-151	5.445E-150
chr4	827599	834050	2.05743	3.07044E-69	5.31986E-68
chr4	839313	840043	1.51156	3.25687E-19	2.92415E-18
chr4	840322	841303	2.04542	1.54597E-68	2.66073E-67
chr4	843215	844164	1.55593	1.41938E-20	1.31069E-19
chr4	863083	865557	3.24098	5.358E-297	3.155E-295
chr4	897724	902511	5.89801	0	0
chr4	920647	922756	1.99472	3.75751E-57	5.8023E-56
chr4	934026	935774	1.52447	1.64513E-19	1.48696E-18
chr4	936477	938210	2.96856	5.5335E-196	2.1528E-194
chr4	938594	939053	1.3406	3.16621E-10	2.26449E-09
chr4	962187	966313	2.57841	5.5081E-125	1.4723E-123
chr4	966694	968393	1.72765	6.87068E-32	7.73571E-31
chr4	993790	996370	1.90669	2.72772E-47	3.7914E-46
chr4	1022549	1022976	1.33908	5.26235E-09	3.61885E-08
chr4	1023687	1025201	2.08009	6.89922E-67	1.17004E-65
chr4	1025513	1026419	1.59943	7.09251E-23	6.84384E-22
chr4	1035223	1035681	1.3245	1.05626E-08	7.19035E-08
chr4	1041115	1046961	4.21636	0	0
chr4	1066020	1069985	3.24171	2.023E-216	8.5901E-215
chr4	1077182	1079132	1.74515	1.45144E-32	1.65158E-31
chr4	1088496	1089015	1.30549	4.48549E-08	2.98573E-07
chr4	1119154	1122220	2.92231	6.2373E-187	2.3335E-185

chr4	1143323	1148307	2.1466	2.02255E-74	3.67451E-73
chr4	1151729	1152942	1.54861	4.89666E-19	4.38329E-18
chr4	1153356	1154038	1.27885	1.48392E-06	9.31644E-06
chr4	1165361	1166059	1.28218	1.14391E-06	7.21573E-06
chr4	1166440	1167469	1.3734	1.35828E-10	9.82404E-10
chr4	1188396	1193012	4.83072	0	0
chr4	1214292	1215284	1.41965	5.3518E-13	4.14381E-12
chr4	1220452	1221074	1.38788	2.63998E-11	1.95065E-10
chr4	1238151	1238691	1.39018	6.09256E-11	4.45195E-10
chr4	1247500	1248900	1.78378	5.84655E-38	7.18787E-37
chr4	1253766	1256761	3.22701	1.6943E-234	7.7804E-233
chr4	1265754	1266339	1.36564	2.35288E-10	1.68943E-09
chr4	1271674	1273674	1.80544	1.13006E-41	1.46117E-40
chr4	1281856	1286647	4.78042	0	0
chr4	1311494	1312726	1.48109	5.665E-16	4.72825E-15
chr4	1313123	1316473	3.85791	0	0
chr4	1323587	1324285	1.482	2.36157E-15	1.94267E-14
chr4	1339996	1341269	1.4471	7.89042E-14	6.24597E-13
chr4	1343309	1345234	1.57251	4.19759E-21	3.91742E-20
chr4	1378278	1381416	4.04773	0	0
chr4	1393062	1394154	1.45622	3.971E-15	3.24788E-14
chr4	1396777	1400829	2.76196	5.105E-144	1.5241E-142
chr4	1403424	1404853	1.49025	4.40453E-16	3.68638E-15
chr4	1406298	1408779	1.89121	9.99079E-49	1.41189E-47
chr4	1413878	1415585	1.98272	4.55093E-54	6.80769E-53
chr4	1423570	1426099	2.87607	9.506E-169	3.2509E-167
chr4	1437444	1439230	2.2946	1.44677E-82	2.82488E-81
chr4	1449367	1450197	1.68178	1.21619E-27	1.2785E-26
chr4	1451455	1454000	2.51316	5.9841E-116	1.5066E-114
chr4	1454444	1457262	2.23133	8.75185E-76	1.60953E-74
chr4	1480751	1481543	1.28238	7.89987E-07	5.01464E-06
chr4	1523596	1523867	1.83502	6.75616E-39	8.41589E-38
chr4	1524253	1525052	3.17898	0	0
chr5	363	996	2.8108	7.3282E-146	2.2131E-144
chr5	1329	2157	2.56288	0	0
chr5	28427	31194	2.40539	2.8655E-98	6.35331E-97
chr5	35180	37077	2.23598	1.02235E-81	1.98061E-80

chr5	77851	79494	3.31648	1.7824E-236	8.2414E-235
chr5	93757	98006	2.49573	5.4075E-112	1.3274E-110
chr5	101506	103772	3.17381	6.2951E-198	2.4717E-196
chr5	114491	117333	4.16001	0	0
chr5	118405	119559	1.67922	7.89223E-34	9.14745E-33
chr5	133893	135581	3.94645	0	0
chr5	135950	139243	5.10925	0	0
chr5	143050	159024	17.5752	0	0
chr5	160593	169705	5.71629	0	0
chr5	185609	188255	3.90917	0	0
chr5	188577	190567	3.92618	0	0
chr5	205141	206209	1.43204	7.67361E-13	5.91834E-12
chr5	206716	207616	3.00729	2.4717E-173	8.6497E-172
chr5	215760	219822	5.78593	0	0
chr5	231874	236361	4.04273	0	0
chr5	243054	244546	1.74601	2.49172E-32	2.82488E-31
chr5	254977	258033	3.14384	2.0464E-193	7.8886E-192
chr5	272914	276017	2.76887	6.5615E-149	2.0137E-147
chr5	300915	302329	1.61998	1.82138E-24	1.81092E-23
chr5	305229	305679	1.3288	2.30978E-08	1.55418E-07
chr5	306043	306710	1.41206	6.72667E-12	5.05592E-11
chr5	307325	310446	2.82643	1.8493E-165	6.2087E-164
chr5	323533	325345	1.92599	3.75059E-52	5.50174E-51
chr5	331084	333523	2.38066	4.6666E-104	1.0814E-102
chr5	338910	339640	1.6685	1.38676E-26	1.43219E-25
chr5	357298	358637	1.59425	3.0761E-22	2.93292E-21
chr5	360150	360854	1.37593	1.1382E-10	8.24879E-10
chr5	365343	370172	3.44547	1.574E-279	8.7096E-278
chr5	372673	374333	1.83917	6.95184E-41	8.89406E-40
chr5	378349	379234	1.27329	2.05461E-06	1.28153E-05
chr5	412679	413291	1.34213	6.93745E-09	4.75401E-08
chr5	418848	422049	4.73588	0	0
chr5	424439	426361	2.03373	2.84643E-66	4.79954E-65
chr5	449770	450782	1.8883	2.93765E-43	3.87882E-42
chr5	454511	455492	1.27654	6.95392E-07	4.42283E-06
chr5	456579	459244	2.76443	3.155E-158	1.0186E-156
chr5	465937	466848	1.73163	1.67071E-33	1.92752E-32

chr5	475560	477326	1.87224	1.41612E-54	2.1301E-53
chr5	519623	522074	2.60842	3.7931E-120	9.8175E-119
chr5	527967	532027	3.80787	0	0
chr5	554149	557260	1.58141	3.50913E-23	3.40643E-22
chr5	574922	575350	2.24204	6.8391E-164	2.2803E-162
chr6	936	1274	2.14824	0	0
chr6	32623	33328	1.32606	8.61609E-09	5.8841E-08
chr6	38471	39198	1.30504	2.0807E-08	1.40197E-07
chr6	41926	43604	1.5902	2.4632E-23	2.39883E-22
chr6	62109	64020	2.13061	1.45747E-73	2.62664E-72
chr6	66459	71578	5.71959	0	0
chr6	77646	79210	1.68505	3.46657E-36	4.15623E-35
chr6	79763	80172	1.30745	1.20659E-08	8.19917E-08
chr6	82083	87542	2.53735	2.8576E-135	8.1283E-134
chr6	92722	93484	1.46481	9.66051E-17	8.20729E-16
chr6	97461	99252	1.75298	2.15675E-37	2.63027E-36
chr6	99533	101348	1.52691	3.70681E-18	3.25387E-17
chr6	128597	136610	9.37792	0	0
chr6	143916	162218	12.9751	0	0
chr6	164487	169428	4.60253	0	0
chr6	169725	176187	7.61725	0	0
chr6	189770	191750	1.8271	3.56533E-41	4.57931E-40
chr6	193031	194782	1.95264	1.48252E-52	2.18374E-51
chr6	195357	202717	2.89192	1.2677E-179	4.5709E-178
chr6	214139	218946	4.79342	0	0
chr6	231129	232495	1.5303	1.2331E-19	1.11764E-18
chr6	236651	240448	2.61695	9.7051E-122	2.5351E-120
chr6	248365	248959	1.60093	2.26934E-23	2.21055E-22
chr7	514	4621	3.19291	0	0
chr7	4868	6166	1.3191	2.91595E-09	2.02269E-08
chr7	7499	7955	1.29873	6.37236E-12	4.79292E-11
chr7	25423	26542	1.66517	2.32863E-26	2.39552E-25
chr7	51900	52815	1.88592	5.43E-47	7.52142E-46
chr7	61300	62394	1.39976	1.27292E-11	9.49074E-11
chr7	62737	64746	1.44919	6.85015E-14	5.43E-13
chr7	75094	75363	1.28551	8.7961E-07	5.57519E-06
chr7	89935	90510	1.29032	4.0577E-07	2.60561E-06

chr7	100805	104212	4.66996	0	0
chr7	110692	111163	1.53435	1.44311E-26	1.48936E-25
chr7	111607	112619	1.38463	9.88553E-11	7.18042E-10
chr7	116729	118349	1.85408	2.06348E-41	2.65889E-40
chr7	137032	140584	3.09722	7.709E-216	3.2659E-214
chr7	144827	146136	2.14474	6.96947E-67	1.18195E-65
chr7	162813	164093	1.71687	1.21367E-31	1.36144E-30
chr7	169210	173623	6.19423	0	0
chr7	174560	176177	1.93802	3.74887E-51	5.44127E-50
chr7	181708	182668	2.85645	2.1727E-170	7.4817E-169
chr7	186589	187259	1.40043	1.32801E-11	9.89692E-11
chr7	187543	187862	1.41626	2.24543E-12	1.71002E-11
chr7	218922	222701	3.83812	0	0
chr7	231796	233243	1.59917	8.18088E-26	8.33489E-25
chr7	240185	242310	2.57332	2.851E-134	8.0353E-133
chr7	252490	253009	1.3476	2.23872E-11	1.65733E-10
chr7	258683	263236	3.12255	1.9953E-224	8.77E-223
chr7	273755	275752	1.78765	5.58727E-37	6.7733E-36
chr7	296545	299481	2.896	2.2029E-172	7.656E-171
chr7	300105	302015	1.58165	1.96426E-23	1.91558E-22
chr7	327533	327962	1.32674	2.70957E-08	1.81811E-07
chr7	328364	329809	1.61521	4.35011E-23	4.21697E-22
chr7	340768	342693	2.04913	1.47877E-63	2.42885E-62
chr7	348626	351422	2.20135	1.1844E-72	2.1169E-71
chr7	359460	360272	1.42921	4.44324E-13	3.44826E-12
chr7	360628	362518	1.54473	1.41091E-19	1.27673E-18
chr7	379029	380703	1.58719	3.69913E-26	3.79228E-25
chr7	388187	396867	2.38291	2.208E-106	5.2E-105
chr7	400205	401007	1.54528	7.63836E-19	6.80769E-18
chr7	405684	408280	2.4344	3.5359E-100	7.9488E-99
chr7	424684	425681	1.35539	1.8161E-10	1.30849E-09
chr7	430527	432972	3.69838	1.3996E-305	8.4723E-304
chr7	436619	437361	2.09145	1.24853E-61	2.01419E-60
chr7	439766	442581	1.2936	8.81495E-09	6.01825E-08
chr7	443285	444681	1.3188	1.68457E-09	1.1778E-08
chr7	456707	458568	1.40381	9.59401E-14	7.5753E-13
chr7	463371	464289	1.26329	6.07239E-07	3.87311E-06

chr7	466059	471249	6.93806	0	0
chr7	472973	474803	2.80953	5.4828E-173	1.9143E-171
chr7	475423	477867	2.59307	5.4702E-122	1.4355E-120
chr7	486866	499918	11.9625	0	0
chr7	500445	510600	7.70551	0	0
chr7	518969	522478	4.29614	0	0
chr7	530154	534069	5.0856	0	0
chr7	534524	534982	1.58857	2.00586E-21	1.88408E-20
chr7	541961	544800	2.49442	1.9231E-105	4.4978E-104
chr7	551705	555268	5.48337	0	0
chr7	556836	557326	1.32547	3.10127E-08	2.07711E-07
chr7	576592	577637	1.25918	1.1145E-06	7.03283E-06
chr7	585082	588388	3.91023	0	0
chr7	603218	605232	2.66562	4.3451E-138	1.2531E-136
chr7	611220	614740	5.23225	0	0
chr7	615074	615321	1.25887	6.70934E-06	4.09355E-05
chr7	629396	633098	3.58106	6.1376E-297	3.6141E-295
chr7	674647	676229	1.94218	3.04579E-48	4.28055E-47
chr7	680345	684870	5.14969	0	0
chr7	696721	698098	1.36483	4.32902E-10	3.08326E-09
chr7	723013	726489	4.33889	0	0
chr7	727873	728612	1.45832	4.67305E-16	3.90751E-15
chr7	744242	745006	1.42525	8.62581E-13	6.64202E-12
chr7	745380	746138	1.46546	7.23602E-15	5.88166E-14
chr7	747027	750058	2.49196	6.4121E-116	1.6181E-114
chr7	750406	751671	1.84867	6.60237E-43	8.67761E-42
chr7	762955	763882	1.58648	8.58816E-26	8.74581E-25
chr7	777417	778715	1.99973	3.74111E-55	5.66109E-54
chr7	783453	783977	1.2649	2.31153E-06	1.43913E-05
chr7	789937	792605	2.13898	1.25922E-73	2.27039E-72
chr7	804064	805988	1.85983	9.66941E-47	1.33506E-45
chr7	833163	833937	1.64778	1.73061E-25	1.75348E-24
chr7	839825	842778	1.68718	6.55994E-30	7.15814E-29
chr7	843927	845321	1.91494	1.63606E-45	2.22485E-44
chr7	845742	847483	1.46935	1.29509E-15	1.07177E-14
chr7	849015	851582	1.85931	1.44577E-44	1.94178E-43
chr7	858169	858658	1.5153	3.78879E-17	3.25087E-16

chr7	859526	860716	1.60988	2.74094E-23	2.66563E-22
chr7	869506	870768	1.46274	1.54241E-15	1.27438E-14
chr7	899035	901059	1.7358	2.27039E-34	2.65216E-33
chr7	922562	923100	1.25816	2.399E-06	1.49269E-05
chr7	934720	938974	3.64235	5.9566E-286	3.3806E-284
chr7	940131	941638	1.81863	8.02971E-41	1.0266E-39
chr7	946617	950848	4.84564	0	0
chr7	954254	955744	1.91777	4.7863E-47	6.63285E-46
chr7	960539	961996	1.7815	9.40156E-36	1.12021E-34
chr7	962460	963766	1.85704	2.1707E-41	2.7964E-40
chr7	990570	991750	1.67588	3.99301E-27	4.16102E-26
chr7	1005671	1008281	1.66953	5.53988E-29	5.95525E-28
chr7	1013536	1015404	2.03083	2.52813E-56	3.87258E-55
chr7	1018969	1019908	1.43947	3.2719E-14	2.61517E-13
chr7	1036462	1038185	1.72778	2.10814E-31	2.35613E-30
chr7	1038529	1040676	2.03968	1.08618E-58	1.70294E-57
chr7	1056165	1058079	2.50144	6.4121E-112	1.574E-110
chr7	1066762	1067602	1.21522	2.42019E-08	1.62645E-07
chr7	1083388	1084240	2.75559	3.3884E-214	1.4256E-212
chr7	1088989	1089437	2.16245	8.0168E-158	2.5823E-156
chr8	696	1090	2.72755	9.3972E-135	2.6669E-133
chr8	1690	2137	1.76818	2.0701E-161	6.8077E-160
chr8	2582	2875	1.78415	6.73442E-46	9.20026E-45
chr8	3559	3979	1.56526	5.14044E-20	4.6957E-19
chr8	27648	28599	1.33504	4.39633E-09	3.03194E-08
chr8	44400	47102	3.29588	1.0209E-221	4.4361E-220
chr8	58631	63045	9.2953	0	0
chr8	69625	74607	6.13781	0	0
chr8	75772	80780	4.19679	0	0
chr8	81459	83122	2.14141	1.50176E-66	2.53922E-65
chr8	92348	93023	3.03061	1.0495E-176	3.7325E-175
chr8	95231	95495	1.42538	1.59661E-12	1.22096E-11
chr8	95923	96230	1.33308	8.74802E-10	6.17234E-09
chr8	99860	101211	2.41121	4.60681E-97	1.01251E-95
chr8	103631	114415	12.6362	0	0
chr8	117742	121882	6.48636	0	0
chr8	123377	124731	1.74285	1.39798E-34	1.63832E-33

chr8	134232	140437	4.47843	0	0
chr8	140775	141843	1.39258	2.79062E-13	2.17671E-12
chr8	142120	142845	1.39876	7.01617E-13	5.41751E-12
chr8	154258	160919	5.30095	0	0
chr8	161247	161983	3.54348	1.6368E-256	8.2794E-255
chr8	162321	162641	1.39874	2.73149E-11	2.01739E-10
chr8	183018	185945	2.92273	3.0761E-177	1.099E-175
chr8	192622	192981	1.51863	2.47685E-17	2.13403E-16
chr8	195954	199625	2.43649	3.1696E-103	7.2946E-102
chr8	210691	213425	2.30417	4.28351E-84	8.48008E-83
chr8	214519	215408	1.74843	3.07326E-32	3.48017E-31
chr8	246619	250150	3.46601	1.1117E-288	6.368E-287
chr8	250632	253826	2.37597	3.9628E-111	9.6605E-110
chr8	256945	259262	2.4857	6.9343E-134	1.9543E-132
chr8	312645	313284	1.35972	1.27005E-10	9.19285E-10
chr8	313595	314710	1.90259	5.10623E-47	7.07457E-46
chr8	322695	327855	1.81105	2.30091E-48	3.23743E-47
chr8	329252	330511	1.33825	2.36205E-10	1.69594E-09
chr8	334461	335169	1.3712	9.06985E-11	6.59295E-10
chr8	338878	340257	1.41207	1.77052E-13	1.38835E-12
chr8	347685	351858	1.95157	3.04018E-58	4.7457E-57
chr8	352128	352828	2.16472	6.74062E-69	1.16439E-67
chr8	353099	354974	1.41821	3.95003E-13	3.06902E-12
chr8	361262	366253	2.24282	1.34617E-97	2.97098E-96
chr8	391993	393165	2.21467	4.87753E-74	8.8308E-73
chr8	393494	394821	1.64317	3.90301E-27	4.06818E-26
chr8	398356	399918	1.84633	4.59833E-41	5.89794E-40
chr8	408740	412175	2.91369	1.1015E-180	3.9994E-179
chr8	412954	415089	1.75897	9.95405E-38	1.21955E-36
chr8	423714	425576	1.65645	2.17771E-34	2.54449E-33
chr8	463257	464400	1.44898	1.57652E-14	1.27057E-13
chr8	472519	478247	2.55826	1.574E-125	4.217E-124
chr8	513587	516142	2.62574	2.0654E-135	5.8749E-134
chr8	518639	520562	1.7451	5.40505E-32	6.09818E-31
chr8	520915	523355	1.98307	1.62517E-53	2.41769E-52
chr8	554419	555318	1.80387	4.4361E-231	2.0045E-229
chr8	555646	556253	1.63847	4.3752E-190	1.6596E-188

chr8	559431	559675	2.27983	1.1735E-99	2.62845E-98
chr8	560458	560907	3.23449	5.236E-234	2.3933E-232
chr9	43	2553	3.22562	0	0
chr9	7530	8548	1.50084	2.88868E-72	5.1428E-71
chr9	14197	15896	1.57982	1.66878E-54	2.50842E-53
chr9	16186	17272	1.33427	4.24033E-20	3.87882E-19
chr9	49478	52938	3.41023	9.7949E-278	5.3827E-276
chr9	56637	58148	1.31429	4.50381E-09	3.10506E-08
chr9	79820	80765	2.50441	1.2134E-106	2.8642E-105
chr9	81052	81617	1.60159	3.111E-22	2.96551E-21
chr9	87053	88245	1.50402	1.24537E-18	1.10433E-17
chr9	104510	108091	2.93193	4.8978E-173	1.71E-171
chr9	125246	126630	1.92972	1.93508E-47	2.69401E-46
chr9	134824	135723	1.33791	3.59642E-09	2.488E-08
chr9	143029	145876	2.87722	2.4378E-165	8.1846E-164
chr9	154327	158490	3.04619	1.0965E-187	4.121E-186
chr9	162228	163729	1.93212	2.31206E-49	3.29003E-48
chr9	185848	187106	1.47524	4.06256E-17	3.48177E-16
chr9	190687	191587	1.3288	2.30978E-08	1.55418E-07
chr9	194956	196390	1.62853	6.10099E-24	6.00759E-23
chr9	205554	206150	1.58867	1.75186E-22	1.6788E-21
chr9	214254	214495	1.53195	4.42385E-18	3.87704E-17
chr9	214738	215140	1.482	2.36157E-15	1.94267E-14
chr9	216376	218400	2.8698	8.6298E-171	2.9854E-169
chr9	228185	231313	2.38374	3.4594E-101	7.8361E-100
chr9	232189	234310	1.31442	1.42141E-08	9.6323E-08
chr9	235243	235851	1.2752	6.12139E-07	3.90328E-06
chr9	251293	252559	2.04638	9.9977E-63	1.62892E-61
chr9	263440	266357	4.36887	0	0
chr9	278508	281188	2.37683	1.7426E-100	3.9283E-99
chr9	287265	288164	1.31816	4.69699E-08	3.12457E-07
chr9	294343	298336	6.8966	0	0
chr9	302015	305153	2.09875	8.78416E-67	1.48799E-65
chr9	313389	314920	1.43874	4.75773E-14	3.78704E-13
chr9	316669	317148	1.86499	2.51478E-41	3.23743E-40
chr9	317976	319627	3.30604	1.1588E-235	5.3456E-234
chr9	326188	331978	5.09056	0	0

chr9	334717	336906	2.92029	4.8641E-164	1.6218E-162
chr9	337823	339235	1.36146	6.04157E-10	4.2841E-09
chr9	339539	359653	14.7292	0	0
chr9	361424	367904	5.66835	0	0
chr9	372294	372923	1.83831	2.70271E-39	3.38376E-38
chr9	373218	373819	1.42883	7.31644E-13	5.64547E-12
chr9	375858	380512	3.65393	7.4131E-287	4.217E-285
chr9	385015	386496	1.88346	1.55991E-44	2.09411E-43
chr9	389089	390869	2.38886	8.5704E-143	2.541E-141
chr9	418895	420870	2.07859	3.22478E-66	5.43501E-65
chr9	432420	432665	1.26916	5.07984E-08	3.37567E-07

Rec8 peak locations at the 6h time point in the rapamycin-treated sample.

Chromosome	Start	End	Fold enrichment	p value	q value
chr1	181	1394	1.77159	1.4757E-131	8.0353E-130
chr1	10349	10676	1.27247	1.68962E-06	1.44754E-05
chr1	10923	11325	1.32193	1.56769E-08	1.50293E-07
chr1	11720	12118	1.3506	6.59933E-14	7.85416E-13
chr1	27227	27582	1.2237	3.61077E-12	4.04669E-11
chr1	40086	42885	3.72891	0	0
chr1	50613	53170	2.73496	1.5668E-135	8.7902E-134
chr1	53417	53900	1.26764	4.30467E-06	3.59741E-05
chr1	64821	66504	1.93431	5.65979E-51	1.44212E-49
chr1	67127	67565	1.39469	1.25684E-10	1.32568E-09
chr1	78265	84450	5.9618	0	0
chr1	87900	88870	1.51106	1.89496E-17	2.5229E-16
chr1	92939	98608	3.4921	7.9616E-274	9.8175E-272
chr1	122917	129706	5.13056	0	0
chr1	129953	130871	2.02871	3.35197E-51	8.56841E-50
chr1	131117	139374	6.33225	0	0
chr1	143724	160190	11.8869	0	0
chr1	166224	178818	5.17438	0	0
chr1	194991	196596	1.52056	5.14636E-17	6.76239E-16
chr1	196935	197295	1.59751	1.86767E-20	2.70707E-19
chr1	226318	226796	1.31461	1.10281E-15	1.39091E-14

chr1	228101	228377	2.84724	7.9616E-160	5.188E-158
chr10	71	3184	2.71883	0	0
chr10	7518	8080	1.31915	1.31099E-27	2.24078E-26
chr10	9089	9565	1.32894	2.60256E-14	3.13906E-13
chr10	14494	16271	1.88678	3.3113E-116	1.6069E-114
chr10	16689	17332	1.43871	2.36048E-26	3.92735E-25
chr10	27128	28519	1.72812	6.11646E-61	1.77623E-59
chr10	39178	41481	1.57308	3.39078E-23	5.25654E-22
chr10	66614	68002	1.43739	1.15931E-14	1.41482E-13
chr10	86762	87764	1.60136	5.16654E-22	7.79112E-21
chr10	91798	92086	1.33879	1.01922E-08	9.86552E-08
chr10	98847	99091	1.51929	2.76503E-16	3.55386E-15
chr10	99479	100668	1.40486	6.13338E-12	6.81397E-11
chr10	106918	107596	1.46335	2.13403E-15	2.66747E-14
chr10	121816	122269	1.28826	6.40973E-07	5.62898E-06
chr10	124018	127801	2.09391	4.37724E-67	1.36395E-65
chr10	147038	148421	1.84469	6.7936E-41	1.48594E-39
chr10	162159	166277	3.16377	1.0544E-201	8.8716E-200
chr10	166806	168680	1.78699	1.57109E-35	3.12824E-34
chr10	168943	170091	1.54924	1.06218E-18	1.46589E-17
chr10	170444	170705	1.5488	1.28027E-17	1.71317E-16
chr10	171072	171529	1.37474	1.31435E-09	1.32565E-08
chr10	209389	211043	2.28731	2.99089E-85	1.12047E-83
chr10	233993	234785	1.72055	3.34503E-29	5.91017E-28
chr10	235034	235624	1.35093	1.35719E-09	1.36808E-08
chr10	249196	253591	3.28086	1.1402E-216	1.052E-214
chr10	268861	276018	3.14076	8.3368E-204	7.1285E-202
chr10	283430	284580	1.46493	1.67494E-14	2.03283E-13
chr10	297586	302632	3.06324	2.4831E-193	1.9861E-191
chr10	304679	305775	1.2642	3.08361E-06	2.60052E-05
chr10	306329	309502	1.76751	9.32395E-38	1.93241E-36
chr10	317685	318318	1.40167	8.23759E-12	9.10961E-11
chr10	323779	325030	1.59999	8.03711E-23	1.23538E-21
chr10	325275	328855	1.8888	5.83445E-46	1.38166E-44
chr10	344484	344995	1.29373	4.61838E-07	4.08856E-06
chr10	346557	347957	1.51469	1.89933E-18	2.60256E-17
chr10	366374	372200	5.5252	0	0

chr10	383706	387109	2.01129	1.74462E-57	4.854E-56
chr10	387906	389064	1.34225	8.41415E-09	8.1758E-08
chr10	395163	396034	1.28287	5.33789E-07	4.70934E-06
chr10	397971	400026	1.50603	2.08065E-17	2.7663E-16
chr10	406174	407369	1.3934	1.9485E-11	2.12393E-10
chr10	407701	412470	5.65313	0	0
chr10	417334	418744	1.4201	4.80729E-12	5.36167E-11
chr10	419221	419982	1.33933	6.54983E-09	6.39838E-08
chr10	429165	444740	8.60216	0	0
chr10	446983	448410	1.45161	1.86638E-14	2.26152E-13
chr10	450352	451004	1.24521	9.00554E-06	7.36818E-05
chr10	451602	453621	3.49025	3.1477E-227	3.0974E-225
chr10	454830	455120	1.36307	2.50092E-09	2.49132E-08
chr10	456305	456552	1.34282	6.05006E-09	5.91902E-08
chr10	457156	459522	2.03375	2.94917E-56	8.07607E-55
chr10	459809	465500	3.74725	2.9923E-305	4.217E-303
chr10	465970	472346	2.1959	2.59179E-71	8.45084E-70
chr10	484086	485768	1.6548	1.99343E-26	3.322E-25
chr10	486601	487316	1.36146	6.89541E-10	7.04109E-09
chr10	499764	501272	2.14502	3.34195E-68	1.05463E-66
chr10	508965	511564	1.60951	3.81241E-25	6.18016E-24
chr10	515706	516841	1.49337	2.96825E-16	3.81154E-15
chr10	557630	558021	1.27149	5.36661E-06	4.45677E-05
chr10	564826	565290	1.26611	4.05098E-06	3.39086E-05
chr10	565570	566150	1.48494	3.52858E-16	4.52168E-15
chr10	566524	566783	1.39525	1.80323E-10	1.88899E-09
chr10	567065	569669	1.93461	1.10408E-49	2.76121E-48
chr10	569932	570653	1.4653	1.71633E-13	2.01558E-12
chr10	573967	576054	1.83141	4.18119E-36	8.41201E-35
chr10	589126	591673	3.69347	0	0
chr10	591961	592440	1.29987	1.94133E-07	1.75582E-06
chr10	602765	603106	1.27488	9.59025E-07	8.337E-06
chr10	628589	629526	1.48723	4.22669E-16	5.40132E-15
chr10	652747	655566	1.77657	2.53454E-37	5.21315E-36
chr10	655854	657339	1.60931	5.05941E-22	7.63133E-21
chr10	688910	689732	1.59289	5.26138E-22	7.93049E-21
chr10	711824	713160	1.34667	5.18084E-12	5.77298E-11

chr11	23966	25679	1.41818	2.83857E-13	3.30902E-12
chr11	25935	26388	1.38799	2.08478E-10	2.17821E-09
chr11	26757	27432	1.37356	1.16536E-10	1.23052E-09
chr11	41989	44103	2.73241	3.1696E-138	1.8113E-136
chr11	54179	58066	3.51362	1.4191E-275	1.762E-273
chr11	85652	86396	1.43982	1.95254E-14	2.36429E-13
chr11	105784	106508	1.39974	1.53699E-10	1.61536E-09
chr11	106804	107684	2.53279	2.5177E-107	1.1402E-105
chr11	108040	109143	1.68332	1.84374E-25	3.00954E-24
chr11	110265	111411	2.07452	1.10815E-56	3.05141E-55
chr11	129424	132518	2.4296	1.4223E-106	6.3973E-105
chr11	159440	160144	1.51761	2.7574E-17	3.65342E-16
chr11	182072	182356	1.30794	1.39306E-07	1.27011E-06
chr11	183874	188243	2.15372	1.24251E-69	3.98291E-68
chr11	221065	222302	1.92768	6.31393E-47	1.5167E-45
chr11	234367	240894	3.11574	6.4269E-187	4.9545E-185
chr11	251577	254493	3.20378	6.1944E-210	5.5081E-208
chr11	264482	267371	2.2239	8.94746E-81	3.20922E-79
chr11	292198	293257	1.46655	1.85695E-15	2.32541E-14
chr11	311547	311799	1.34235	2.30245E-08	2.18862E-07
chr11	324263	324860	1.67827	1.31917E-26	2.20648E-25
chr11	325105	326346	2.3077	7.02749E-88	2.70085E-86
chr11	328120	328721	1.40975	9.22571E-12	1.0181E-10
chr11	330039	332422	2.08132	6.53131E-68	2.05353E-66
chr11	335878	336266	1.3512	2.95563E-09	2.93515E-08
chr11	375538	380289	3.48846	3.0409E-252	3.4041E-250
chr11	389529	390232	1.47115	1.60066E-15	2.00863E-14
chr11	410987	416755	4.02046	0	0
chr11	418343	423543	4.16274	0	0
chr11	432083	432387	1.37065	2.27982E-09	2.27552E-08
chr11	432813	434335	1.9606	1.97788E-49	4.92833E-48
chr11	435709	443200	12.4864	0	0
chr11	443548	449575	5.47233	0	0
chr11	452468	456120	9.86212	0	0
chr11	470711	473814	2.70972	1.4421E-137	8.2224E-136
chr11	481035	482287	1.41752	1.0266E-12	1.17382E-11
chr11	484338	486308	1.55639	3.17541E-20	4.57509E-19

chr11	495273	499741	4.57244	0	0
chr11	500598	502095	1.5398	2.27877E-20	3.29534E-19
chr11	520513	523908	1.90751	9.52138E-45	2.2136E-43
chr11	524286	526377	1.36396	6.66423E-10	6.80926E-09
chr11	527030	535658	3.2613	7.925E-211	7.0795E-209
chr11	535950	537423	1.54193	3.41901E-20	4.9204E-19
chr11	570265	570661	1.4465	4.52064E-14	5.40879E-13
chr11	572873	577516	2.2805	4.87978E-84	1.80676E-82
chr11	595716	597575	1.60036	2.208E-23	3.43954E-22
chr11	602482	604275	1.42589	4.09072E-13	4.74351E-12
chr11	605665	606140	1.27451	1.07263E-06	9.29951E-06
chr11	632983	633824	1.75867	1.84969E-33	3.54242E-32
chr11	659058	659791	2.17558	2.56448E-97	1.07473E-95
chr11	660131	660428	2.8322	1.5959E-136	9.0157E-135
chr11	662296	662886	3.20667	3.8282E-186	2.9376E-184
chr11	663201	664738	2.77627	2.9854E-146	1.7989E-144
chr12	1516	1834	3.31814	2.729E-269	3.3037E-267
chr12	3776	4113	3.46117	6.9502E-223	6.6834E-221
chr12	9308	9648	3.39936	9.4842E-214	8.6099E-212
chr12	51246	53394	2.50193	8.6896E-113	4.102E-111
chr12	103406	106082	3.76383	0	0
chr12	119054	122998	4.11909	0	0
chr12	127794	128264	1.37041	1.61503E-10	1.69531E-09
chr12	128879	129188	1.27249	5.9617E-06	4.93696E-05
chr12	133416	134580	1.28448	5.03211E-07	4.4459E-06
chr12	145815	147860	1.3681	1.44059E-10	1.51586E-09
chr12	148143	156722	15.3446	0	0
chr12	157029	158231	1.27511	7.51415E-07	6.57204E-06
chr12	158519	158842	1.44056	4.96935E-13	5.74513E-12
chr12	159244	162901	3.26433	1.5812E-211	1.4158E-209
chr12	164844	165562	1.25952	1.96106E-06	1.6739E-05
chr12	170014	170480	1.28398	4.91802E-07	4.3475E-06
chr12	171523	171843	1.2657	2.63566E-06	2.23229E-05
chr12	172310	172750	1.25979	3.76296E-06	3.15588E-05
chr12	173131	173484	1.31478	4.1262E-08	3.87026E-07
chr12	178726	179417	1.30156	1.35772E-07	1.23854E-06
chr12	180309	186226	1.93416	5.87625E-48	1.43384E-46

chr12	209667	211375	1.50167	1.59993E-17	2.13403E-16
chr12	218530	218899	1.30527	1.6138E-08	1.54614E-07
chr12	256981	257909	1.25007	3.36411E-06	2.83054E-05
chr12	306455	307166	1.36168	3.80908E-10	3.93469E-09
chr12	335844	337056	1.71466	3.748E-35	7.4114E-34
chr12	381758	382270	1.34322	3.59625E-09	3.5568E-08
chr12	458799	459602	1.29911	2.3388E-281	2.9785E-279
chr12	459881	460782	1.32532	0	0
chr12	467934	468770	1.2603	1.0186E-220	9.6383E-219
chr12	662163	663361	1.79102	2.45754E-35	4.87528E-34
chr12	733921	734909	1.84236	1.22829E-43	2.80737E-42
chr12	798744	800975	1.67492	6.87702E-30	1.23112E-28
chr12	802012	802482	1.25855	5.57892E-06	4.62828E-05
chr12	814662	816437	1.48278	5.08628E-16	6.48336E-15
chr12	821580	823258	1.46575	1.11892E-15	1.41124E-14
chr12	823508	824348	1.34537	8.08128E-09	7.86068E-08
chr12	835478	836133	1.25546	6.19598E-06	5.12389E-05
chr12	847661	848345	1.33451	1.21955E-08	1.17574E-07
chr12	865402	865691	1.40909	5.77564E-11	6.1776E-10
chr12	866205	869933	3.43639	4.5082E-228	4.4668E-226
chr12	881073	883850	1.78464	2.95053E-33	5.6273E-32
chr12	886466	890954	2.13848	1.8514E-64	5.60015E-63
chr12	891459	891783	1.35555	7.71223E-10	7.85887E-09
chr12	894651	896730	1.95638	4.11339E-50	1.0349E-48
chr12	952608	953196	1.45225	1.64437E-14	1.99618E-13
chr12	971077	972147	1.46077	1.6014E-14	1.94491E-13
chr12	998474	1001592	3.45609	2.7669E-256	3.1623E-254
chr12	1003831	1005295	1.62271	7.2929E-24	1.14895E-22
chr12	1006391	1008704	1.89787	3.0493E-45	7.14332E-44
chr12	1038361	1039896	1.6098	1.62368E-22	2.47742E-21
chr12	1063202	1063455	1.29606	9.44931E-19	1.30617E-17
chr12	1071931	1072438	3.29263	7.6736E-295	1.0304E-292
chr13	3510	3969	2.71585	2.8249E-122	1.4388E-120
chr13	8304	9453	1.80235	6.99037E-38	1.45211E-36
chr13	49387	50859	1.58374	3.4578E-22	5.236E-21
chr13	53310	55879	1.57271	1.62967E-22	2.48657E-21
chr13	57533	58067	1.48011	4.02346E-15	4.98425E-14

chr13	58356	58687	1.44621	2.60315E-13	3.03879E-12
chr13	73518	74845	1.70348	2.46037E-30	4.44427E-29
chr13	84919	86314	1.60725	3.90661E-22	5.90881E-21
chr13	107767	110409	1.94374	2.89534E-48	7.09578E-47
chr13	111217	113276	1.59025	5.17845E-23	7.9965E-22
chr13	144181	145563	1.97173	5.5616E-47	1.33721E-45
chr13	147357	149862	1.36839	1.60913E-10	1.68935E-09
chr13	152741	153602	1.31797	5.12153E-08	4.7809E-07
chr13	173700	174101	1.33456	2.26892E-09	2.2648E-08
chr13	188252	188744	1.38244	8.70362E-17	1.13606E-15
chr13	211558	213032	1.91498	3.85212E-48	9.42323E-47
chr13	237889	239679	2.24451	5.76235E-76	1.96834E-74
chr13	249922	251853	2.18155	2.06348E-73	6.87543E-72
chr13	254396	259139	8.03937	0	0
chr13	259838	262298	1.99879	3.434E-57	9.51919E-56
chr13	264041	270967	9.94909	0	0
chr13	274183	274519	1.25839	3.64477E-06	3.05907E-05
chr13	274947	280477	5.72722	0	0
chr13	293406	296517	3.83348	0	0
chr13	298639	301828	1.91958	4.72498E-49	1.17112E-47
chr13	316371	317323	1.4437	1.31795E-13	1.55346E-12
chr13	324234	324489	1.27088	1.94124E-06	1.65749E-05
chr13	326985	329832	1.52294	1.50522E-18	2.06871E-17
chr13	331063	332867	1.53829	2.07491E-18	2.83988E-17
chr13	338886	340582	1.82694	3.77398E-38	7.8759E-37
chr13	363064	363848	1.82511	4.23058E-35	8.35795E-34
chr13	403320	404574	1.42978	1.8681E-13	2.19079E-12
chr13	415196	416994	2.15919	1.07597E-74	3.63162E-73
chr13	442426	444383	1.79193	6.56448E-39	1.38867E-37
chr13	467422	468818	2.75026	1.6255E-134	9.0365E-133
chr13	488591	488887	1.28736	5.52077E-07	4.86542E-06
chr13	489139	491551	2.63545	7.8163E-131	4.2364E-129
chr13	498656	499391	1.62577	1.58088E-25	2.58464E-24
chr13	507468	507719	1.29335	7.38941E-07	6.46532E-06
chr13	519670	521548	1.96003	3.44112E-52	8.92072E-51
chr13	538231	539431	1.54513	3.66606E-19	5.12861E-18
chr13	549412	550116	2.07194	3.58096E-58	1.00485E-56

chr13	553135	555272	2.27777	4.3431E-81	1.56279E-79
chr13	576835	581030	2.79502	2.023E-154	1.2735E-152
chr13	634608	635444	1.37273	2.51519E-10	2.61806E-09
chr13	639290	642835	5.02128	0	0
chr13	665621	666837	1.59934	6.48933E-21	9.52138E-20
chr13	668408	669025	1.52335	2.75169E-16	3.53834E-15
chr13	669426	671640	2.89685	5.9293E-175	4.256E-173
chr13	676320	678422	1.76495	9.16854E-34	1.76563E-32
chr13	690295	690633	1.26189	2.5717E-06	2.17947E-05
chr13	697379	699385	1.87888	2.58345E-43	5.87489E-42
chr13	710916	714830	1.45852	6.72667E-15	8.2737E-14
chr13	717136	719058	1.37207	2.01697E-10	2.10858E-09
chr13	747974	749811	1.69268	1.08143E-26	1.81259E-25
chr13	761027	763710	2.54851	2.1777E-116	1.0568E-114
chr13	764588	765931	1.37876	4.3381E-11	4.66305E-10
chr13	824330	827940	2.50304	2.9309E-114	1.3996E-112
chr13	886944	890908	3.56046	1E-266	1.1967E-264
chr14	6079	6332	1.87259	1.07944E-43	2.46888E-42
chr14	37517	39668	1.66369	2.06063E-25	3.3597E-24
chr14	41356	43123	1.50693	8.93923E-18	1.20143E-16
chr14	69472	73066	2.81169	8.4528E-148	5.1404E-146
chr14	94156	96373	2.39109	3.29306E-92	1.32008E-90
chr14	124035	128153	2.60559	8.4528E-132	4.6132E-130
chr14	153371	157482	2.22134	1.25401E-78	4.40251E-77
chr14	157783	158896	1.29753	1.37832E-07	1.25696E-06
chr14	178931	180209	1.41829	8.53297E-13	9.78588E-12
chr14	194275	196869	2.90173	1.714E-153	1.0765E-151
chr14	213119	216192	1.86239	4.25305E-41	9.33254E-40
chr14	222022	223282	1.2936	3.6206E-07	3.22493E-06
chr14	253998	258122	2.77364	2.1281E-139	1.2246E-137
chr14	259623	262262	1.83316	1.90152E-39	4.06069E-38
chr14	291050	293673	3.3188	7.1285E-234	7.2778E-232
chr14	314147	317693	2.58208	9.9541E-119	4.9204E-117
chr14	318718	319104	1.30111	1.8906E-07	1.71096E-06
chr14	334392	335545	1.47958	4.39542E-16	5.61436E-15
chr14	343340	344181	2.23416	7.10232E-84	2.62482E-82
chr14	357740	362595	2.65572	6.6527E-129	3.5563E-127

chr14	388698	391367	2.43777	1.0483E-100	4.5061E-99
chr14	396316	398681	2.27308	2.75423E-80	9.83332E-79
chr14	401752	403036	1.5986	3.00815E-23	4.67089E-22
chr14	408435	409477	1.41064	5.36167E-11	5.74381E-10
chr14	409726	411133	1.82484	1.3627E-39	2.91743E-38
chr14	420433	421547	1.46233	1.56171E-14	1.89758E-13
chr14	435341	435857	1.25022	6.72233E-06	5.54728E-05
chr14	436876	438248	1.4164	1.20726E-12	1.37689E-11
chr14	468553	469580	1.66835	5.43E-26	8.96603E-25
chr14	469835	470638	2.00194	2.89468E-52	7.51104E-51
chr14	474749	476841	2.10548	6.74062E-67	2.09556E-65
chr14	477150	477470	1.31137	1.42646E-07	1.29981E-06
chr14	484860	487590	1.76163	1.08918E-33	2.09508E-32
chr14	492066	493764	1.72729	1.28588E-29	2.28981E-28
chr14	494584	496031	1.52212	8.20163E-17	1.07127E-15
chr14	504532	505731	1.50088	1.01414E-16	1.3213E-15
chr14	511843	512322	1.42794	1.50003E-12	1.7049E-11
chr14	512674	513266	1.30754	6.12083E-08	5.68971E-07
chr14	533545	536301	1.28337	4.84607E-07	4.28529E-06
chr14	539375	540401	1.44802	1.7402E-14	2.11106E-13
chr14	560978	561261	1.39555	2.40658E-11	2.61415E-10
chr14	569262	569777	1.30532	1.8706E-07	1.69321E-06
chr14	581795	582085	1.28314	1.0077E-06	8.74943E-06
chr14	582542	584333	1.75603	2.81903E-35	5.58599E-34
chr14	610615	618474	8.23552	0	0
chr14	618810	619434	1.4548	6.9215E-16	8.7882E-15
chr14	620028	622372	2.181	7.97995E-75	2.6965E-73
chr14	623276	625605	1.97027	3.48177E-54	9.27897E-53
chr14	625857	630699	10.3656	0	0
chr14	637642	646524	3.6515	2.0184E-281	2.5704E-279
chr14	647410	651315	3.04545	1.1482E-171	8.0724E-170
chr14	653335	655017	2.54516	7.6208E-111	3.54E-109
chr14	677009	678432	1.43151	6.56145E-14	7.81088E-13
chr14	678709	681227	2.60666	3.3037E-125	1.7179E-123
chr14	681498	681959	1.48502	1.32648E-14	1.61547E-13
chr14	710817	714541	1.95591	6.36796E-50	1.59809E-48
chr14	714790	717398	2.3495	6.41505E-85	2.39497E-83

chr14	748355	750653	2.28299	7.50931E-90	2.94171E-88
chr14	751074	752286	1.53451	2.24285E-22	3.41193E-21
chr14	783425	783883	2.72806	1.1429E-133	6.3241E-132
chr15	1145	1936	1.70698	1.92088E-43	4.37724E-42
chr15	11136	11527	1.74149	2.98882E-29	5.28689E-28
chr15	15410	15829	1.27612	4.6119E-06	3.84725E-05
chr15	22964	23326	1.67628	1.72902E-37	3.56533E-36
chr15	76983	78677	1.4948	8.10588E-16	1.02683E-14
chr15	89721	89990	1.2532	4.72988E-06	3.94158E-05
chr15	131539	134607	3.41537	7.0469E-256	8.0353E-254
chr15	148736	151489	2.03537	9.77012E-62	2.86418E-60
chr15	188177	194051	4.03537	0	0
chr15	195027	200114	1.99605	1.41286E-56	3.88508E-55
chr15	212956	213727	1.33017	5.88762E-09	5.76501E-08
chr15	238516	238828	1.34151	3.12363E-09	3.09863E-08
chr15	239325	240465	1.35565	5.91112E-10	6.05508E-09
chr15	248382	249020	1.43642	3.96826E-14	4.75773E-13
chr15	264837	266963	1.52243	1.07771E-18	1.4873E-17
chr15	269190	270227	1.3729	1.236E-10	1.30401E-09
chr15	279106	283737	2.67414	7.5162E-132	4.102E-130
chr15	293719	295010	1.61811	3.08887E-25	5.0188E-24
chr15	306697	313972	3.4004	6.9343E-243	7.4302E-241
chr15	317916	318898	1.36886	2.7928E-10	2.90188E-09
chr15	319389	338986	7.69298	0	0
chr15	344801	346826	1.37798	1.29748E-11	1.42364E-10
chr15	353080	354422	1.65373	3.38376E-24	5.37279E-23
chr15	360193	360771	1.31569	6.31582E-08	5.86665E-07
chr15	365546	367286	1.48032	1.61027E-15	2.02069E-14
chr15	369428	370971	1.52654	7.86683E-18	1.05925E-16
chr15	371670	376229	3.2904	1.4256E-223	1.3772E-221
chr15	388995	390042	1.3574	1.06038E-09	1.07409E-08
chr15	390380	391090	1.28144	5.12094E-07	4.52251E-06
chr15	391797	393777	2.03297	5.39138E-57	1.49073E-55
chr15	413775	415498	1.59347	2.02955E-21	3.01578E-20
chr15	450539	453270	2.14006	2.83335E-63	8.45668E-62
chr15	453988	456302	2.14152	1.50626E-68	4.7709E-67
chr15	468656	469738	1.56707	7.23602E-22	1.08718E-20

chr15	482073	484697	2.72616	6.8707E-137	3.8905E-135
chr15	489343	489693	1.3773	2.47508E-10	2.57763E-09
chr15	495626	499562	2.05509	5.86138E-58	1.64059E-56
chr15	507459	507718	1.27562	2.0773E-06	1.77068E-05
chr15	535544	536899	1.46699	3.00469E-15	3.73766E-14
chr15	537220	541594	2.72336	5.9704E-142	3.4995E-140
chr15	547731	549484	1.67639	6.77174E-28	1.16439E-26
chr15	562754	565627	2.0286	5.236E-58	1.46656E-56
chr15	580969	583313	2.7466	2.0749E-147	1.256E-145
chr15	588337	591181	2.28497	1.11455E-86	4.2335E-85
chr15	608563	608965	1.38642	2.47913E-11	2.69153E-10
chr15	612107	612965	1.31931	3.85762E-08	3.62485E-07
chr15	624489	627391	1.79407	1.25083E-36	2.54039E-35
chr15	635471	638433	1.90716	6.47739E-48	1.57979E-46
chr15	646402	646791	1.25942	6.99327E-06	5.76448E-05
chr15	650345	652115	1.5675	7.45418E-20	1.06341E-18
chr15	657744	661050	2.52794	2.2131E-115	1.0641E-113
chr15	663100	663570	1.27381	4.13371E-06	3.45836E-05
chr15	696334	699324	2.50287	7.2611E-117	3.5318E-115
chr15	699572	699907	1.54516	1.99848E-17	2.65889E-16
chr15	700242	700587	1.51971	4.22571E-16	5.40132E-15
chr15	715081	716760	1.66277	5.56929E-25	8.99498E-24
chr15	717989	719122	1.64187	7.64188E-24	1.20337E-22
chr15	725456	726508	2.05693	1.27938E-59	3.65511E-58
chr15	727569	728933	1.8542	3.05141E-37	6.26758E-36
chr15	729303	730529	2.3081	2.48428E-83	9.13482E-82
chr15	731476	731784	1.30463	2.94422E-07	2.63597E-06
chr15	735196	738077	2.34354	7.52835E-97	3.1434E-95
chr15	738372	739411	1.28706	3.79542E-07	3.37691E-06
chr15	751081	752859	2.00374	4.33012E-55	1.16762E-53
chr15	765955	767595	1.45632	4.82725E-16	6.15744E-15
chr15	776854	778497	2.21385	7.5544E-81	2.71206E-79
chr15	778904	779620	1.45919	8.75588E-14	1.03801E-12
chr15	787049	791410	2.11075	8.37336E-67	2.60076E-65
chr15	792849	795268	1.98232	1.19536E-58	3.3752E-57
chr15	797699	799776	1.70216	7.80369E-31	1.42266E-29
chr15	804371	805308	1.39537	2.09942E-11	2.28528E-10

chr15	815425	815768	1.28996	6.04269E-07	5.31398E-06
chr15	821083	823039	1.88761	1.41351E-43	3.22775E-42
chr15	827097	828059	1.50614	1.42824E-16	1.85183E-15
chr15	849499	850058	1.64796	1.3406E-48	3.30065E-47
chr15	855104	856032	2.08196	1.93687E-57	5.38642E-56
chr15	886832	891283	1.83092	4.54569E-43	1.02991E-41
chr15	917737	918986	1.88126	4.1735E-45	9.75439E-44
chr15	934840	939753	2.89469	3.1117E-177	2.2699E-175
chr15	965273	967042	1.7536	4.53524E-32	8.45668E-31
chr15	982724	986279	2.12729	1.17274E-65	3.59501E-64
chr15	990273	993676	2.08051	5.12979E-63	1.52651E-61
chr15	1011708	1013799	1.75052	6.36649E-31	1.16225E-29
chr15	1084856	1085396	3.07242	5.1642E-260	5.9841E-258
chr16	5987	6304	1.80694	6.76239E-37	1.38007E-35
chr16	50635	52239	2.4114	2.31526E-98	9.77913E-97
chr16	70333	71960	1.35747	2.99785E-10	3.11136E-09
chr16	89657	91255	1.87409	9.14324E-46	2.15924E-44
chr16	124589	125661	1.56517	5.11093E-19	7.12197E-18
chr16	139442	140703	1.69905	2.35288E-29	4.16869E-28
chr16	148277	149217	1.35651	4.05126E-10	4.17975E-09
chr16	167196	168778	2.18599	2.90737E-74	9.77012E-73
chr16	179888	184943	1.7381	5.4325E-32	1.01181E-30
chr16	219504	220493	1.34267	1.42331E-09	1.43347E-08
chr16	249587	251522	2.40049	7.7804E-110	3.581E-108
chr16	275625	278163	2.89722	5.7677E-172	4.0551E-170
chr16	283126	284360	1.61424	2.86682E-21	4.24424E-20
chr16	315129	315435	1.39103	9.33469E-11	9.8992E-10
chr16	315689	319186	2.08671	1.9893E-62	5.87895E-61
chr16	323023	324543	1.75815	2.08785E-35	4.14763E-34
chr16	331058	333296	2.42229	7.5509E-103	3.3037E-101
chr16	340527	342050	1.74676	3.11745E-31	5.724E-30
chr16	392559	392902	1.31555	1.90884E-08	1.8223E-07
chr16	393244	393621	1.29333	1.34571E-07	1.22789E-06
chr16	394567	399635	3.99888	0	0
chr16	422289	423108	1.35472	1.69946E-10	1.7823E-09
chr16	443261	446172	2.49054	5.7148E-112	2.6792E-110
chr16	446642	447311	1.77421	1.74622E-31	3.22181E-30

chr16	470937	474466	1.75555	1.10179E-33	2.11934E-32
chr16	499371	500780	1.53783	1.25083E-19	1.77296E-18
chr16	509463	512981	1.66133	1.84842E-28	3.21662E-27
chr16	513498	513868	1.26447	3.45979E-06	2.90904E-05
chr16	514316	514815	1.26738	2.22213E-06	1.89038E-05
chr16	524286	525657	2.12652	4.7326E-68	1.49108E-66
chr16	529649	537810	3.67613	4.5709E-285	5.902E-283
chr16	539089	543682	2.55243	2.7797E-115	1.3366E-113
chr16	544531	560442	6.58029	0	0
chr16	560829	566217	3.54106	4.8753E-261	5.6885E-259
chr16	576696	578274	1.73422	9.1622E-29	1.60435E-27
chr16	599120	601670	2.27772	1.17733E-81	4.25893E-80
chr16	618054	619810	1.9897	1.23623E-53	3.26964E-52
chr16	623837	624919	1.59701	3.37598E-24	5.3592E-23
chr16	636444	637933	1.96233	1.99434E-52	5.18681E-51
chr16	649233	652028	3.22848	3.3497E-189	2.6182E-187
chr16	660702	661440	1.29731	2.20232E-07	1.986E-06
chr16	673718	674552	1.30065	1.60613E-07	1.45949E-06
chr16	691988	692723	1.34374	1.14723E-09	1.16041E-08
chr16	702116	703669	1.67648	8.40814E-28	1.44278E-26
chr16	705926	709141	1.65311	1.03348E-26	1.73301E-25
chr16	710379	711856	1.94607	6.60846E-46	1.56387E-44
chr16	714268	717328	2.69492	1.6904E-138	9.6828E-137
chr16	733038	734303	1.98038	7.06155E-56	1.92398E-54
chr16	765788	769150	1.9045	9.65606E-46	2.27877E-44
chr16	773310	774328	1.31532	3.19654E-08	3.01613E-07
chr16	776257	776680	1.40263	1.34184E-11	1.4716E-10
chr16	779991	780362	1.38211	1.96743E-11	2.14417E-10
chr16	780672	781187	1.26588	1.48122E-06	1.27362E-05
chr16	781480	782219	1.27103	8.14498E-07	7.11001E-06
chr16	784050	784326	1.25215	5.89495E-06	4.88349E-05
chr16	802394	803227	1.39133	1.5758E-11	1.72401E-10
chr16	803471	804552	2.36981	8.7821E-99	3.72135E-97
chr16	808099	808458	1.23239	2.4454E-06	2.0751E-05
chr16	810752	812206	1.45443	5.79295E-15	7.13839E-14
chr16	815185	816364	1.77931	5.44252E-34	1.05269E-32
chr16	852745	853014	1.55209	9.09076E-76	3.09885E-74

chr16	862876	863446	1.3224	1.14269E-07	1.04691E-06
chr16	863700	865239	1.78002	3.287E-36	6.62674E-35
chr16	885263	886172	1.42391	2.57099E-12	2.89734E-11
chr16	886679	887028	1.44113	6.00759E-13	6.92628E-12
chr16	890432	890984	1.27243	8.66363E-07	7.55231E-06
chr16	892832	894690	1.61934	5.95662E-24	9.40373E-23
chr16	896132	897684	1.61922	7.46105E-23	1.14762E-21
chr16	915555	918178	1.78877	1.84417E-38	3.8699E-37
chr16	927980	928230	1.51215	1.00438E-20	1.46622E-19
chr16	944073	944555	4.48446	0	0
chr16	945986	946646	2.79703	0	0
chr2	879	1157	2.99748	2.6485E-301	3.6644E-299
chr2	1473	1950	3.07379	0	0
chr2	3130	3451	4.5955	0	0
chr2	5955	6226	1.97425	1.01836E-64	3.08887E-63
chr2	6775	7502	2.2939	6.5013E-233	6.6069E-231
chr2	44183	44527	1.25672	6.63101E-06	5.47369E-05
chr2	51117	51626	1.31469	4.45913E-08	4.17542E-07
chr2	66172	67896	2.3805	4.91247E-93	1.98472E-91
chr2	77927	80104	2.08666	2.68782E-64	8.11708E-63
chr2	104787	106194	1.86954	2.52464E-41	5.5616E-40
chr2	114874	116620	1.63282	1.44644E-24	2.31579E-23
chr2	118490	119849	1.59078	2.62482E-22	3.98474E-21
chr2	127233	128323	1.38882	5.09565E-11	5.46399E-10
chr2	148309	154231	3.13072	3.013E-197	2.466E-195
chr2	172918	173270	1.54611	1.53815E-17	2.05305E-16
chr2	173528	176312	3.24895	1.1272E-201	9.4842E-200
chr2	205829	206491	1.28286	4.47013E-07	3.96041E-06
chr2	209682	214683	5.20872	0	0
chr2	231397	233606	1.59746	1.6125E-23	2.52E-22
chr2	233869	235332	2.2681	2.30144E-92	9.2406E-91
chr2	235835	240905	11.3016	0	0
chr2	243434	251571	5.66308	0	0
chr2	252007	253846	1.48462	1.9476E-16	2.51478E-15
chr2	279102	286140	2.56514	8.6099E-134	4.7643E-132
chr2	304065	304943	1.53467	9.84238E-19	1.35988E-17
chr2	305332	305911	1.46012	6.20297E-14	7.38754E-13

chr2	318672	322234	3.26447	9.3972E-222	8.9743E-220
chr2	322520	323926	1.49373	1.21703E-17	1.62967E-16
chr2	333874	334377	1.6142	1.58562E-21	2.36265E-20
chr2	348741	350093	1.75024	3.16082E-32	5.91153E-31
chr2	351343	354776	1.46989	1.10078E-14	1.34462E-13
chr2	388169	389906	1.46096	3.33119E-15	4.13809E-14
chr2	393609	397667	4.01041	0	0
chr2	398085	398389	1.33066	7.10035E-08	6.57855E-07
chr2	398979	399361	1.29469	7.64874E-07	6.68683E-06
chr2	412239	413316	1.4723	8.87565E-16	1.12305E-14
chr2	413596	414090	1.61756	9.96093E-24	1.56459E-22
chr2	442132	444332	1.45078	2.29932E-14	2.77843E-13
chr2	459432	463348	1.7781	1.98016E-35	3.93641E-34
chr2	469542	471691	2.03036	9.38426E-84	3.46418E-82
chr2	480026	481560	1.39225	1.95659E-11	2.13275E-10
chr2	498796	499268	1.30647	1.7187E-07	1.5588E-06
chr2	511549	520558	4.06205	0	0
chr2	539922	542206	2.18471	6.79204E-70	2.18323E-68
chr2	549847	551272	1.35936	1.94603E-10	2.03624E-09
chr2	569540	570979	1.52619	3.70339E-18	5.03153E-17
chr2	572301	574557	2.43841	2.2131E-103	9.7275E-102
chr2	602895	604245	1.73664	1.81761E-32	3.41507E-31
chr2	607805	608090	1.2807	2.2183E-06	1.88717E-05
chr2	608482	608903	1.4262	4.15432E-12	4.64515E-11
chr2	609159	609777	1.32497	2.76713E-09	2.75138E-08
chr2	610044	610880	1.31701	1.22752E-08	1.18326E-07
chr2	631062	641029	3.37089	2.884E-250	3.1989E-248
chr2	641392	641920	1.44614	4.03088E-13	4.6752E-12
chr2	652588	652881	1.30852	8.84728E-08	8.15436E-07
chr2	654068	658700	2.56466	1.4289E-116	6.9343E-115
chr2	658968	659264	1.64457	1.81343E-23	2.83139E-22
chr2	659784	660189	1.4591	5.63119E-14	6.71893E-13
chr2	672614	677361	2.03513	2.2856E-61	6.67114E-60
chr2	689166	691112	1.80639	2.26986E-37	4.67197E-36
chr2	693483	696874	2.18176	1.0937E-69	3.50833E-68
chr2	704967	705682	1.33834	7.37972E-09	7.19118E-08
chr2	707057	709817	1.76092	9.01779E-33	1.70451E-31

chr2	731339	734354	2.12788	8.88792E-67	2.75931E-65
chr2	738969	739513	1.28082	7.62518E-07	6.66669E-06
chr2	739760	740310	1.27094	1.93361E-06	1.65105E-05
chr2	742200	742645	1.30437	1.43387E-07	1.30638E-06
chr2	743013	743365	1.29914	1.57972E-07	1.43602E-06
chr2	745533	746849	1.40338	8.23379E-12	9.10542E-11
chr2	750282	753582	2.70961	5.728E-143	3.3806E-141
chr2	762293	763112	1.31604	1.15569E-08	1.11548E-07
chr2	801851	803197	1.73905	8.31955E-42	1.84799E-40
chr3	1128	12004	4.39307	0	0
chr3	13067	13598	2.03641	3.3721E-58	9.46673E-57
chr3	13916	16704	2.1052	1.54277E-65	4.72389E-64
chr3	26843	28349	1.79375	8.35795E-36	1.67225E-34
chr3	28610	29443	1.30584	4.66595E-08	4.36486E-07
chr3	32804	33296	1.29027	8.58005E-07	7.48066E-06
chr3	35889	37852	2.19069	3.40643E-74	1.14419E-72
chr3	42191	46817	1.60126	9.16854E-24	1.44112E-22
chr3	53671	56833	2.66192	1.3459E-135	7.5509E-134
chr3	57247	58338	1.4479	4.39036E-13	5.08394E-12
chr3	59070	60649	1.34179	2.39409E-09	2.38671E-08
chr3	62032	67566	2.76301	2.3014E-137	1.3092E-135
chr3	69351	69977	1.2829	8.3207E-07	7.25989E-06
chr3	70539	73927	1.51983	1.26853E-17	1.69746E-16
chr3	74731	75154	1.4194	1.33937E-12	1.52546E-11
chr3	75508	75890	1.27192	2.1354E-06	1.81832E-05
chr3	80816	82543	1.87965	3.68129E-39	7.82168E-38
chr3	83150	83954	1.86057	9.42975E-44	2.15973E-42
chr3	90989	94280	4.24537	0	0
chr3	96605	104642	10.2059	0	0
chr3	104912	107230	1.91736	2.81968E-42	6.31103E-41
chr3	107761	117755	10.8005	0	0
chr3	129201	132080	6.01901	0	0
chr3	139157	139416	1.35856	2.20303E-09	2.20029E-08
chr3	143940	145382	1.65085	1.23367E-26	2.06586E-25
chr3	151369	151620	1.54193	1.00879E-18	1.39348E-17
chr3	188333	197175	4.26845	0	0
chr3	198711	199391	2.74801	6.2661E-211	5.5976E-209

chr3	200193	200774	2.20514	3.36047E-69	1.07201E-67
chr3	208538	209563	1.52008	5.84386E-17	7.66655E-16
chr3	209958	210472	1.43926	3.01648E-12	3.38922E-11
chr3	211773	213793	1.73786	5.23842E-29	9.21934E-28
chr3	214053	214607	1.36844	8.64032E-10	8.78375E-09
chr3	215197	216126	1.27401	1.05114E-06	9.11759E-06
chr3	217154	222678	2.24459	1.26911E-79	4.50194E-78
chr3	230600	235617	3.89687	0	0
chr3	239446	240506	1.3759	4.05695E-11	4.36697E-10
chr3	240872	241847	1.39638	1.20948E-11	1.32926E-10
chr3	242155	245411	1.56104	2.88403E-20	4.15815E-19
chr3	248164	249131	1.44207	1.45412E-12	1.65348E-11
chr3	249437	250399	1.56243	5.4063E-20	7.73927E-19
chr3	251095	252374	1.32537	4.21551E-08	3.95239E-07
chr3	256950	258575	1.42672	1.34462E-13	1.58416E-12
chr3	258867	260077	2.56672	3.2734E-108	1.4894E-106
chr3	260497	260740	1.43272	2.77843E-12	3.1268E-11
chr3	268708	269075	1.5282	1.01695E-27	1.74221E-26
chr3	271726	272793	1.27764	8.07012E-07	7.04628E-06
chr3	274550	275470	1.28763	4.78939E-07	4.23633E-06
chr3	275780	278785	1.83227	1.22096E-41	2.70396E-40
chr3	285753	288620	2.00075	7.11705E-54	1.88886E-52
chr3	289834	293039	2.0281	1.50176E-61	4.39339E-60
chr3	293821	294114	2.26139	1.45044E-72	4.79181E-71
chr3	294613	297220	2.03041	5.25896E-52	1.35957E-50
chr3	297467	301779	1.86501	1.48354E-43	3.3861E-42
chr3	314374	314727	1.3322	3.81154E-15	4.72498E-14
chr4	2142	2785	1.43295	2.9744E-14	3.58014E-13
chr4	24249	27076	3.07278	3.54E-196	2.884E-194
chr4	52859	55004	2.09902	4.89779E-68	1.54277E-66
chr4	91929	92874	1.29402	8.7265E-08	8.04563E-07
chr4	95254	96144	1.56146	3.89583E-20	5.59887E-19
chr4	117122	118142	1.73058	1.59919E-28	2.78676E-27
chr4	118607	119251	1.71183	3.55468E-30	6.4003E-29
chr4	140581	140981	1.29911	1.92513E-07	1.74157E-06
chr4	141294	141557	1.24362	7.41413E-06	6.10141E-05
chr4	142728	147073	2.5823	7.8524E-129	4.1976E-127

chr4	192207	195948	2.73956	3.4914E-138	1.9953E-136
chr4	203187	206504	3.59958	1.2246E-257	1.406E-255
chr4	226326	226642	1.30227	4.39076E-07	3.89189E-06
chr4	226966	227223	1.5147	1.86294E-16	2.40713E-15
chr4	232382	233674	1.27968	2.23702E-06	1.90261E-05
chr4	235498	236585	1.52266	2.38013E-19	3.34734E-18
chr4	253410	254281	1.61829	8.22811E-25	1.32434E-23
chr4	256271	256741	1.37123	1.19264E-10	1.25878E-09
chr4	263012	263671	1.33478	3.69786E-09	3.65536E-08
chr4	279798	280433	1.37635	2.81255E-10	2.92146E-09
chr4	292883	293746	1.29766	1.55278E-07	1.41208E-06
chr4	297586	298730	1.50112	8.16394E-18	1.0985E-16
chr4	333252	333562	1.32881	9.64029E-09	9.34136E-08
chr4	335690	336131	1.34615	1.2479E-08	1.20246E-07
chr4	353510	355617	1.70974	4.49987E-32	8.39267E-31
chr4	375947	380440	1.75766	4.85289E-34	9.39291E-33
chr4	399917	402430	3.27464	1.4421E-226	1.4158E-224
chr4	414064	415556	1.63684	5.87084E-25	9.47764E-24
chr4	439327	442431	4.32271	0	0
chr4	442906	444200	1.62179	8.33489E-23	1.28056E-21
chr4	444627	450182	13.1196	0	0
chr4	450487	451492	4.13377	0	0
chr4	451898	452144	1.36702	3.15718E-09	3.13105E-08
chr4	455485	457536	1.80214	8.21864E-39	1.7354E-37
chr4	468645	471745	2.57267	1.0715E-119	5.3456E-118
chr4	472949	476287	2.99034	1.7498E-168	1.205E-166
chr4	476567	477428	1.57631	3.24265E-21	4.79402E-20
chr4	477759	479378	2.52199	1.9055E-108	8.6896E-107
chr4	479672	480912	1.70363	1.01672E-28	1.77869E-27
chr4	492402	493578	1.60789	3.17687E-25	5.15941E-24
chr4	581744	582958	1.36054	2.83426E-10	2.94374E-09
chr4	583682	585736	2.22629	1.23396E-80	4.42079E-79
chr4	605554	608507	2.33292	3.32736E-90	1.30768E-88
chr4	638845	639142	1.45644	1.20781E-13	1.42561E-12
chr4	639437	642956	3.72645	1.135E-270	1.3836E-268
chr4	659492	662813	2.16386	5.99515E-80	2.13304E-78
chr4	677392	677995	1.64654	3.26437E-26	5.41627E-25

chr4	697181	699822	1.86972	2.67917E-43	6.09116E-42
chr4	700631	701328	1.39189	6.37676E-12	7.07946E-11
chr4	710234	715055	4.10971	0	0
chr4	733143	735955	1.99997	5.85464E-59	1.6592E-57
chr4	762494	765052	2.20439	2.99502E-79	1.05803E-77
chr4	765918	766940	1.25557	5.42325E-06	4.50257E-05
chr4	776721	778613	1.3971	1.54028E-11	1.68566E-10
chr4	778962	782545	1.75172	6.40914E-34	1.23823E-32
chr4	782820	783921	1.50854	1.50695E-16	1.95254E-15
chr4	785014	786688	1.48306	1.30677E-15	1.64475E-14
chr4	791162	792979	1.7229	1.58782E-30	2.8774E-29
chr4	824414	827257	2.20765	5.18203E-78	1.80801E-76
chr4	827551	833571	1.94134	1.17625E-48	2.89868E-47
chr4	833910	834478	1.26548	1.37079E-06	1.18092E-05
chr4	837315	837893	1.31374	8.73796E-08	8.05601E-07
chr4	838796	840022	1.45895	3.85212E-15	4.77419E-14
chr4	840347	841310	1.94998	8.4489E-48	2.05636E-46
chr4	843346	844644	1.49446	2.29721E-16	2.96006E-15
chr4	863525	865569	2.92109	1.094E-160	7.1779E-159
chr4	898326	902700	4.48729	0	0
chr4	920801	922457	1.52936	1.00879E-18	1.39348E-17
chr4	934725	935427	1.29334	2.22608E-07	2.00692E-06
chr4	936761	938183	2.09885	8.3888E-63	2.49E-61
chr4	962208	964549	1.80418	1.91426E-39	4.0879E-38
chr4	965029	966237	1.40304	2.82618E-12	3.1798E-11
chr4	967532	968394	1.37573	1.39749E-10	1.47143E-09
chr4	993829	995198	1.46715	1.52792E-15	1.91867E-14
chr4	1023766	1025168	1.66727	4.48745E-28	7.7464E-27
chr4	1025563	1026420	1.41428	3.75491E-11	4.04846E-10
chr4	1040526	1047265	3.44068	1.6106E-238	1.6827E-236
chr4	1066136	1069562	2.30661	2.14882E-84	7.98362E-83
chr4	1077664	1078594	1.30784	9.63541E-08	8.86115E-07
chr4	1118766	1121796	2.08271	2.16521E-68	6.84857E-67
chr4	1143502	1146213	1.80234	2.5787E-37	5.30274E-36
chr4	1146485	1148314	1.68544	3.41901E-28	5.91562E-27
chr4	1151774	1152127	1.33066	7.10035E-08	6.57855E-07
chr4	1152381	1152699	1.37713	9.44409E-10	9.58539E-09

chr4	1188621	1189202	1.36025	3.03739E-10	3.15166E-09
chr4	1190106	1192756	3.47414	1.7219E-268	2.0797E-266
chr4	1247643	1248836	1.29876	9.98665E-08	9.17698E-07
chr4	1253828	1255898	2.06965	1.99526E-62	5.89658E-61
chr4	1271745	1273522	1.59145	1.44777E-23	2.26517E-22
chr4	1282328	1286468	3.32988	1.6368E-227	1.6144E-225
chr4	1286773	1287069	1.25797	4.60564E-06	3.84211E-05
chr4	1311309	1312718	1.46241	6.23878E-15	7.68069E-14
chr4	1313129	1316424	2.66655	1.1041E-133	6.1094E-132
chr4	1344564	1344965	1.26053	4.07258E-06	3.40824E-05
chr4	1378952	1381003	2.8567	6.0954E-159	3.9537E-157
chr4	1397103	1400198	1.99175	8.99498E-57	2.4797E-55
chr4	1403695	1404273	1.23787	8.07495E-06	6.6272E-05
chr4	1406677	1408431	1.58381	8.62383E-22	1.2933E-20
chr4	1414321	1415103	1.67288	1.86037E-27	3.16957E-26
chr4	1423830	1425600	2.01351	2.67732E-57	7.4319E-56
chr4	1437670	1438732	1.37781	1.62237E-10	1.70251E-09
chr4	1452128	1453527	1.84503	4.29141E-39	9.10752E-38
chr4	1454964	1456811	1.62618	1.28056E-24	2.05258E-23
chr4	1523611	1523856	1.67605	5.28324E-25	8.53886E-24
chr4	1524259	1524991	2.32079	8.3176E-173	5.8884E-171
chr4	1525758	1526259	3.24603	2.9992E-282	3.8371E-280
chr5	379	1018	2.13051	3.53834E-60	1.01789E-58
chr5	1314	2182	3.62834	0	0
chr5	2640	2913	2.46879	1.9231E-132	1.0544E-130
chr5	28989	30262	1.51507	1.56171E-18	2.14536E-17
chr5	78081	79444	2.08426	2.46263E-58	6.92468E-57
chr5	94862	97830	1.54767	4.95108E-21	7.2845E-20
chr5	102240	103012	1.79145	4.59515E-34	8.9002E-33
chr5	115744	117317	3.85065	2.1478E-307	3.0549E-305
chr5	118701	119095	1.26336	4.7338E-06	3.94466E-05
chr5	133886	135556	2.80311	6.5917E-133	3.6392E-131
chr5	135981	138862	2.57111	3.0974E-126	1.6218E-124
chr5	143097	158681	7.10976	0	0
chr5	160028	160308	1.24793	8.6459E-06	7.08239E-05
chr5	160557	169018	3.68405	2.421E-293	3.2359E-291
chr5	185727	188241	2.90448	3.2211E-166	2.1827E-164

chr5	188615	190548	2.66197	1.122E-134	6.2661E-133
chr5	205114	205940	1.46881	1.33537E-13	1.57362E-12
chr5	206743	207044	1.97862	1.21283E-47	2.9451E-46
chr5	207869	208123	1.36083	3.71878E-09	3.6757E-08
chr5	215779	219481	4.30336	0	0
chr5	232289	236311	2.32388	1.98884E-93	8.06678E-92
chr5	255286	257068	1.90729	9.06776E-44	2.07683E-42
chr5	257427	257882	1.33736	4.58839E-09	4.51534E-08
chr5	273689	275490	1.65533	6.19441E-26	1.02164E-24
chr5	301180	301466	1.29463	4.19614E-07	3.72332E-06
chr5	307714	310131	1.76748	2.7842E-37	5.72137E-36
chr5	323601	325341	1.40976	7.34683E-13	8.44306E-12
chr5	331280	332936	1.75976	1.66265E-35	3.30902E-34
chr5	338983	339613	1.33202	1.48361E-08	1.42443E-07
chr5	365035	370161	2.74857	7.6033E-136	4.2756E-134
chr5	419118	421901	3.09838	1.9588E-196	1.5959E-194
chr5	424655	426734	1.73916	2.12276E-33	4.06069E-32
chr5	449858	450392	1.38562	4.79954E-11	5.15146E-10
chr5	456951	459459	2.11467	1.92353E-68	6.08695E-67
chr5	466243	466621	1.31604	3.23981E-08	3.05619E-07
chr5	475148	477000	1.82274	6.82025E-41	1.49211E-39
chr5	520373	521582	1.49341	9.31751E-16	1.17815E-14
chr5	528082	531674	2.79574	4.1976E-147	2.541E-145
chr5	555108	556939	1.27742	8.20087E-07	7.15748E-06
chr5	574913	575340	2.91281	1.5596E-243	1.6711E-241
chr6	906	1318	3.54775	0	0
chr6	38488	39369	1.43169	3.11602E-13	3.6266E-12
chr6	41929	43604	1.64929	2.57573E-26	4.28253E-25
chr6	62119	64066	2.01501	1.74582E-54	4.67089E-53
chr6	65726	71629	6.89874	0	0
chr6	72039	73002	1.3295	1.28404E-08	1.23652E-07
chr6	76638	87531	2.5332	1.7258E-119	8.5704E-118
chr6	93087	93472	1.57078	9.73419E-20	1.3842E-18
chr6	96901	99240	1.84823	2.55682E-41	5.63248E-40
chr6	99789	101305	1.46536	4.32713E-15	5.3555E-14
chr6	101621	101930	1.42917	2.39056E-12	2.69712E-11
chr6	128450	136506	11.112	0	0

chr6	143938	162161	8.86476	0	0
chr6	162438	162706	1.33451	3.36767E-08	3.17388E-07
chr6	163185	163456	1.32184	7.93506E-08	7.33179E-07
chr6	164436	177579	7.30752	0	0
chr6	189946	190929	1.46818	1.58745E-14	1.92841E-13
chr6	191299	191673	1.47881	4.27268E-15	5.2881E-14
chr6	192994	194792	1.79356	5.04778E-38	1.05099E-36
chr6	195632	197231	1.86203	3.03669E-40	6.57052E-39
chr6	197512	202696	2.78407	1.3521E-147	8.2035E-146
chr6	214183	219076	4.03282	0	0
chr6	231223	232414	1.32247	1.1183E-08	1.08039E-07
chr6	236836	237101	1.31136	4.68921E-08	4.38601E-07
chr6	237363	240389	1.70659	2.34747E-30	4.24033E-29
chr6	248468	248901	1.45225	1.36144E-14	1.6573E-13
chr7	551	4503	2.51601	1.2589E-230	1.2618E-228
chr7	7534	7975	1.48948	1.83443E-25	2.99502E-24
chr7	52026	52789	1.59038	2.64484E-23	4.11244E-22
chr7	53041	53345	1.54161	3.05703E-18	4.1639E-17
chr7	100808	104038	4.46922	0	0
chr7	116967	117745	1.46804	1.08118E-14	1.32069E-13
chr7	136443	137385	1.43281	1.74864E-13	2.05258E-12
chr7	137640	140541	2.75601	4.7973E-141	2.7925E-139
chr7	144980	146118	1.44547	2.18223E-13	2.55329E-12
chr7	163121	164085	1.51742	2.81125E-17	3.72392E-16
chr7	169457	173756	4.86816	0	0
chr7	174034	176120	1.82633	2.83727E-40	6.14186E-39
chr7	181699	182679	2.90756	9.2045E-178	6.7298E-176
chr7	219015	222700	3.60701	9.2257E-270	1.1194E-267
chr7	231768	233291	1.4106	7.53182E-13	8.65366E-12
chr7	240338	242290	2.13428	5.0269E-68	1.58307E-66
chr7	252086	253032	1.3437	3.60288E-09	3.56328E-08
chr7	258175	263193	2.6633	2.4099E-138	1.3772E-136
chr7	273397	275322	1.69442	1.56964E-28	2.73527E-27
chr7	296691	299168	2.27758	3.95185E-83	1.45011E-81
chr7	300047	302011	1.41706	9.60506E-13	1.09926E-11
chr7	341027	342358	1.61855	2.93359E-24	4.66337E-23
chr7	348717	351058	1.64501	3.3335E-25	5.41128E-24

chr7	360667	361500	1.25035	9.37066E-06	7.65826E-05
chr7	378975	380336	1.51621	1.00925E-17	1.35425E-16
chr7	387533	391737	2.28585	7.91225E-86	2.98057E-84
chr7	392036	392571	1.37706	9.36483E-11	9.92727E-10
chr7	392815	397072	1.97645	5.27473E-52	1.36364E-50
chr7	400290	400901	1.42155	1.8281E-11	1.99503E-10
chr7	405834	408680	1.85712	3.76184E-42	8.40234E-41
chr7	430755	433011	2.92705	6.7143E-166	4.5499E-164
chr7	436617	437344	1.54998	4.32016E-20	6.20012E-19
chr7	439769	440848	1.38251	9.32825E-11	9.89191E-10
chr7	441142	446758	1.6569	2.98951E-28	5.17845E-27
chr7	447068	448553	1.47489	6.74994E-16	8.57038E-15
chr7	448996	449558	1.4961	1.82264E-15	2.28297E-14
chr7	450256	452030	1.33835	1.69609E-09	1.70228E-08
chr7	452931	458587	1.4688	7.5631E-16	9.58959E-15
chr7	458839	461428	1.54716	3.46418E-19	4.84953E-18
chr7	462859	465407	1.52921	6.36209E-19	8.83894E-18
chr7	466035	471239	8.04293	0	0
chr7	473033	474777	2.27811	1.03777E-86	3.94366E-85
chr7	475720	478355	2.05657	4.59515E-57	1.27174E-55
chr7	486769	499354	8.62408	0	0
chr7	500722	510406	5.22329	0	0
chr7	517752	522196	3.20596	1.4555E-199	1.2106E-197
chr7	530141	532787	3.94213	0	0
chr7	533158	534031	1.75661	1.99894E-31	3.68383E-30
chr7	542141	542640	1.31622	7.25471E-08	6.71645E-07
chr7	542947	544771	1.5507	3.72907E-21	5.50427E-20
chr7	552056	555215	4.33799	0	0
chr7	585246	587666	2.22943	2.78227E-77	9.63163E-76
chr7	603657	605124	1.86047	6.46547E-43	1.46184E-41
chr7	611488	614716	4.05742	0	0
chr7	629427	632885	2.36018	2.58167E-93	1.04616E-91
chr7	674655	675327	1.55555	3.287E-18	4.47301E-17
chr7	675617	676217	1.57804	3.38844E-20	4.87753E-19
chr7	680116	684331	3.66589	0	1.6482E-307
chr7	723012	726364	3.00355	2.6002E-171	1.8197E-169
chr7	727849	729472	1.51376	2.77715E-16	3.56944E-15

chr7	744577	744982	1.31376	1.37927E-07	1.25777E-06
chr7	747364	749693	1.69268	1.74181E-30	3.155E-29
chr7	750614	751694	1.34901	1.53102E-09	1.53993E-08
chr7	777776	778565	1.47384	1.20893E-15	1.52335E-14
chr7	789998	792207	1.451	3.91471E-15	4.85065E-14
chr7	804459	805564	1.31911	1.83362E-08	1.75202E-07
chr7	841137	841823	1.32278	1.41039E-08	1.3555E-07
chr7	844842	845259	1.42031	1.37025E-11	1.50231E-10
chr7	846558	847470	1.41688	2.86022E-11	3.09649E-10
chr7	849688	851580	1.59187	3.72392E-20	5.35427E-19
chr7	859968	860267	1.37434	1.19281E-10	1.25895E-09
chr7	934747	938665	2.34702	2.99778E-93	1.21423E-91
chr7	940441	941360	1.42402	4.13523E-13	4.79402E-12
chr7	946631	950455	3.06434	1.2417E-180	9.247E-179
chr7	954360	955510	1.34034	2.70689E-09	2.69259E-08
chr7	960799	961505	1.38569	8.57828E-11	9.10961E-10
chr7	962722	963536	1.24355	4.37341E-06	3.65317E-05
chr7	1006269	1006606	1.32282	2.03109E-08	1.93638E-07
chr7	1014302	1015251	1.36991	2.54947E-10	2.65302E-09
chr7	1037342	1038087	1.27225	1.26206E-06	1.08951E-05
chr7	1038962	1040556	1.50356	2.0017E-16	2.58404E-15
chr7	1056874	1057787	1.69489	1.40864E-27	2.40602E-26
chr7	1083780	1084220	2.26315	3.6559E-109	1.6788E-107
chr7	1084631	1084880	1.99579	1.07696E-50	2.73275E-49
chr7	1088979	1089400	3.15156	4.7534E-280	6.0256E-278
chr8	753	1090	2.39228	7.45933E-86	2.81061E-84
chr8	1622	2150	2.31073	1.4622E-276	1.8281E-274
chr8	2495	2908	3.50116	7.2611E-229	7.2277E-227
chr8	3511	3986	2.37774	2.46661E-84	9.15799E-83
chr8	45100	46407	2.02711	1.48457E-57	4.13428E-56
chr8	58620	63096	8.17597	0	0
chr8	66324	66780	1.40139	7.2277E-12	8.0094E-11
chr8	69538	74532	4.45334	0	0
chr8	75810	80704	2.77039	6.9984E-129	3.7325E-127
chr8	81478	83010	1.77785	9.76113E-32	1.81009E-30
chr8	92410	92965	1.86994	2.58821E-39	5.51442E-38
chr8	99951	100985	1.3909	4.24033E-11	4.56173E-10

chr8	103628	113032	7.90595	0	0
chr8	113381	114452	1.32859	4.10743E-09	4.05191E-08
chr8	117783	120718	6.73328	0	0
chr8	120964	121295	1.99599	1.91955E-48	4.71628E-47
chr8	122989	124957	1.69328	4.66445E-28	8.05008E-27
chr8	130087	130730	1.35964	1.42177E-09	1.43199E-08
chr8	134295	140445	4.22355	0	0
chr8	140746	143122	1.66926	4.10582E-27	6.94544E-26
chr8	153256	153658	1.28074	9.99563E-07	8.68041E-06
chr8	154568	160889	4.82718	0	0
chr8	161283	161964	2.60678	1.8621E-109	8.5704E-108
chr8	162335	162624	1.26722	6.30275E-06	5.20991E-05
chr8	183012	185895	2.25535	3.16009E-83	1.16091E-81
chr8	191257	192351	1.47609	6.03949E-14	7.1978E-13
chr8	192652	192981	1.62878	4.01236E-22	6.06597E-21
chr8	195992	200637	2.24947	7.08925E-74	2.37356E-72
chr8	210785	213080	1.59665	1.65082E-24	2.63876E-23
chr8	214521	215316	1.47609	6.03949E-14	7.1978E-13
chr8	246633	249744	2.91344	1.7579E-180	1.3062E-178
chr8	250672	253665	2.25947	5.49667E-83	2.01465E-81
chr8	257128	259201	2.11787	6.09537E-70	1.9602E-68
chr8	313670	314514	1.57897	3.06196E-20	4.41266E-19
chr8	322371	327941	1.99811	6.09537E-53	1.59735E-51
chr8	328864	330040	1.44713	8.36951E-14	9.93116E-13
chr8	334474	335488	1.32604	1.63117E-08	1.56243E-07
chr8	338880	340251	1.51892	2.97509E-18	4.05415E-17
chr8	343640	344217	1.34587	7.15418E-09	6.97638E-08
chr8	347768	351859	2.00217	1.78033E-54	4.76321E-53
chr8	352151	352815	2.0256	1.56135E-51	4.01051E-50
chr8	353654	354732	1.33866	2.45612E-09	2.44748E-08
chr8	361269	366882	2.10325	6.95024E-72	2.28034E-70
chr8	392008	393093	1.8462	1.27057E-38	2.67424E-37
chr8	393618	395251	1.56509	9.68947E-20	1.37784E-18
chr8	398481	399907	1.57727	6.00067E-21	8.81049E-20
chr8	408975	411891	2.35593	1.02802E-93	4.18119E-92
chr8	412986	414504	1.56852	3.26964E-21	4.83393E-20
chr8	420624	421512	1.28142	1.13713E-06	9.8451E-06

chr8	423309	425748	1.94207	3.59253E-49	8.91867E-48
chr8	472554	478448	2.60315	4.8753E-109	2.2387E-107
chr8	513756	514689	1.62507	7.18621E-25	1.15824E-23
chr8	514950	516005	2.00595	8.4879E-54	2.25061E-52
chr8	519843	520468	1.29648	1.69887E-07	1.54174E-06
chr8	520927	521827	1.7417	6.25317E-33	1.18522E-31
chr8	522312	523400	1.28734	2.08439E-07	1.88209E-06
chr8	554199	555331	2.17388	0	0
chr8	555641	556232	1.71909	7.8705E-210	6.9823E-208
chr8	559401	559691	3.36887	6.0117E-239	6.2951E-237
chr8	560456	560883	3.45753	2.4155E-222	2.3121E-220
chr9	79	2590	2.641	0	0
chr9	2836	3200	2.53853	5.4325E-254	6.1376E-252
chr9	7532	8547	2.22674	9.1411E-220	8.6099E-218
chr9	14699	15895	1.4256	8.6836E-28	1.4897E-26
chr9	16207	17369	1.35698	5.88844E-20	8.4217E-19
chr9	46276	46698	1.26685	2.49983E-06	2.12012E-05
chr9	49426	52931	2.99212	3.3343E-166	2.2594E-164
chr9	55446	55773	1.28178	1.58161E-06	1.35769E-05
chr9	56672	56996	1.26703	4.24473E-06	3.54862E-05
chr9	79905	80740	1.94134	2.01419E-48	4.94538E-47
chr9	81103	81605	1.3954	1.60887E-10	1.68908E-09
chr9	87320	87873	1.46883	9.19602E-16	1.16305E-14
chr9	105038	107073	2.07546	5.41128E-61	1.57217E-59
chr9	125180	126824	1.57874	6.44021E-23	9.91973E-22
chr9	128982	129399	1.29646	5.23733E-07	4.62275E-06
chr9	132446	133584	1.27889	1.10629E-06	9.58407E-06
chr9	143052	145379	1.92191	6.16737E-47	1.48184E-45
chr9	154682	154941	1.2478	7.37819E-06	6.07295E-05
chr9	155304	157404	1.93962	4.66122E-54	1.23994E-52
chr9	162258	163613	1.5812	2.66563E-21	3.94912E-20
chr9	185876	186952	1.36269	5.94347E-10	6.08751E-09
chr9	195114	195958	1.29241	3.59335E-07	3.20103E-06
chr9	216752	218040	2.03148	1.80094E-58	5.07224E-57
chr9	228560	230594	1.7509	1.28499E-34	2.51536E-33
chr9	233529	234353	1.37891	1.62166E-10	1.70188E-09
chr9	251632	252366	1.44537	3.09885E-14	3.72735E-13

chr9	263654	266349	3.4244	5.7544E-245	6.223E-243
chr9	266695	267519	1.35247	1.13154E-08	1.0929E-07
chr9	279003	280406	1.87893	4.93628E-43	1.11789E-41
chr9	280789	281288	1.32209	4.01976E-08	3.77251E-07
chr9	294828	298973	6.07828	0	0
chr9	302198	303391	1.37703	3.48418E-11	3.75959E-10
chr9	316610	317128	1.62723	2.56567E-22	3.89673E-21
chr9	318004	319306	2.56772	7.5509E-119	3.7411E-117
chr9	326178	331978	4.01743	0	0
chr9	334781	335048	1.29066	8.88853E-07	7.74355E-06
chr9	335407	336875	2.06119	1.8412E-54	4.9238E-53
chr9	339646	358902	9.8899	0	0
chr9	361451	366468	3.52414	2.5586E-265	3.0409E-263
chr9	366740	367293	1.33104	8.34795E-09	8.1126E-08
chr9	372436	372819	1.30852	1.58358E-07	1.43946E-06
chr9	376307	379143	1.78762	1.56387E-40	3.40017E-39
chr9	379495	379840	1.28218	9.86597E-07	8.57077E-06
chr9	384823	386242	1.53162	4.9705E-18	6.72822E-17
chr9	389152	390852	2.34825	1.7865E-111	8.3368E-110
chr9	419633	420023	1.41222	5.94566E-12	6.60998E-11
chr9	432236	432703	1.44339	1.10739E-17	1.48423E-16
chr9	433026	433681	1.86147	8.72168E-38	1.80842E-36

Table S2. List of yeast strains used in the study.

**Strain ID**	**Genotype**	**MAT**
GMY023	*ho::LYS2 lys2 ura3 leu2::hisG his3::hisG trp1::hisG RPL13A-2xFKBP12-TRP1 fpr1::kanMX4 tor1-1::HIS3 REC8-FRB-HYG*	*MATalpha*
GMY024	*ho::LYS2 lys2 ura3 leu2::hisG his3::hisG trp1::hisG RPL13A-2xFKBP12-TRP1 fpr1::kanMX4 tor1-1::HIS3 REC8-FRB-HYG*	*MATa*
GMY026	*ho::LYS2, lys2, LEU2, ura3, trp1::hisG , his3, RPL13A-2xFKBP12::TRP1, fpr1Δ::KanmX4, tor1-1::HIS3, ndt80Δ::TRP1 REC8-FRB-GFP-NAT*	*MATa*
GMY028	*ho::LYS2, lys2, URA3, LEU2, his3::hisG, trp1::hisG, RPL13A-2xFKBP12::TRP1, tor1-1::HIS3, ndt80Δ::TRP1, fpr1Δ::KanmX4 REC8-FRB-GFP-NAT*	*MATalpha*

GMY034	*ho::LYS2 lys2 ura3 leu2::hisG his3::hisG trp1::hisG RPL13A-2xFKBP12-TRP1 fpr1::kanMX4 tor1-1::HIS3 REC8-FRB-HYG ho::LYS2 lys2 ura3 leu2::hisG his3::hisG trp1::hisG RPL13A-2xFKBP12-TRP1 fpr1::kanMX4 tor1-1::HIS3 REC8-FRB-HYG*	*Diploid*
GMY035	*ho::LYS2, lys2, LEU2, ura3, trp1::hisG , his3, RPL13A-2xFKBP12::TRP1, fpr1Δ::KanmX4, tor1-1::HIS3, ndt80Δ::TRP1 REC8-FRB-GFP-NAT ho::LYS2, lys2, URA3, LEU2, his3::hisG, trp1::hisG, RPL13A-2xFKBP12::TRP1, tor1-1::HIS3, ndt80Δ::TRP1, fpr1Δ::KanmX4 REC8-FRB-GFP-NAT*	*Diploid*
GMY214	*ho::LYS2 lys2 ura3 leu2::hisG his3::hisG trp1::hisG RPL13A-2xFKBP12-TRP1 fpr1::kanMX4 tor1-1::HIS3 REC8-FRB-HYG leu2::TetR-GFP::LEU2*	*MATalpha*
GMY216	*ho::LYS2 lys2 ura3 leu2::hisG his3::hisG trp1::hisG RPL13A-2xFKBP12-TRP1 fpr1::kanMX4 tor1-1::HIS3 REC8-FRB-HYG ho::LYS2 lys2 ura3 leu2::hisG his3::hisG trp1::hisG RPL13A-2xFKBP12-TRP1 fpr1::kanMX4 tor1-1::HIS3 REC8-FRB-HYG SPC42-NeonGreen::URA3*	*Diploid*
GMY218	*ho::LYS2 lys2 ura3 leu2::hisG his3::hisG trp1::hisG RPL13A-2xFKBP12-TRP1 fpr1::kanMX4 tor1-1::HIS3 REC8-FRB-HYG leu2::TetR-GFP::LEU2 CENV::TetO-HIS3*	*MATa*
GMY219	*ho::LYS2 lys2 ura3 leu2::hisG his3::hisG trp1::hisG RPL13A-2xFKBP12-TRP1 fpr1::kanMX4 tor1-1::HIS3 REC8-FRB-HYG leu2::TetR-GFP::LEU2 ho::LYS2 lys2 ura3 leu2::hisG his3::hisG trp1::hisG RPL13A-2xFKBP12-TRP1 fpr1::kanMX4 tor1-1::HIS3 REC8-FRB-HYG leu2::TetR-GFP::LEU2 CENV::TetO-HIS3*	*Diploid*
GMY603	*ho::Lys2, lys2, ura3, leu2::hiSGY, his3::hiSGY, trp1::hiSGY rec8Δ::hphMX6 SPC42-neon green:URA3*	*MATalpha*
GMY604	*ho::Lys2, lys2, ura3, leu2::hiSGY, his3::hiSGY, trp1::hiSGY rec8Δ::hphMX6 SPC42-neon green:URA3*	*MATa*
GMY605	*ho::Lys2, lys2, ura3, leu2::hiSGY, his3::hiSGY, trp1::hiSGY rec8Δ::hphMX6 SPC42-neon green: URA3 ho::Lys2, lys2, ura3, leu2::hiSGY, his3::hiSGY, trp1::hiSGY rec8Δ::hphMX6 SPC42-neon green:URA3*	*Diploid*

Table S3. List of Primers used in the study.

Sr no.	Primer ID	Description	Template	Sequence
1	GM007	*REC8-FRB* tagging (Forward)	pFA6a-FRB-hphMX6	TAACTAAAGATCTTAAATTGAGAAGAGAGGACGAAATAATTGTATATGCCCGGATCCCCGGGTTAATTAA
2	GM008	*REC8-FRB* tagging (Reverse)	pFA6a-FRB-hphMX6	CAGCGGCTAGTAACCGCTGTCCTCATATGGAAGGAGAAAATAAAAATTCAGAATTCGAGCTCGTTTAAAC
3	GM009	Diagnostic primer for *REC8-FRB* (Forward)	*S. cerevisiae* genomic DNA	GACAGATAGCCGCCAGTGCC
4	GM010	Diagnostic primer for *REC8-FRB* (Reverse)	*S. cerevisiae* genomic DNA	GCATATTCGGCCCGGATCTCGG
5	GM218	*SPC42*-NeonGreen tagging (Reverse)	pTSK509	GAACGCTTTAAGAATGTGCCATACTCCTTAACTGCTTTTTTAAATCATCAGCGTCCATCTTTACAGTCCT
6	GM219	Diagnostic primer for *SPC42*-NeonGreen tagging (Forward)	*S. cerevisiae* genomic DNA	CCCAATCCAGTCGCGATTAC
7	GM220	Diagnostic primer for *SPC42*-NeonGreen tagging (Reverse)	*S. cerevisiae* genomic DNA	GTTTCCGGCTTCTGTTGG
12	GM229	*SPC42-NeonGreen* tagging (Forward)	pTSK509	CTGAAAATAATATGTCAGAAACATTCGCAACTCCCACTCCCAATAATCGAGATCTGTACGACGATGACGATAAG
13	GM233	*CENIII* (C3.1) Primer (Forward)	*S. cerevisiae* genomic DNA (ChIP-qPCR)	GATCAGCGCCAAACAATATGG
14	GM234	*CENIII* (C3.1) Primer (Reverse)	*S. cerevisiae* genomic DNA (ChIP-qPCR)	AACTTCCACCAGTAAACGTTT

15	GM235	*CENIV* (C4.1) Primer (Forward)	*S. cerevisiae* genomic DNA (ChIP-qPCR)	GCTTGCAAAAGGTCACATGC
16	GM236	*CENIV* (C4.1) Primer (Reverse)	*S. cerevisiae* genomic DNA (ChIP-qPCR)	GAGCAGGTTTTATGTTTCGG
17	GM237	Peri-centromeric (P3.2) Primer (Forward)	*S. cerevisiae* genomic DNA (ChIP-qPCR)	CATCTTTGAAAAGTTCATCAAGG
18	GM238	Peri-centromeric (P3.2) Primer (Reverse)	*S. cerevisiae* genomic DNA (ChIP-qPCR)	CGATAACAAAGCATGGTATGGC
19	GM239	Peri-centromeric (P3.3) Primer (Forward)	*S. cerevisiae* genomic DNA (ChIP-qPCR)	GTCAACGAGTCCTCTCTGGC
20	GM240	Peri-centromeric (P3.3) Primer (Reverse)	*S. cerevisiae* genomic DNA (ChIP-qPCR)	TTTACTGGTGGAAGTTTTGCTC
21	GM241	Peri-centromeric (P4.1) Primer (Forward)	*S. cerevisiae* genomic DNA (ChIP-qPCR)	TAGTGCTTTCTCCACCATTT
22	GM242	Peri-centromeric (P4.1) Primer (Reverse)	*S. cerevisiae* genomic DNA (ChIP-qPCR)	ACAGAACGTCATACGAATCC
23	GM243	Peri-centromeric (P4.2) Primer (Forward)	*S. cerevisiae* genomic DNA (ChIP-qPCR)	CTTCTGGCTTGTTCACAACT
24	GM244	Peri-centromeric (P4.2) Primer (Reverse)	*S. cerevisiae* genomic DNA (ChIP-qPCR)	GGCAATTGTAGGTGGACTAA
25	GM245	Arm region (A4.1) Primer (CAR site) (Forward)	*S. cerevisiae* genomic DNA (ChIP-qPCR)	GAAAGCGACCAGCTAGATTA
26	GM246	Arm region (A4.1) Primer (CAR site) (Reverse)	*S. cerevisiae* genomic DNA (ChIP-qPCR)	CAAACGCTTTAACACACAAG

27	GM247	Arm region (A4.2) Primer (non-CAR site) (Forward)	*S. cerevisiae* genomic DNA (ChIP-qPCR)	GTCGATGGTTTCATTCAGAT
28	GM248	Arm region (A4.2) Primer (non-CAR site) (Reverse)	*S. cerevisiae* genomic DNA (ChIP-qPCR)	CAATGGAGAGAGTGGATGTT
29	GM555	Forward Primer *CDC5*	cDNA (RT-qPCR)	CGCAGACCTCGTAGGAAAAG
30	GM556	Reverse Primer *CDC5*	cDNA (RT-qPCR)	TAATTTGGAAACAGCGAGCA
31	GM557	Forward Primer *CLB1*	cDNA (RT-qPCR)	TGAAGAGAAGGGGGTAAACG
32	GM558	Reverse Primer *CLB1*	cDNA (RT-qPCR)	AGGTGAAGTCATCGGCTCTC
33	GM559	Forward Primer *HOP1*	cDNA (RT-qPCR)	TTGCTCCAAACAATGCTCAC
34	GM560	Reverse Primer *HOP1*	cDNA (RT-qPCR)	CTCCCTTCTCCAACCAATCC
35	GM561	Forward Primer *IME1*	cDNA (RT-qPCR)	ATGACACAACCACCGATCAA
36	GM562	Reverse Primer *IME1*	cDNA (RT-qPCR)	TCGTTTCCTTGGAAATAGGC
37	GM563	Forward Primer *IME2*	cDNA (RT-qPCR)	AAGCGCAATTTCCTTTATCG
38	GM564	Reverse Primer *IME2*	cDNA (RT-qPCR)	GCTGGGATTGCCAGTATGAA
39	GM567	Forward Primer *MRE11*	cDNA (RT-qPCR)	ATGGTTGTACAGTCCGGTGA
40	GM568	Reverse Primer *MRE11*	cDNA (RT-qPCR)	TGCCGAATACGGGAATAGAA
41	GM569	Forward Primer *MSH5*	cDNA (RT-qPCR)	CCTTCGATTTTGACGAGGAG
42	GM570	Reverse Primer *MSH5*	cDNA (RT-qPCR)	CAGTTGGGTTTGCTTCCATT
43	GM571	Forward Primer *NDT80*	cDNA (RT-qPCR)	ATTGACAGAGGGTTCGACCA

44	GM572	Reverse Primer *NDT80*	cDNA (RT-qPCR)	CGTGTCGTCGTCATCATTCT
45	GM573	Forward Primer *REC8*	cDNA (RT-qPCR)	AAAGCAACTCCACTGGAAACA
46	GM574	Reverse Primer *REC8*	cDNA (RT-qPCR)	GCACAAGCAGGTGATTTAGG
47	GM575	Forward Primer *RED1*	cDNA (RT-qPCR)	GCCGTTAAAATCACCGATTC
48	GM576	Reverse Primer *RED1*	cDNA (RT-qPCR)	ATTGAGGGACACTGCTGAGG
49	GM577	Forward Primer *SPO11*	cDNA (RT-qPCR)	CGCAGATGTTTTGGCTCATA
50	GM578	Reverse Primer *SPO11*	cDNA (RT-qPCR)	GGATTGCGCATCTTTTCAAC
51	GM579	Forward Primer *SPO13*	cDNA (RT-qPCR)	TTGAGCAGCCTCCAAAATCT
52	GM580	Reverse Primer *SPO13*	cDNA (RT-qPCR)	TGGATGGGAATGGCATAGTT
53	GM581	Forward Primer *UME6*	cDNA (RT-qPCR)	GCTAGACAAGGCACGCTCTC
54	GM582	Reverse Primer *UME6*	cDNA (RT-qPCR)	TGTAGGATGGCGGGAGTTAG
55	GM583	Forward Primer *ZIP1*	cDNA (RT-qPCR)	TGGGGATCCTAACGATTCTG
56	GM584	Reverse Primer *ZIP1*	cDNA (RT-qPCR)	TGCATTATCCTGTTGCTGCT

Table S4. List of plasmids used in the study.

Sr. No.	Plasmid ID	Purpose	Source
1	pFA6a-FRB-hphMX6 (VS p601)	To tag the gene of interest with FRB at the endogenous locus, hygromycin-resistant	Gift from Viji Subramanian (21)
2	pTSK509	To tag the gene of interest with the Neon green, URA3 marker	This study
3	FRB-GFP-natMX6 (pLD116)	To tag the gene of interest with FRB-GFP at the endogenous locus, nourseothricin-resistant	Susan Forsburg's lab, University of

			Southern California, USA (47)
4	p4211	TetR tagging, LEU2 marker	Kim Nasmyth lab, University of Oxford, UK (59)
5	p3939	Tet O tagging, HIS3 marker	Kim Nasmyth lab, University of Oxford, UK (59)
